# A Facile One-Pot Construction of Succinimide-Fused Spiro[Pyrrolidine-2,3′-Oxindoles] via 1,3-Dipolar Cycloaddition Involving 3-Amino Oxindoles and Maleimides

**DOI:** 10.3390/molecules23030582

**Published:** 2018-03-05

**Authors:** Lunqiang Jin, Feng Liang

**Affiliations:** The State Key Laboratory of Refractories and Metallurgy, Coal Conversion and New Carbon Materials Hubei Key Laboratory, School of Chemistry & Chemical Engineering, Wuhan University of Science and Technology, Wuhan 430081, China; jinlqchem123@163.com

**Keywords:** 1,3-dipolar cycloaddition, azomethine ylide, one pot synthesis, maleimide, succinimide-fused spiro[pyrrolidine-2,3′-oxindole]

## Abstract

Increasing interests have been invested in the development of synthetic strategies toward the construction of spiro[pyrrolidine-2,3′-oxindole], which is the core structural skeleton in some compounds with diverse biological activities. In this work, an efficient diastereoselective 1,3-dipolar cycloaddition reaction of azomethine ylides generated in situ from 3-amino oxindoles and aldehydes with maleimides has been described. The protocol provides a facile and efficient access to structurally diverse succinimide-fused spiro[pyrrolidine-2,3′-oxindole] compounds in good to high yields (up to 93%) with moderate to excellent diastereoselectivities (up to >95:5). The relative stereochemistry of cycloaddition products has been assigned by X-ray diffraction analysis.

## 1. Introduction

**Figure 1:**
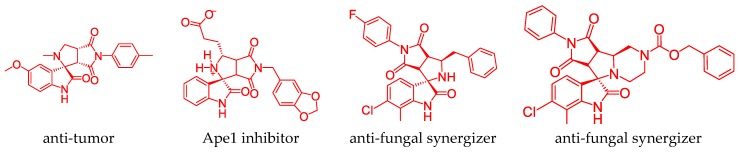
Representative bioactive succinimide-fused spiro[pyrrolidine-2,3′-oxindoles].

**Scheme 1:**
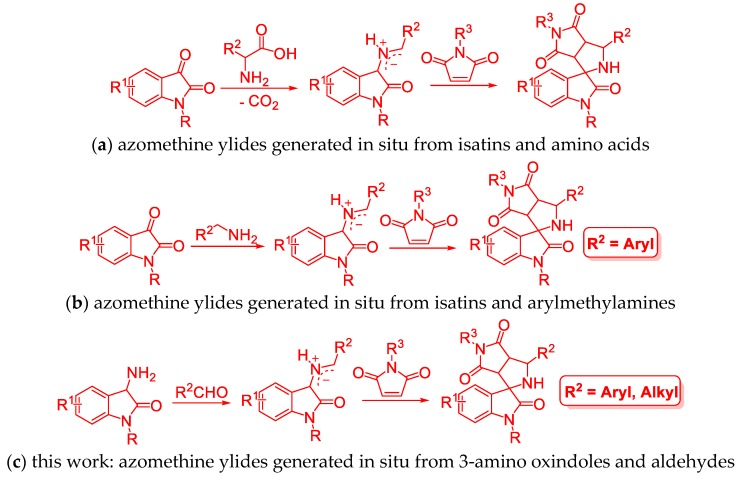
Strategies for the construction of succinimide-fused spiro[pyrrolidine-2,3′-oxindoles].

The spiro[pyrrolidine-2,3′-oxindole] has been identified as the core structural skeleton in some unnatural compounds with diverse biological activities [1,2,3,4,5,6]. As a subset of spiro[pyrrolidine-2,3′-oxindole], succinimide-fused spiro-[pyrrolidine-2,3′-oxindole] has been attracting more attention due to the recent discovery of some important biological activities such as anti-tumor [7], Ape1 inhibitor [8], and anti-fungal synergizer [9] (Figure 1). As a consequence, much more attention has been paid to developing synthetic strategies toward the construction of this spirocyclic structure [2,7,9,10,11,12,13,14,15,16]. Among these reported approaches, most studies have focused on 1,3-dipolar cycloaddition of azomethine ylides generated in situ via decarboxylative condensation of isatins with amino acids (Scheme 1a) [7,9,13,14,15].

Although Zhao et al. developed a 1,3-dipolar cycloaddition of azomethine ylides generated in situ from isatins and arylmethylamines with maleimides (Scheme 1b) [16], an urgent necessity is still in need to enrich the synthetic methodologies of spiro[pyrrolidine-2,3′-oxindole]. 1,3-dipolar cycloaddition of azomethine ylides derived from 3-aminooxindole with other alkenes such as nitroalkene derivatives [17,18], α and β unsaturated imines [19], and ketones [20] were investigated. However, to our best knowledge, no azomethine ylides derived from aminoindolones were used in this kind of 1,3-dipolar cycloaddition. As well, azomethine ylides have been widely investigated in synthesizing spiro[pyrrolidine-2,3′-oxindoles] [17,18,19,20,21]. Herein, we describe a facile and efficient strategy for accessing to succinimide-fused spiro[pyrrolidine-2,3′-oxindoles] by a one-pot three-component 1,3-dipolar cycloaddition reaction of azomethine ylides generated in situ from 3-amino oxindoles **1** and aldehydes **2** with maleimides **3** in the presence of triethylamine (TEA, Scheme 1c), which is a supplement to previous work.

## 2. Results and Discussion

**Scheme 2:**
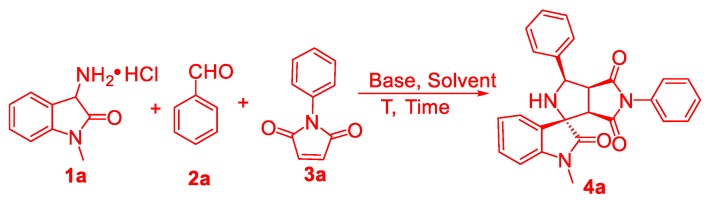
The three-component reaction of 3-amino-1-methylindolin-2-one hydrochloride **1a**, benzaldehyde **2a,** and *N*-phenylmaleimide **3a**. Prolonging reaction time would benefit the reaction.

We commenced our studies with the three-component reaction of 3-amino-1-methylindolin-2-one hydrochloride **1a**, **2a**, and *N*-phenylmaleimide **3a** (Scheme 2) as model substrates for surveying the reaction parameters, and the results are summarized in Table 1. Initially, the reaction was performed in the presence of 1 equivalent of weak inorganic base NaHCO_3_ and the desired product **4a** could be obtained in 60% yield with 74:26 diasteromeric excess (dr, Table 1, entry 1). Other two weak inorganic bases, K_2_CO_3_ and KF/Al_2_O_3_, did not provide better results (Table 1, entries 2 and 3). When the strong base NaOH was employed, only trace product was detected (Table 1, entry 4). A further study showed that organic base TEA could afford **4a** in 68% yield and 83:17 dr (Table 1, entry 6), and prolonging reaction time would benefit the reaction yield (Table 1, entry 7). Subsequently, a series of solvents were also screened. As seen from Table 1, with chlorinated alkane-type solvents, ether-type solvents, alcohol-type solvents, toluene or acetonitrile, the current strategy could afford the desired product **4a** in various yields and diastereoselectivities (Table 1, entries 8–16). In terms of diastereoselectivity, CH_2_Cl_2_ was selected as the optimal reaction solvent (Table 1, entry 8). When the reaction temperature was increased to reflux, up to 86% yield could be obtained without erosion in diastereoselectivity (Table 1, entry 17). Scaling up reaction did not result in the loss of the reactivity and diastereoselectivity (Table 1, entry 18).

**Scheme 3:**
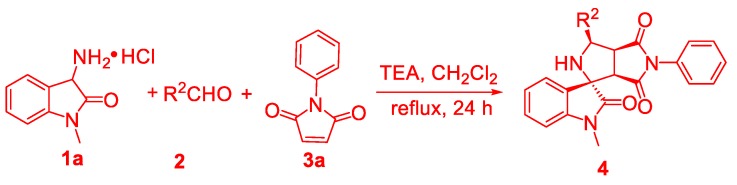
A variety of aldehyde substrates **2** were investigated under optimized reaction conditions.

Under optimum conditions, a variety of aldehyde substrates **2** were firstly investigated (Scheme 3). As shown in Table 2, all tested aldehydes underwent the reaction smoothly to afford the corresponding products with good to excellent results. Both electron-withdrawing and electron-donating substituents on the aryl ring of R^2^ groups could be well tolerated (Table 2, entries 2–19). It was shown that the positions of the substituents on the aryl ring of R^2^ groups seem to play a significant influence on the reaction results. The *ortho*- and *para*-substituents exhibit more beneficial impact on reaction yield and diastereoselectivity than *meta*-substituents (Table 2, entries 2 and 4 vs. entry 3; entries 5 and 7 vs. entry 6; entries 9 and 11 vs. entry 10; entries 15 and 17 vs. entry 16; entry 18 vs. entry 19). The aldehyde adorned with 2-naphthyl group could be well performed, affording 93% yield and 94:6 dr (Table 2, entry 20). Notably, as demonstrated by the examples with 2-furyl and 2-thienyl substituents, heteroaryl aldehydes **2u** and **2v** could be also well accommodated, giving 94:6 and 93:7 dr values, respectively (Table 2, entries 21 and 22). In addition, aliphatic aldehydes could be tolerated albeit with moderate diastereoselectivities (Table 2, entries 23 and 24).

**Scheme 4:**
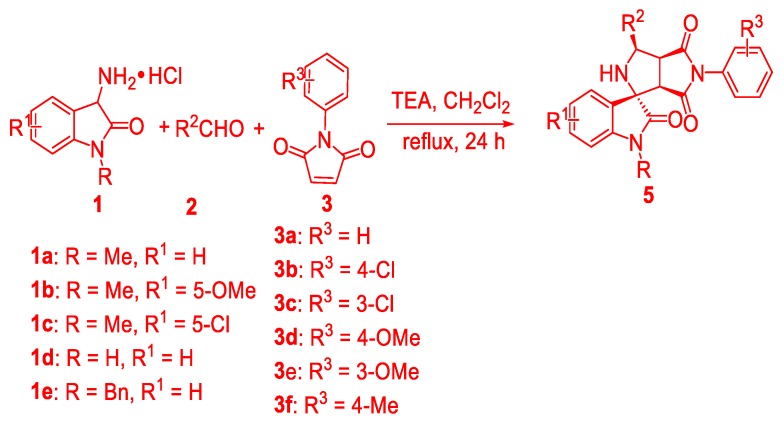
Screening of a series of 3-amino oxindoles **1** and maleimides **3**.

To extend the utility of this procedure, we then screened a series of 3-amino oxindoles **1** and maleimides **3** (Scheme 4). As can be seen from Table 3, the electronic property of the substituent R^1^ on aromatic ring of 3-amino oxindole seems to show significant influence on the diastereoselectivity of the reaction, and electron-donating group gave better dr value than electron-withdrawing group (Table 3, entry 1 vs. entry 3, entry 2 vs. entry 4). Additionally, the *N*-protecting group R of 3-amino oxindole has also been found to have a major impact on the reaction result. When methyl-substituted **1a** was replaced with benzyl-substituted **1e**, the diastereoselectivity of the reaction was decreased from 88:12 to 83:17 (Table 2, entry 1 vs. Table 3, entry 6). Unprotected **1d** came to the worst results (Table 3, entry 5). Next, to further validate the compatibility of this strategy, the scope of maleimides **3** was also explored. It was found that substrates **3** with either electron-withdrawing or electron-donating substituents R^3^ could be amenable to this reaction system (Table 3, entries 8–13).

**Figure 2:**
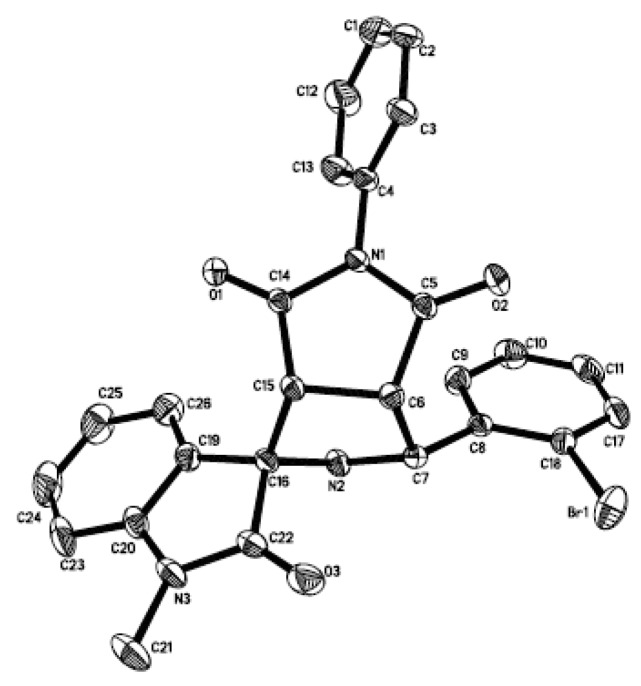
X-ray crystal structure of product **4k**. All H atoms have been omitted for clarity.

The relative configuration of 1,3-dipolar cycloaddition product **4k** was established by X-ray diffraction analysis (Figure 2) [22], the relevant data shown in Supplementary Materials and the relative configurations of other succinimide-fused spiro[pyrrolidine-2,3′-oxindole] products were assigned by analogy.

## 3. Materials and Methods 

### 3.1. Experimental

All reactions were carried out in reaction tubes with magnetic stirring and no special precautions were taken to exclude air from the reaction vessels. TLC was performed on pre-coated silica gel plates (Qingdao Marine Chemistry Company, Qingdao, China). Column chromatography was carried out with silica gel (200–300 mesh, Qingdao Marine Chemistry Company, Qingdao, China) eluting with ethyl acetate and petroleum ether. NMR spectra were recorded with a Bruker Avance II 400 NMR spectrometer (Bruker Biospin, Fällanden, Switzerland). Chemical shifts are reported in parts per million (ppm) downfield from TMS (Aladdin, Shanghai, China) with the solvent resonance as the internal standard. Coupling constants (*J*) are reported in Hz and refer to apparent peak multiplications. High Resolution Mass Spectrometer (HRMS) was recorded on a Bruker micrOTOF-Q II mass spectrometer (Bruker Daltonics Inc., Billerica, Massachusetts, MA, USA). X-ray diffraction analysis was recorded with a Bruker Apex-II spectrometer (Bruker AXS, Karlsruhe, Germany).

### 3.2. General Procedure forthe Preparation of Succinimide-Fused Spiro[Pyrrolidine-2,3′-Oxindoles] ***4*** and ***5***

3-Amino oxindoles **1** (0.2 mmol), aldehydes **2** (0.2 mmol) and TEA (0.2 mmol) were put into an ordinary test tube equipped with a magnetic stirring bar and then sealed in the air. Then, CH_2_Cl_2_ (1 mL) was added. After being stirred at room temperature for 30 min, maleimides **3** (0.22 mmol) and CH_2_Cl_2_ (1 mL) were added and the resulting mixture was stirred at reflux for 24 h. The crude reaction mixture was directly purified by flash column chromatography on silica gel (petroleum ether/ethyl acetate = 7:1–3:1) to give the correspondingsuccinimide-fused spiro[pyrrolidine-2,3′-oxindole] products **4** or **5**. All the products were confirmed by ^1^H-NMR, ^13^C-NMR and HRMS spectroscopic analysis. The diastereomeric ratio was determined by crude NMR analysis.

*1-Methyl-3′,5′-diphenyl-2′,3′,3a′,6a′-tetrahydro-4′H-spiro[indoline-3,1′-pyrrolo[3,4-c]pyrrole]-2,4′,6′(5′H)-trione* (**4a**). White solid, 71.9 mg, 85% yield. 88:12 dr. ^1^H-NMR (400 MHz, CDCl_3_): δ 7.56 (d, *J* = 7.2 Hz, 2H), 7.48–7.43 (m, 2H), 7.41–7.36 (m, 4H), 7.36–7.31 (m, 2H), 7.24 (d, *J* = 7.4 Hz, 2H), 7.14 (t, *J* = 7.6 Hz, 1H), 6.90 (d, *J* = 7.8 Hz, 1H), 5.83 (d, *J* = 8.8 Hz, 1H), 4.03 (t, *J* = 8.4 Hz, 1H), 3.55 (d, *J* = 7.9 Hz, 1H), 3.25 (s, 3H); ^13^C-NMR (100 MHz, CDCl_3_): δ 178.8, 174.1, 173.5, 143.7, 138.0, 130.2, 129.1, 128.5, 128.4, 128.3, 127.3, 127.0, 126.2, 125.4, 122.8, 108.4, 68.0, 60.8, 50.9, 49.5, 26.2; HRMS (ESI): *m*/*z* calcd for C_26_H_21_NaN_3_O_3_^+^ [M + Na]^+^ 446.1481, found 446.1493.

*3′-(4-Fluorophenyl)-1-methyl-5′-phenyl-2′,3′,3a′,6a′-tetrahydro-4′H-spiro[indoline-3,1′-pyrrolo[3,4-c]pyrrole]-2,4′,6′(5′H)-trione* (**4b**). White solid, 70.6 mg, 80% yield. 81:19 dr. ^1^H-NMR (400 MHz, CDCl_3_): δ 7.51 (dd, *J* = 7.7, 5.8 Hz, 2H), 7.45 (t, *J* = 7.6 Hz, 2H), 7.38 (dd, *J* = 13.7, 7.0 Hz, 2H), 7.32 (d, *J* = 7.5 Hz, 1H), 7.22 (d, *J* = 7.6 Hz, 2H), 7.12 (t, *J* = 7.5 Hz, 1H), 7.06 (t, *J* = 8.4 Hz, 2H), 6.89 (d, *J* = 7.8 Hz, 1H), 5.80 (d, *J* = 8.7 Hz, 1H), 3.97 (t, *J* = 8.3 Hz, 1H), 3.53 (d, *J* = 7.9 Hz, 1H), 3.23 (s, 3H); ^13^C-NMR (100 MHz, CDCl_3_): δ 178.7, 174.2, 173.5, 143.7, 130.3, 129.2, 128.9, 128.8, 128.6, 126.9, 126.1, 125.3, 122.8, 115.4, 115.2, 108.5, 67.9, 60.0, 50.8, 49.4, 26.2; HRMS (ESI): *m*/*z* calcd for C_26_H_20_FN_3_NaO_3_^+^ [M + Na]^+^ 442.1567, found 442.1579.

*3′-(3-Fluorophenyl)-1-methyl-5′-phenyl-2′,3′,3a′,6a′-tetrahydro-4′H-spiro[indoline-3,1′-pyrrolo[3,4-c]pyrrole]-2,4′,6′(5′H)-trione* (**4c**). White solid, 61.8 mg, 70% yield. 62:38 dr. ^1^H-NMR (400 MHz, CDCl_3_): δ 7.45 (t, *J* = 7.6 Hz, 2H), 7.39 (dd, *J* = 15.4, 7.8 Hz, 3H), 7.32 (t, *J* = 9.0 Hz, 3H), 7.22 (d, *J* = 7.6 Hz, 2H), 7.13 (t, *J* = 7.5 Hz, 1H), 7.00 (dd, *J* = 10.4, 5.5 Hz, 1H), 6.89 (d, *J* = 7.8 Hz, 1H), 5.81 (d, *J* = 8.8 Hz, 1H), 4.01 (t, *J* = 8.4 Hz, 1H), 3.54 (d, *J* = 7.9 Hz, 1H), 3.24 (s, 3H); ^13^C-NMR (100 MHz, CDCl_3_): δ 178.7, 174.0, 173.4, 143.7, 130.3, 129.9, 129.2, 128.7, 127.0, 126.2, 123.2, 122.9, 115.1, 114.1, 113.9, 108.5, 67.9, 60.1, 50.8, 49.4, 26.2; HRMS (ESI): *m*/*z* calcd for C_26_H_20_FN_3_NaO_3_^+^[M + Na]^+^ 442.1567, found 442.1576.

*3′-(2-Fluorophenyl)-1-methyl-5′-phenyl-2′,3′,3a′,6a′-tetrahydro-4′H-spiro[indoline-3,1′-pyrrolo[3,4-c]pyrrole]-2,4′,6′(5′H)-trione* (**4d**). White solid, 79.4 mg, 90% yield. 92:8 dr. ^1^H-NMR (400 MHz, CDCl_3_): δ 7.57 (t, *J* = 7.1 Hz, 1H), 7.43–7.38 (m, 4H), 7.30 (d, *J* = 5.9 Hz, 1H), 7.20 (d, *J* = 7.6 Hz, 2H), 7.16–7.08 (m, 4H), 6.89 (d, *J* = 7.8 Hz, 1H), 6.01 (d, *J* = 8.4 Hz, 1H), 4.13 (t, *J* = 8.2 Hz, 1H), 3.61 (d, *J* = 8.0 Hz, 1H), 3.24 (s, 3H); ^13^C-NMR (100 MHz, CDCl_3_): δ 178.6, 174.0, 173.5, 143.8, 130.3, 129.5, 128.6, 127.0, 126.9, 126.7, 126.2, 122.9, 115.2, 115.0, 108.5, 67.8, 54.7, 51.1, 48.0, 26.2; HRMS (ESI): *m*/*z* calcd for C_26_H_20_FN_3_NaO_3_^+^ [M + Na]^+^ 442.1567, found 442.1573.

*3′-(4-Chlorophenyl)-1-methyl-5′-phenyl-2′,3′,3a′,6a′-tetrahydro-4′H-spiro[indoline-3,1′-pyrrolo[3,4-c]pyrrole]-2,4′,6′(5′H)-trione* (**4e**). White solid, 72.2 mg, 79% yield. 81:19 dr. ^1^H-NMR (400 MHz, CDCl_3_): δ 7.47 (t, *J* = 8.4 Hz, 5H), 7.38–7.30 (m, 4H), 7.24 (d, *J* = 7.5 Hz, 2H), 7.14 (t, *J* = 7.5 Hz, 1H), 6.90 (d, *J* = 7.8 Hz, 1H), 5.80 (d, *J* = 8.7 Hz, 1H), 4.00 (t, *J* = 8.3 Hz, 1H), 3.56 (d, *J* = 7.9 Hz, 1H), 3.25 (s, 3H); ^13^C-NMR (100 MHz, CDCl_3_): δ 178.7, 174.0, 173.4, 143.7, 136.5, 133.9, 131.7, 130.3, 129.2, 128.7, 128.6, 126.9, 126.1, 125.3, 122.9, 108.5, 67.9, 60.1, 50.8, 49.4, 26.2; HRMS (ESI): *m*/*z* calcd for C_26_H_20_ClN_3_NaO_3_^+^ [M + Na]^+^ 480.1091, found 480.1090.

*3′-(3-Chlorophenyl)-1-methyl-5′-phenyl-2′,3′,3a′,6a′-tetrahydro-4′H-spiro[indoline-3,1′-pyrrolo[3,4-c]pyrrole]-2,4′,6′(5′H)-trione* (**4f**). White solid, 64.9 mg, 71% yield. 76:24 dr. ^1^H-NMR (400 MHz, CDCl_3_): δ 7.58 (s, 1H), 7.47 (t, *J* = 7.5 Hz, 2H), 7.41 (t, *J* = 7.5 Hz, 3H), 7.34 (dd, *J* = 14.0, 7.1 Hz, 3H), 7.24 (d, *J* = 7.6 Hz, 2H), 7.14 (t, *J* = 7.5 Hz, 1H), 6.90 (d, *J* = 7.8 Hz, 1H), 5.80 (d, *J* = 8.8 Hz, 1H), 4.03 (t, *J* = 8.4 Hz, 1H), 3.54 (d, *J* = 7.9 Hz, 1H), 3.25 (s, 3H); ^13^C-NMR (100 MHz, CDCl_3_): δ 178.6, 174.0, 173.3, 143.7, 140.3, 134.4, 130.3, 129.7, 129.2, 128.7, 128.4, 127.1, 126.7, 126.2, 125.9, 122.9, 108.4, 68.0, 60.1, 50.7, 49.4, 26.2; HRMS (ESI): *m*/*z* calcd for C_26_H_20_ClN_3_NaO_3_^+^ [M + Na]^+^ 480.1091, found 480.1109.

*3′-(2-Chlorophenyl)-1-methyl-5′-phenyl-2′,3′,3a′,6a′-tetrahydro-4′H-spiro[indoline-3,1′-pyrrolo[3,4-c]pyrrole]-2,4′,6′(5′H)-trione* (**4g**). White solid, 75.9 mg, 83% yield. 81:19 dr. ^1^H-NMR (400 MHz, CDCl_3_): δ 7.69 (dd, *J* = 5.6, 3.8 Hz, 1H), 7.48–7.44 (m, 2H), 7.42 (d, *J* = 7.6 Hz, 3H), 7.40–7.34 (m, 2H), 7.33 (d, *J* = 7.6 Hz, 1H), 7.20 (d, *J* = 7.5 Hz, 2H), 7.14 (t, *J* = 7.5 Hz, 1H), 6.91 (d, *J* = 7.8 Hz, 1H), 6.11 (d, *J* = 8.4 Hz, 1H), 4.31 (t, *J* = 8.1 Hz, 1H), 3.62 (d, *J* = 8.0 Hz, 1H), 3.26 (s, 3H); ^13^C-NMR (100 MHz, CDCl_3_): δ 178.6, 173.8, 173.5, 143.8, 136.0, 134.0, 130.3, 129.4, 129.1, 129.0, 128.5, 127.0, 126.8, 126.7, 126.2, 122.9, 108.5, 67.6, 57.6, 51.0, 47.0, 26.2; HRMS (ESI): *m*/*z* calcd for C_26_H_20_ClN_3_NaO_3_^+^ [M + Na]^+^ 480.1091, found 480.1116.

*3′-(3,4-Dichlorophenyl)-1-methyl-5′-phenyl-2′,3′,3a′,6a′-tetrahydro-4′H-spiro[indoline-3,1′-pyrrolo[3,4-c]pyrrole]-2,4′,6′(5′H)-trione* (**4h**). White solid, 83.5 mg, 85% yield. 86:14 dr. ^1^H-NMR (400 MHz, CDCl_3_): δ 7.64 (s, 1H), 7.46 (dd, *J* = 15.9, 7.9 Hz, 3H), 7.42–7.35 (m, 3H), 7.35–7.29 (m, 1H), 7.22 (d, *J* = 7.6 Hz, 2H), 7.13 (t, *J* = 7.5 Hz, 1H), 6.89 (d, *J* = 7.8 Hz, 1H), 5.76 (dd, *J* = 8.6, 3.2 Hz, 1H), 3.99 (t, *J* = 8.3 Hz, 1H), 3.53 (d, *J* = 7.9 Hz, 1H), 3.23 (s, 3H); ^13^C-NMR (100 MHz, CDCl_3_): δ 178.5, 173.9, 173.2, 143.7, 138.5, 132.1, 130.4, 129.3, 128.9, 128.8, 127.0, 126.2, 124.9, 122.9, 108.5, 67.9, 59.6, 50.7, 49.3, 26.2; HRMS (ESI): *m*/*z* calcd for C_26_H_20_Cl_2_N_3_O_3_^+^ [M + H]^+^ 492.0882, found 492.0905.

*3′-(4-Bromophenyl)-1-methyl-5′-phenyl-2′,3′,3a′,6a′-tetrahydro-4′H-spiro[indoline-3,1′-pyrrolo[3,4-c]pyrrole]-2,4′,6′(5′H)-trione* (**4i**). White solid, 81.2 mg, 81% yield. >95:5 dr. ^1^H-NMR (400 MHz, CDCl_3_): δ 7.47 (dd, *J* = 16.4, 8.1 Hz, 4H), 7.39 (dd, *J* = 12.5, 5.4 Hz, 4H), 7.31 (d, *J* = 7.3 Hz, 1H), 7.22 (d, *J* = 7.6 Hz, 2H), 7.12 (t, *J* = 7.5 Hz, 1H), 6.89 (d, *J* = 7.8 Hz, 1H), 5.77 (d, *J* = 7.4 Hz, 1H), 3.98 (t, *J* = 8.3 Hz, 1H), 3.55 (d, *J* = 7.9 Hz, 1H), 3.23 (s, 3H); ^13^C-NMR (100 MHz, CDCl_3_): δ 178.7, 173.9, 173.3, 143.7, 137.0, 131.5, 130.3, 129.2, 129.0, 128.6, 126.9, 126.1, 125.3, 122.9, 122.1, 108.4, 67.9, 60.2, 50.9, 49.3, 26.2; HRMS (ESI): *m*/*z* calcd for C_26_H_20_BrN_3_NaO_3_^+^ [M + Na]^+^ 524.0586, found 524.0567.

*3′-(3-Bromophenyl)-1-methyl-5′-phenyl-2′,3′,3a′,6a′-tetrahydro-4′H-spiro[indoline-3,1′-pyrrolo[3,4-c]pyrrole]-2,4′,6′(5′H)-trione* (**4j**).White solid, 65.1 mg, 65% yield. 67:33 dr. ^1^H-NMR (400 MHz, CDCl_3_): δ 7.72 (s, 1H), 7.46 (t, *J* = 7.7 Hz, 4H), 7.40 (t, *J* = 7.1 Hz, 2H), 7.34 (d, *J* = 7.4 Hz, 1H), 7.24 (dd, *J* = 7.5, 4.7 Hz, 3H), 7.13 (t, *J* = 7.5 Hz, 1H), 6.89 (d, *J* = 7.8 Hz, 1H), 5.78 (d, *J* = 8.9 Hz, 1H), 4.02 (t, *J* = 8.4 Hz, 1H), 3.53 (d, *J* = 7.9 Hz, 1H), 3.24 (s, 3H); ^13^C-NMR (100 MHz, CDCl_3_): δ 178.6, 173.9, 173.2, 143.7, 140.6, 131.4, 130.3, 130.0, 129.9, 129.2, 128.6, 127.1, 126.4, 126.2, 125.0, 122.9, 108.4, 68.0, 60.1, 50.7, 49.4, 26.2; HRMS (ESI): *m*/*z* calcd for C_26_H_20_BrN_3_NaO_3_^+^ [M + Na]^+^ 524.0586, found 524.0570.

*3′-(2-Bromophenyl)-1-methyl-5′-phenyl-2′,3′,3a′,6a′-tetrahydro-4′H-spiro[indoline-3,1′-pyrrolo[3,4-c]pyrrole]-2,4′,6′(5′H)-trione* (**4k**). White solid, 90.2 mg, 90% yield. 93:7 dr. ^1^H-NMR (400 MHz, CDCl_3_): δ 7.70–7.65 (m, 1H), 7.62 (d, *J* = 7.9 Hz, 1H), 7.41 (d, *J* = 7.7 Hz, 3H), 7.39–7.29 (m, 3H), 7.19 (d, *J* = 7.5 Hz, 3H), 7.13 (t, *J* = 7.5 Hz, 1H), 6.90 (d, *J* = 7.8 Hz, 1H), 6.07 (d, *J* = 8.3 Hz, 1H), 4.34 (t, *J* = 8.2 Hz, 1H), 3.60 (d, *J* = 8.0 Hz, 1H), 3.25 (s, 3H); ^13^C-NMR (100 MHz, CDCl_3_): δ 178.6, 173.8, 173.5, 143.8, 137.6, 132.7, 130.3, 129.4, 129.1, 128.5, 127.4, 127.2, 126.8, 126.1, 124.3, 122.9, 108.5, 67.7 , 59.9, 50.8, 46.9, 26.2; HRMS (ESI): *m*/*z* calcd for C_26_H_20_BrN_3_NaO_3_^+^ [M + Na]^+^ 524.0586, found 524.0561.

*1-Methyl-3′-(4-nitrophenyl)-5′-phenyl-2′,3′,3a′,6a′-tetrahydro-4′H-spiro[indoline-3,1′-pyrrolo[3,4-c]pyrrole]-2,4′,6′(5′H)-trione* (**4l**). White solid, 79.6 mg, 85% yield. >95:5 dr. ^1^H-NMR (400 MHz, CDCl_3_): δ 8.24 (d, *J* = 8.6 Hz, 2H), 7.72 (d, *J* = 8.6 Hz, 2H), 7.47 (dd, *J* = 12.9, 5.2 Hz, 2H), 7.41 (dd, *J* = 7.6, 2.2 Hz, 2H), 7.32 (d, *J* = 7.3 Hz, 1H), 7.23 (d, *J* = 7.6 Hz, 2H), 7.16 (t, *J* = 7.5 Hz, 1H), 6.92 (d, *J* = 7.8 Hz, 1H), 5.94 (d, *J* = 8.5 Hz, 1H), 4.06 (t, *J* = 8.3 Hz, 1H), 3.62 (d, *J* = 8.0 Hz, 1H), 3.26 (s, 3H); ^13^C-NMR (100 MHz, CDCl_3_): δ 178.5, 173.8, 173.1, 147.7, 145.6, 143.7, 131.4, 130.5, 129.3, 128.9, 128.1, 126.8, 126.1, 125.0, 123.6, 123.5, 123.1, 108.7, 68.0, 60.0, 50.7, 49.5, 26.3; HRMS (ESI): *m*/*z* calcd for C_26_H_20_N_4_NaO_5_^+^ [M + Na]^+^ 491.1331, found 491.1331.

*1-Methyl-3′-(3-nitrophenyl)-5′-phenyl-2′,3′,3a′,6a′-tetrahydro-4′H-spiro[indoline-3,1′-pyrrolo[3,4-c]pyrrole]-2,4′,6′(5′H)-trione* (**4m**). White solid; 77.7 mg, 83% yield; >95:5 dr; ^1^H-NMR (400 MHz, CDCl_3_): δ 8.43 (s, 1H), 8.17 (d, *J* = 7.9 Hz, 1H), 7.84 (d, *J* = 7.6 Hz, 1H), 7.55 (t, *J* = 7.9 Hz, 1H), 7.45 (d, *J* = 7.8 Hz, 2H), 7.40 (dd, *J* = 7.3, 3.6 Hz, 2H), 7.33 (d, *J* = 7.3 Hz, 1H), 7.23 (d, *J* = 7.7 Hz, 2H), 7.15 (t, *J* = 7.5 Hz, 1H), 6.90 (d, *J* = 7.7 Hz, 1H), 5.93 (d, *J* = 8.5 Hz, 1H), 4.04 (t, *J* = 8.2 Hz, 1H), 3.60 (d, *J* = 7.9 Hz, 1H), 3.25 (s, 3H); ^13^C-NMR (100 MHz, CDCl_3_): δ 178.6, 174.0, 173.2, 148.4 , 143.7, 140.4, 133.9, 130.5, 129.4, 129.3, 129.2, 128.9, 126.9, 126.4, 126.3, 123.3, 123.1, 122.0, 108.6, 67.9, 59.9, 50.7, 49.4, 26.2; HRMS (ESI): *m*/*z* calcd for C_26_H_20_N_4_NaO_5_^+^ [M + Na]^+^ 491.1331, found 491.1332.

*1-Methyl-3′-(2-nitrophenyl)-5′-phenyl-2′,3′,3a′,6a′-tetrahydro-4′H-spiro[indoline-3,1′-pyrrolo[3,4-c]pyrrole]-2,4′,6′(5′H)-trione* (**4n**). White solid; 80.5 mg, 86% yield; 94:6 dr; ^1^H-NMR (400 MHz, CDCl_3_): δ 8.13 (d, *J* = 8.1 Hz, 1H), 8.06 (d, *J* = 7.8 Hz, 1H), 7.62 (t, *J* = 7.6 Hz, 1H), 7.44–7.38 (m, 3H), 7.38–7.32 (m, 3H), 7.17 (d, *J* = 7.6 Hz, 2H), 7.13 (t, *J* = 7.5 Hz, 1H), 6.89 (d, *J* = 7.8 Hz, 1H), 6.23 (dd, *J* = 8.2, 2.8 Hz, 1H), 4.51 (t, *J* = 8.2 Hz, 1H), 3.60 (d, *J* = 8.1 Hz, 1H), 3.22 (s, 3H); ^13^C-NMR (100 MHz, CDCl_3_): δ 178.5, 174.2, 173.4, 148.7, 143.8, 134.5, 133.4, 130.4, 129.2, 128.8, 128.7, 128.1, 126.7, 126.1, 125.3, 122.8, 108.6, 67.7, 56.9, 50.7, 48.5, 26.2; HRMS (ESI): *m*/*z* calcd for C_26_H_20_N_4_NaO_5_^+^ [M + Na]^+^ 491.1331, found 491.1350.

*1-Methyl-5′-phenyl-3′-(p-tolyl)-2′,3′,3a′,6a′-tetrahydro-4′H-spiro[indoline-3,1′-pyrrolo[3,4-c]pyrrole]-2,4′,6′(5′H)-trione* (**4o**). White solid; 68.2 mg, 78% yield; 82:18 dr; ^1^H-NMR (400 MHz, CDCl_3_): δ 7.43 (dd, *J* = 14.0, 6.1 Hz, 3H), 7.38 (d, *J* = 3.3 Hz, 1H), 7.35 (d, *J* = 3.5 Hz, 4H), 7.23 (d, *J* = 7.7 Hz, 2H), 7.12 (dd, *J* = 8.7, 5.2 Hz, 2H), 6.88 (d, *J* = 8.0 Hz, 1H), 5.76 (d, *J* = 8.9 Hz, 1H), 4.00 (t, *J* = 8.4 Hz, 1H), 3.50 (d, *J* = 7.9 Hz, 1H), 3.23 (s, 3H), 2.36 (s, 3H); ^13^C-NMR (100 MHz, CDCl_3_): δ 178.8, 174.2, 173.5, 143.8, 138.0, 137.9, 130.2, 129.1, 128.5, 128.3, 128.0, 127.1, 126.2, 124.5, 122.8, 108.4, 68.1 , 60.7, 50.9, 49.5, 26.2, 21.6; HRMS (ESI): *m*/*z* calcd for C_27_H_23_N_3_NaO_3_^+^ [M + Na]^+^ 460.1637, found 460.1658.

*1-Methyl-5′-phenyl-3′-(m-tolyl)-2′,3′,3a′,6a′-tetrahydro-4′H-spiro[indoline-3,1′-pyrrolo[3,4-c]pyrrole]-2,4′,6′(5′H)-trione* (**4p**). White solid; 62.1 mg, 71% yield; 75:25 dr; ^1^H-NMR (400 MHz, CDCl_3_): δ 7.46–7.40 (m, 4H), 7.37 (d, *J* = 7.4 Hz, 2H), 7.34 (d, *J* = 7.3 Hz, 1H), 7.24 (d, *J* = 7.5 Hz, 2H), 7.18 (d, *J* = 7.8 Hz, 2H), 7.12 (t, *J* = 7.5 Hz, 1H), 6.88 (d, *J* = 7.7 Hz, 1H), 5.78 (d, *J* = 8.7 Hz, 1H), 3.98 (t, *J* = 8.4 Hz, 1H), 3.52 (d, *J* = 7.9 Hz, 1H), 3.23 (s, 3H), 2.35 (s, 3H); ^13^C-NMR (100 MHz, CDCl_3_): δ 178.8, 174.2, 173.6, 143.7, 137.9, 134.9, 130.2, 129.2, 129.1, 128.5, 127.2, 127.0, 126.7, 126.2, 125.5, 122.8, 108.4, 67.9, 60.6, 51.0, 49.5, 26.2, 21.3; HRMS (ESI): *m*/*z* calcd for C_27_H_24_N_3_O_3_^+^ [M + H]^+^ 438.1818, found 438.1825.

*1-Methyl-5′-phenyl-3′-(o-tolyl)-2′,3′,3a′,6a′-tetrahydro-4′H-spiro[indoline-3,1′-pyrrolo[3,4-c]pyrrole]-2,4′,6′(5′H)-trione* (**4q**). White solid; 69.9 mg, 80% yield; 77:23 dr; ^1^H-NMR (400 MHz, CDCl_3_): δ 7.75–7.68 (m, 1H), 7.40 (t, *J* = 7.7 Hz, 4H), 7.36–7.30 (m, 1H), 7.21 (d, *J* = 7.8 Hz, 3H), 7.13 (t, *J* = 8.4 Hz, 3H), 6.90 (d, *J* = 7.7 Hz, 1H), 5.94 (d, *J* = 9.0 Hz, 1H), 4.13 (t, *J* = 8.4 Hz, 1H), 3.53 (d, *J* = 7.9 Hz, 1H), 3.25 (s, 3H), 2.54 (s, 3H); ^13^C-NMR (100 MHz, CDCl_3_): δ 178.8, 173.9, 173.5, 143.8, 136.6, 130.3, 130.2, 129.1, 128.5, 127.8, 127.3, 126.2, 125.8, 125.1, 122.8, 108.4, 67.8, 57.1, 50.9, 47.2, 26.2, 19.5; HRMS (ESI): *m*/*z* calcd for C_27_H_23_N_3_NaO_3_^+^ [M + Na]^+^ 460.1637, found 460.1646.

*3′-(4-Methoxyphenyl)-1-methyl-5′-phenyl-2′,3′,3a′,6a′-tetrahydro-4′H-spiro[indoline-3,1′-pyrrolo[3,4-c]pyrrole]-2,4′,6′(5′H)-trione* (**4r**). White solid; 76.1 mg, 84% yield; 83:17 dr; ^1^H-NMR (400 MHz, CDCl_3_): δ 7.46 (t, *J* = 7.7 Hz, 4H), 7.41–7.35 (m, 3H), 7.28–7.24 (m, 2H), 7.13 (t, *J* = 7.6 Hz, 1H), 6.94–6.88 (m, 3H), 5.78 (d, *J* = 8.8 Hz, 1H), 3.99 (t, *J* = 8.5 Hz, 1H), 3.82 (s, 3H), 3.53 (d, *J* = 7.9 Hz, 1H), 3.25 (s, 3H); ^13^C-NMR (100MHz, CDCl_3_): δ 178.8, 174.2, 173.5, 143.8, 138.0, 137.9, 129.1, 128.5, 128.3, 128.0, 127.1, 126.2, 124.5, 122.8, 108.4, 68.1, 60.7, 50.9, 49.5, 26.2, 21.6; HRMS (ESI): *m*/*z* calcd for C_27_H_23_N_3_NaO_4_^+^ [M + Na]^+^ 476.1586, found 476.1597.

*3′-(3-Methoxyphenyl)-1-methyl-5′-phenyl-2′,3′,3a′,6a′-tetrahydro-4′H-spiro[indoline-3,1′-pyrrolo[3,4-c]pyrrole]-2,4′,6′(5′H)-trione* (**4s**). White solid; 68.9 mg, 76% yield; 80:20 dr; ^1^H-NMR (400 MHz, CDCl_3_): δ 7.48–7.42 (m, 2H), 7.41–7.30 (m, 4H), 7.27–7.22 (m, 2H), 7.14 (dd, *J* = 12.9, 5.2 Hz, 3H), 6.89 (d, *J* = 7.9 Hz, 1H), 6.86 (dd, *J* = 7.9, 2.2 Hz, 1H), 5.79 (d, *J* = 8.9 Hz, 1H), 4.02 (t, *J* = 8.4 Hz, 1H), 3.81 (s, 3H), 3.53 (d, *J* = 7.9 Hz, 1H), 3.25 (s, 3H); ^13^C-NMR (100 MHz, CDCl_3_): δ 178.7, 174.0, 173.5, 143.7, 139.8, 130.2, 129.4, 129.1, 128.5, 127.1, 126.7, 126.2, 125.3, 122.8, 119.8, 108.4, 68.0, 60.6, 55.2, 50.9, 49.4, 26.2; HRMS (ESI): *m*/*z* calcd for C_27_H_23_N_3_NaO_4_^+^ [M + Na]^+^ 476.1586, found 476.1605.

*1-Methyl-3′-(naphthalen-1-yl)-5′-phenyl-2′,3′,3a′,6a′-tetrahydro-4′H-spiro[indoline-3,1′-pyrrolo[3,4-c]pyrrole]-2,4′,6′(5′H)-trione* (**4t**). White solid; 88.0 mg, 93% yield; 94:6 dr; ^1^H-NMR (400 MHz, CDCl_3_): δ 8.26 (d, *J* = 8.4 Hz, 1H), 7.90 (d, *J* = 8.4 Hz, 2H), 7.83 (d, *J* = 8.1 Hz, 1H), 7.64 (t, *J* = 7.5 Hz, 1H), 7.54 (t, *J* = 7.5 Hz, 1H), 7.48 (d, *J* = 7.8 Hz, 1H), 7.41 (dd, *J* = 14.6, 7.3 Hz, 5H), 7.18–7.12 (m, 3H), 6.93 (d, *J* = 7.9 Hz, 1H), 6.57 (d, *J* = 8.5 Hz, 1H), 4.32 (t, *J* = 8.2 Hz, 1H), 3.64 (d, *J* = 7.8 Hz, 1H), 3.28 (s, 3H); ^13^C-NMR (100 MHz, CDCl_3_): δ 178.7, 173.6, 173.5, 143.8,130.3, 129.1, 129.0, 128.6, 128.5, 127.1, 126.5, 126.2, 125.8, 125.2, 122.9, 108.5, 67.7, 56.5, 51.1, 48.4, 26.2; HRMS (ESI): *m*/*z* calcd for C_30_H_23_N_3_NaO_3_^+^ [M + Na]^+^ 496.1637, found 496.1651.

*3′-(Furan-2-yl)-1-methyl-5′-phenyl-2′,3′,3a′,6a′-tetrahydro-4′H-spiro[indoline-3,1′-pyrrolo[3,4-c]pyrrole]-2,4′,6′(5′H)-trione* (**4u**). White solid; 57.8 mg, 70% yield; 94:6 dr; ^1^H-NMR (400 MHz, CDCl_3_): δ 7.48 (d, *J* = 8.0 Hz, 3H), 7.39 (dd, *J* = 16.4, 7.7 Hz, 3H), 7.32 (d, *J* = 7.5 Hz, 2H), 7.10 (t, *J* = 7.6 Hz, 1H), 6.88 (d, *J* = 7.8 Hz, 1H), 6.43 (d, *J* = 3.1 Hz, 1H), 6.40–6.36 (m, 1H), 5.78 (d, *J* = 8.8 Hz, 1H), 4.02 (t, *J* = 8.4 Hz, 1H), 3.51 (d, *J* = 8.0 Hz, 1H), 3.21 (s, 3H); ^13^C-NMR (100 MHz, CDCl_3_): δ 178.3, 174.4, 173.5, 151.2, 143.8, 142.7, 131.9, 130.3, 129.2, 128.7, 126.9, 126.3, 124.8, 122.8, 110.4, 108.4, 108.1, 67.9, 55.8, 50.7, 48.6, 26.2; HRMS (ESI): *m*/*z* calcd for C_24_H_19_N_3_NaO_4_^+^ [M + Na]^+^ 436.1273, found 436.1290.

*1-Methyl-5′-phenyl-3′-(thiophen-2-yl)-2′,3′,3a′,6a′-tetrahydro-4′H-spiro[indoline-3,1′-pyrrolo[3,4-c]pyrrole]-2,4′,6′(5′H)-trione* (**4v**). White solid; 67.8 mg, 79% yield; 93:7 dr; ^1^H-NMR (400 MHz, CDCl_3_): δ 7.47 (t, *J* = 7.6 Hz, 2H), 7.39 (dd, *J* = 7.5, 5.4 Hz, 2H), 7.34 (d, *J* = 7.6 Hz, 1H), 7.29 (s, 1H), 7.28 (d, *J* = 2.1 Hz, 2H), 7.22 (d, *J* = 3.2 Hz, 1H), 7.13 (t, *J* = 7.5 Hz, 1H), 7.08-7.04 (m, 1H), 6.89 (d, *J* = 7.7 Hz, 1H), 6.11 (d, *J* = 9.1 Hz, 1H), 4.02 (t, *J* = 8.5 Hz, 1H), 3.49 (d, *J* = 1.7 Hz, 1H), 3.24 (s, 3H); ^13^C-NMR (100 MHz, CDCl_3_): δ 178.5, 173.8, 173.4, 143.7, 142.7, 131.8, 130.3, 129.2, 128.6, 127.3, 127.2, 126.7, 126.4, 125.5, 125.0, 124.8, 122.8, 108.4, 67.8, 57.1, 50.5, 49.3, 26.2; HRMS (ESI): *m*/*z* calcd for C_24_H_19_N_3_NaO_3_S^+^ [M + Na]^+^ 452.1045, found 452.1066.

*3′-Benzyl-1-methyl-5′-phenyl-2′,3′,3a′,6a′-tetrahydro-4′H-spiro[indoline-3,1′-pyrrolo[3,4-c]pyrrole]-2,4′,6′(5′H)-trione* (**4w**). White solid; 66.5 mg, 76% yield; 77:23 dr; ^1^H-NMR (400 MHz, CDCl_3_): δ 7.56 (t, *J* = 7.6 Hz, 2H), 7.46 (dd, *J* = 12.7, 7.4 Hz, 4H), 7.31 (t, *J* = 7.2 Hz, 5H), 7.23 (dd, *J* = 13.3, 6.6 Hz, 2H), 6.82 (d, *J* = 7.7 Hz, 1H), 4.87–4.78 (m, 1H), 3.77 (t, *J* = 7.8 Hz, 1H), 3.57 (d, *J* = 8.0 Hz, 1H), 3.29–3.23 (m, 1H), 3.15 (s, 3H), 2.80–2.71 (m, 1H); ^13^C-NMR (100 MHz, CDCl_3_): δ 178.5, 175.4, 173.9, 143.6, 139.4, 131.9, 130.1, 129.4, 129.1, 128.8, 128.7, 128.4, 126.5, 126.3, 122.8, 108.4, 67.7, 58.8, 51.7, 47.6, 38.2, 26.1; HRMS (ESI): *m*/*z* calcd for C_27_H_23_N_3_NaO_3_^+^ [M + Na]^+^ 460.1637, found 460.1647.

*3′-(tert-Butyl)-1-methyl-5′-phenyl-2′,3′,3a′,6a′-tetrahydro-4′H-spiro[indoline-3,1′-pyrrolo[3,4-c]pyrrole]-2,4′,6′(5′H)-trione* (**4x**). White solid; 58.1 mg; 72% yield; 72:28 dr; ^1^H-NMR (400 MHz, CDCl_3_): δ 7.50 (d, *J* = 7.7 Hz, 3H), 7.37–7.30 (m, 4H), 6.87 (d, *J* = 7.9 Hz, 2H), 4.38 (d, *J* = 7.9 Hz, 1H), 3.75 (t, *J* = 8.0 Hz, 1H), 3.58 (d, *J* = 8.1 Hz, 1H), 3.22 (s, 3H), 1.19 (s, 9H); ^13^C-NMR (100 MHz, CDCl_3_): δ 178.8, 176.3, 173.5, 143.6, 130.0, 129.3, 128.8, 126.5, 126.2, 122.8, 108.4, 68.4, 67.3, 52.2, 47.0, 33.2, 29.7, 26.2; HRMS (ESI): *m*/*z* calcd for C_24_H_25_N_3_NaO_3_^+^ [M + Na]^+^ 426.1794, found 426.1808.

*5-Methoxy-1-methyl-3′,5′-diphenyl-2′,3′,3a′,6a′-tetrahydro-4′H-spiro[indoline-3,1′-pyrrolo[3,4-c]pyrrole]-2,4′,6′(5′H)-trione* (**5a**). White solid; 74.3 mg; 82% yield; 83:17 dr; ^1^H-NMR (400 MHz, CDCl_3_): δ 7.54 (d, *J* = 7.5 Hz, 2H), 7.42 (dd, *J* = 14.5, 6.9 Hz, 3H), 7.39–7.32 (m, 3H), 7.23 (d, *J* = 7.8 Hz, 2H), 6.94 (s, 1H), 6.89 (d, *J* = 8.5 Hz, 1H), 6.78 (d, *J* = 8.4 Hz, 1H), 5.80 (d, *J* = 8.6 Hz, 1H), 3.99 (t, *J* = 8.3 Hz, 1H), 3.76 (s, 3H), 3.55 (d, *J* = 7.9 Hz, 1H), 3.21 (s, 3H); ^13^C-NMR (100 MHz, CDCl_3_): δ 178.6, 174.1, 173.4, 155.9, 138.0, 137.1, 129.1, 128.5, 128.3, 128.2, 127.3, 127.0, 126.2, 114.5, 114.3, 108.7, 68.2, 60.9, 55.8, 51.1, 49.6, 26.2; HRMS (ESI): *m*/*z* calcd for C_27_H_23_N_3_NaO_4_^+^ [M + Na]^+^ 476.1586, found 476.1605.

*5-Methoxy-1-methyl-3′-(4-nitrophenyl)-5′-phenyl-2′,3′,3a′,6a′-tetrahydro-4′H-spiro[indoline-3,1′-pyrrolo[3,4-c]pyrrole]-2,4′,6′(5′H)-trione* (**5b**). White solid; 79.7 mg; 80% yield; 82:18 dr; ^1^H-NMR (400 MHz, CDCl_3_): δ 8.26 (dd, *J* = 8.4, 4.9 Hz, 3H), 7.88 (d, *J* = 8.6 Hz, 2H), 7.48 (d, *J* = 7.7 Hz, 2H), 7.42 (d, *J* = 7.2 Hz, 1H), 6.91 (d, *J* = 8.4 Hz, 3H), 6.79 (d, *J* = 8.5 Hz, 1H), 5.63 (d, *J* = 6.8 Hz, 1H), 3.95 (t, *J* = 10.8 Hz, 1H), 3.86 (s, 3H), 3.57 (dd, *J* = 10.0, 7.0 Hz, 1H), 3.17 (s, 3H); ^13^C-NMR (100 MHz, CDCl_3_): δ 177.29 (s), 175.51 (s), 173.89 (s), 156.90 (s), 148.76 (s), 137.30 (s), 131.74 (s), 130.47 (s), 129.04 (d, *J* = 31.4 Hz), 128.76 (s), 127.66 (s), 126.87 (s), 126.41 (s), 123.94 (s), 114.48 (s), 111.45 (s), 109.36 (s), 69.20 (s), 61.17 (s), 55.97 (s), 53.48 (s), 52.95 (s), 26.26 (s). HRMS (ESI): *m*/*z* calcd for C_27_H_22_N_4_NaO_6_^+^ [M + Na]^+^ 521.1437, found 521.1437.

*5-Chloro-1-methyl-3′,5′-diphenyl-2′,3′,3a′,6a′-tetrahydro-4′H-spiro[indoline-3,1′-pyrrolo[3,4-c]pyrrole]-2,4′,6′(5′H)-trione* (**5c**). White solid; 77.7 mg; 85% yield; 75:25 dr; ^1^H-NMR (400 MHz, CDCl_3_): δ 7.53 (d, *J* = 7.2 Hz, 3H), 7.39-7.31 (m, 7H), 7.21 (d, *J* = 7.3 Hz, 3H), 5.76 (d, *J* = 8.8 Hz, 1H), 4.02 (t, *J* = 8.3 Hz, 1H), 3.53 (d, *J* = 7.9 Hz, 1H), 3.22 (s, 3H); ^13^C-NMR (100 MHz, CDCl_3_): δ 178.4, 174.0, 173.3, 142.3, 137.7, 130.1, 129.2, 128.8, 128.7, 128.6, 128.4, 128.3, 127.4, 127.3, 126.1, 109.4, 67.9, 60.9, 51.0, 49.4, 26.3; HRMS (ESI): *m*/*z* calcd for C2_6_H_20_ClN_3_NaO_3_^+^ [M + Na]^+^ 480.1091, found 480.1103.

*5-Chloro-1-methyl-3′-(4-nitrophenyl)-5′-phenyl-2′,3′,3a′,6a′-tetrahydro-4′H-spiro[indoline-3,1′-pyrrolo[3,4-c]pyrrole]-2,4′,6′(5′H)-trione* (**5d**). White solid; 82.3 mg; 82% yield; 78:22 dr; ^1^H-NMR (400 MHz, CDCl_3_): δ 8.22 (d, *J* = 7.9 Hz, 2H), 7.84 (d, *J* = 7.7 Hz, 2H), 7.52–7.43 (m, 4H), 7.41 (d, *J* = 6.3 Hz, 2H), 6.78 (t, *J* = 8.4 Hz, 2H), 5.56 (d, *J* = 6.1 Hz, 1H), 3.85 (d, *J* = 9.9 Hz, 1H), 3.54 (t, *J* = 9.8 Hz, 1H), 3.14 (s, 3H); ^13^C-NMR (100 MHz, CDCl_3_): δ 177.2, 175.5, 173.9, 148.5, 147.6, 142.5, 130.3, 130.2, 129.3, 129.0, 127.7, 127.2, 126.8, 126.7, 124.6, 124.0, 110.0, 68.8, 61.2, 53.4, 52.8, 26.3; HRMS (ESI): *m*/*z* calcd for C_26_H_19_ClN_4_NaO_5_^+^ [M + Na]^+^ 525.0942, found 525.0962.

*3′,5′-Diphenyl-2′,3′,3a′,6a′-tetrahydro-4′H-spiro[indoline-3,1′-pyrrolo[3,4-c]pyrrole]-2,4′,6′(5′H)-trione* (**5e**). White solid; 55.6 mg; 68% yield; 69:31 dr; ^1^H-NMR (400 MHz, DMSO-*d*_6_): δ 10.46 (s, 1H), 7.44 (d, *J* = 26.8 Hz, 6H), 7.24 (d, *J* = 36.5 Hz, 8H), 5.53 (s, 1H), 4.27 (s, 1H), 3.88 (s, 1H); ^13^C-NMR (100 MHz, DMSO-*d*_6_): δ 181.3, 174.9, 174.3, 142.8, 140.0, 129.6, 129.4, 128.2, 128.0, 127.7, 127.4,127.3, 121.5, 109.8, 68.1, 60.6, 51.8, 50.4; HRMS (ESI): *m*/*z* calcd for C_25_H_19_N_3_NaO_3_^+^ [M + Na]^+^ 432.1324, found 432.1341.

*1-Benzyl-3′,5′-diphenyl-2′,3′,3a′,6a′-tetrahydro-4′H-spiro[indoline-3,1′-pyrrolo[3,4-c]pyrrole]-2,4′,6′(5′H)-trione* (**5f**). White solid; 79.9 mg; 80% yield; 83:17 dr; ^1^H-NMR (400 MHz, DMSO-*d*_6_): δ 7.50 (s, 5H), 7.42–7.18 (m, 12H), 7.00 (s, 1H), 6.85 (s, 1H), 5.60 (s, 1H), 4.90 (s, 2H), 3.94 (s, 1H), 3.57 (s, 1H); ^13^C-NMR (100 MHz, DMSO-*d*_6_): δ 179.4, 174.8, 174.1, 143.2, 139.9, 136.7, 133.3, 129.7, 129.4, 129.1, 129.0, 128.8, 128.2, 128.0, 127.7, 127.4, 127.2, 122.3, 109.4, 68.0, 60.8, 52.1, 50.4, 42.9; HRMS (ESI): *m*/*z* calcd for C_32_H_25_N_3_NaO_3_^+^ [M + Na]^+^ 522.1794, found 522.1799.

*1-Benzyl-3′-(4-nitrophenyl)-5′-phenyl-2′,3′,3a′,6a′-tetrahydro-4′H-spiro[indoline-3,1′-pyrrolo[3,4-c]pyrrole]-2,4′,6′(5′H)-trione* (**5g**). White solid; 88.2 mg; 81% yield; 82:18 dr; ^1^H-NMR (400 MHz, DMSO-*d*_6_): δ 8.22 (d, *J* = 7.9 Hz, 2H), 7.85 (d, *J* = 7.9 Hz, 2H), 7.48 (d, *J* = 6.8 Hz, 2H), 7.41 (s, 3H), 7.37–7.32 (m, 2H), 7.30–7.21 (m, 5H), 7.04 (t, *J* = 6.8 Hz, 1H), 6.88 (d, *J* = 7.2 Hz, 1H), 5.05–4.85 (m, 2H), 4.70 (s, 1H), 4.13 (t, *J* = 7.7 Hz, 1H), 3.66 (d, *J* = 7.4 Hz, 1H); ^13^C-NMR (100 MHz, DMSO-*d*_6_): δ 179.3, 174.9, 174.0, 148.3, 147.2, 143.2, 136.6, 129.8, 129.5, 129.2, 129.1, 128.9, 127.9, 127.8, 127.4, 127.1, 127.0, 123.4, 122.5, 109.6, 68.0, 60.1, 52.2, 50.5, 43.0; HRMS (ESI): *m*/*z* calcd for C_32_H_24_N_4_NaO_5_^+^ [M + Na]^+^ 567.1644, found 567.1646.

*5′-(4-Chlorophenyl)-1-methyl-3′-phenyl-2′,3′,3a′,6a′-tetrahydro-4′H-spiro[indoline-3,1′-pyrrolo[3,4-c]pyrrole]-2,4′,6′(5′H)-trione* (**5h**). White solid; 60.3 mg; 66% yield; 67:33 dr; ^1^H-NMR (400 MHz, DMSO-*d*_6_): δ 7.55 (d, *J* = 7.9 Hz, 3H), 7.47 (d, *J* = 7.1 Hz, 3H), 7.28 (d, *J* = 7.1 Hz, 2H), 7.23 (d, *J* = 7.9 Hz, 3H), 7.01 (d, *J* = 8.2 Hz, 2H), 5.54 (d, *J* = 6.1 Hz, 1H), 3.90 (t, *J* = 8.1 Hz, 1H), 3.50 (d, *J* = 7.6 Hz, 1H), 3.14 (s, 3H); ^13^C-NMR (100 MHz, DMSO-*d*_6_): δ 179.1, 174.7, 174.0, 144.4, 139.9, 133.2, 131.6, 129.7, 129.5, 129.1, 128.2, 127.9, 127.7, 127.1, 127.0, 122.2, 108.7, 67.9, 60.7, 51.8, 50.5, 26.3; HRMS (ESI): *m*/*z* calcd for C_26_H_20_ClN_3_NaO_3_^+^ [M + Na]^+^ 480.1091, found 480.1110.

*5′-(4-Chlorophenyl)-1-methyl-3′-(4-nitrophenyl)-2′,3′,3a′,6a′-tetrahydro-4′H-spiro[indoline-3,1′-pyrrolo[3,4-c]pyrrole]-2,4′,6′(5′H)-trione* (**5i**). White solid; 81.3 mg; 81% yield; 82:18 dr; ^1^H-NMR (400 MHz, CDCl_3_): δ 8.22 (d, *J* = 8.6 Hz, 2H), 7.68 (d, *J* = 8.6 Hz, 2H), 7.42 (t, *J* = 3.3 Hz, 2H), 7.28 (dd, *J* = 14.0, 7.8 Hz, 2H), 7.15 (dd, *J* = 16.8, 8.2 Hz, 3H), 6.91 (d, *J* = 7.8 Hz, 1H), 5.91 (d, *J* = 8.5 Hz, 1H), 4.03 (t, *J* = 8.2 Hz, 1H), 3.59 (d, *J* = 8.0 Hz, 1H), 3.24 (s, 3H); ^13^C-NMR (100 MHz, CDCl_3_): δ 178.5, 173.6, 172.9, 147.7, 145.5, 143.7, 134.5, 130.6, 129.9, 129.5, 129.4, 128.1, 128.0, 127.2, 126.6, 125.0, 123.6, 123.0, 108.7, 68.0, 60.0, 50.8, 49.5, 26.3; HRMS (ESI): *m*/*z* calcd for C_26_H_19_ClN_4_NaO_5_^+^ [M + Na]^+^ 525.0942, found 525.0964.

*5′-(3-Chlorophenyl)-1-methyl-3′-(4-nitrophenyl)-2′,3′,3a′,6a′-tetrahydro-4′H-spiro[indoline-3,1′-pyrrolo[3,4-c]pyrrole]-2,4′,6′(5′H)-trione* (**5j**). White solid; 80.3 mg; 80% yield; 81:19 dr; ^1^H-NMR (400 MHz, CDCl_3_): δ 8.24 (d, *J* = 8.7 Hz, 2H), 7.71 (d, *J* = 8.7 Hz, 2H), 7.46–7.35 (m, 3H), 7.28 (dd, *J* = 12.9, 4.8 Hz, 2H), 7.21–7.11 (m, 2H), 6.93 (d, *J* = 7.8 Hz, 1H), 5.94 (d, *J* = 8.5 Hz, 1H), 4.06 (t, *J* = 8.2 Hz, 1H), 3.61 (d, *J* = 8.0 Hz, 1H), 3.26 (s, 3H); ^13^C-NMR (100 MHz, CDCl_3_): δ 178.4, 173.5, 172.7, 147.7, 145.4, 143.7, 134.8, 130.6, 130.3, 129.0, 128.0, 126.7, 126.3, 124.9, 124.2, 123.7, 123.1, 108.7, 68.0, 60.0, 50.7, 49.5, 26.3; HRMS (ESI): *m*/*z* calcd for C_26_H_19_ClN_4_NaO_5_^+^ [M + Na]^+^ 525.0942, found 525.0965.

*5′-(4-Methoxyphenyl)-1-methyl-3′-(4-nitrophenyl)-2′,3′,3a′,6a′-tetrahydro-4′H-spiro[indoline-3,1′-pyrrolo[3,4-c]pyrrole]-2,4′,6′(5′H)-trione* (**5k**). White solid; 79.7 mg; 80% yield; 78:22 dr; ^1^H-NMR (400 MHz, DMSO-*d*_6_): δ 8.29 (s, 2H), 7.87 (s, 2H), 7.10 (d, *J* = 34.9 Hz, 8H), 5.26 (s, 1H), 4.48 (s, 1H), 3.99 (s, 1H), 3.78 (s, 3H), 3.09 (s, 3H); ^13^C-NMR (100 MHz, DMSO-*d*_6_): δ 177.9, 177.1, 175.3, 159.5, 150.8, 147.3, 144.4, 130.1, 129.8, 128.7, 128.6, 128.3, 125.2, 124.6, 124.3,124.1, 123.4, 114.7, 109.3, 100.0, 69.0, 60.9, 55.9, 53.6, 26.3; HRMS (ESI): *m*/*z* calcd for C_27_H_22_N_4_NaO_6_^+^ [M + Na]^+^ 521.1437, found 521.1450.

*5′-(3-Methoxyphenyl)-1-methyl-3′-(4-nitrophenyl)-2′,3′,3a′,6a′-tetrahydro-4′H-spiro[indoline-3,1′-pyrrolo[3,4-c]pyrrole]-2,4′,6′(5′H)-trione* (**5l**). White solid; 84.7 mg; 85% yield; 84:16 dr; ^1^H-NMR (400 MHz, CDCl_3_): δ 8.21 (d, *J* = 8.6 Hz, 2H), 7.70 (d, *J* = 8.6 Hz, 2H), 7.36 (ddd, *J* = 20.8, 14.1, 7.5 Hz, 4H), 7.14 (t, *J* = 7.5 Hz, 1H), 6.91 (dd, *J* = 11.2, 4.7 Hz, 2H), 6.79 (d, *J* = 7.9 Hz, 1H), 5.90 (d, *J* = 8.5 Hz, 1H), 4.03 (t, *J* = 8.2 Hz, 1H), 3.77 (s, 3H), 3.58 (d, *J* = 7.9 Hz, 1H), 3.23 (s, 3H); ^13^C-NMR (100 MHz, CDCl_3_): δ 178.6, 173.8, 173.1, 147.7, 145.7, 143.7, 132.5, 130.5, 130.0, 129.9, 128.1, 126.8, 125.1, 123.6, 123.0, 118.3, 114.4, 112.2, 108.6, 68.0, 60.0, 55.4, 50.8, 49.5, 26.2; HRMS (ESI): *m*/*z* calcd for C_27_H_22_N_4_NaO_6_^+^ [M + Na]^+^ 521.1437, found 521.1461.

*1-Methyl-3′-(4-nitrophenyl)-5′-(p-tolyl)-2′,3′,3a′,6a′-tetrahydro-4′H-spiro[indoline-3,1′-pyrrolo[3,4-c]pyrrole]-2,4′,6′(5′H)-trione* (**5m**). White solid; 78.1 mg; 81% yield; 82:18 dr; ^1^H-NMR (400 MHz, CDCl_3_): δ 8.21 (d, *J* = 8.6 Hz, 2H), 7.69(d, *J* = 8.6 Hz, 2H), 7.40(t, *J* = 7.5 Hz, 1H), 7.30 (d, *J* = 7.3 Hz, 1H), 7.28–7.21 (m, 2H), 7.13 (t, *J* = 7.5 Hz, 1H), 7.08 (d, *J* = 8.2 Hz, 2H), 6.89 (d, *J* = 7.8 Hz, 1H), 5.90 (d, *J* = 8.5 Hz, 1H), 4.02 (t, *J* = 8.2 Hz, 1H), 3.57 (d, *J* = 7.9 Hz, 1H), 3.23 (s, 3H), 2.36 (s, 3H); ^13^C-NMR (100 MHz, CDCl_3_): δ 178.6, 173.9, 173.2, 147.7, 145.7, 143.7, 138.9, 130.4, 129.9, 128.9, 128.1, 126.8, 125.9, 125.1, 123.6, 123.0, 108.6, 68.0, 60.0, 50.7, 49.5, 26.2, 21.2; HRMS (ESI): *m*/*z* calcd for C_27_H_22_N_4_NaO_5_^+^ [M + Na]^+^ 505.1488, found 505.1510.

## 4. Conclusions

In summary, we have developed a simple and efficient strategy for diastereoselective construction of structurally diverse succinimide-fused spiro[pyrrolidine-2,3′-oxindoles] by a one-pot three-component 1,3-dipolar cycloaddition reaction ofazomethine ylides generated in situ from 3-amino oxindolesand aldehydes with maleimides. A series of succinimide-fused spiro[pyrrolidine-2,3′-oxindole] compounds have been obtained in good to high yields (up to 93%) with moderate to excellent diastereoselectivities (up to >95:5). The relative stereochemistry of products has been assigned by X-ray diffraction analysis. Further biological applications of 3-aminooxindoles are currently underway.

## Figures and Tables

**Table 1:** Optimization of reaction conditions ^a^.

Entry	Base	Solvent	T (°C)	Time (h)	Yield ^b^ (%)	dr ^c^
1	NaHCO_3_	CHCl_3_	30	16	60	74:26
2	K_2_CO_3_	CHCl_3_	30	16	62	73:27
3	KF/Al_2_O_3_	CHCl_3_	30	16	58	71:29
4	NaOH	CHCl_3_	30	16	trace	-
5	DIPEA	CHCl_3_	30	16	65	81:19
6	TEA	CHCl_3_	30	16	68	83:17
7	TEA	CHCl_3_	30	24	74	84:16
8	TEA	CH_2_Cl_2_	30	24	60	88:12
9	TEA	DCE	30	24	61	83:17
10	TEA	THF	30	24	62	69:31
11	TEA	dioxane	30	24	41	80:20
12	TEA	Et_2_O	30	24	55	69:31
13	TEA	CH_3_OH	30	24	57	84:16
14	TEA	C_2_H_5_OH	30	24	74	78:22
15	TEA	toluene	30	24	37	71:29
16	TEA	CH_3_CN	30	24	42	85:15
17	TEA	CH_2_Cl_2_	reflux	24	86	88:12
18 ^d^	TEA	CH_2_Cl_2_	reflux	24	85	88:12

^a^ Unless otherwise noted, all reactions were carried out with **1a** (0.1 mmol), **2a** (0.1 mmol), **3a** (0.11 mmol), and base (0.1 mmol) in solvent (1.0 mL) at specified temperature for 16 or 24 h. DIPEA: diisopropylethylamine, TEA: triethylamine, DCE: 1,2-dichloroethane. ^b^ Isolated yield. ^c^ Determined by ^1^H-NMR spectroscopy of the crude mixture. ^d^ Reaction was carried out with two-fold scale.

**Table 2:** Scope of aldehyde **2** ^a^.

Entry	2,R^2^	4	Yield ^b^ (%)	dr ^c^
1	**2a**,Ph	**4a**	85	88:12
2	**2b**,4-F-Ph	**4b**	80	81:19
3	**2c**,3-F-Ph	**4c**	70	62:38
4	**2d**,2-F-Ph	**4d**	90	92:8
5	**2e**,4-Cl-Ph	**4e**	79	81:19
6	**2f**,3-Cl-Ph	**4f**	71	76:24
7	**2g**,2-Cl-Ph	**4g**	83	81:19
8	**2h**,3,4-diCl-Ph	**4h**	85	86:14
9	**2i**,4-Br-Ph	**4i**	81	>95:5
10	**2j**,3-Br-Ph	**4j**	65	67:33
11	**2k**,2-Br-Ph	**4k**	90	93:7
12	**2l**,4-NO_2_-Ph	**4l**	85	>95:5
13	**2m**,3-NO_2_-Ph	**4m**	83	>95:5
14	**2n**,2-NO_2_-Ph	**4n**	86	94:6
15	**2o**,4-Me-Ph	**4o**	78	82:18
16	**2p**,3-Me-Ph	**4p**	71	75:25
17	**2q**,2-Me-Ph	**4q**	80	77:23
18	**2r**,4-OMe-Ph	**4r**	84	83:17
19	**2s**,3-OMe-Ph	**4s**	76	80:20
20	**2t**,2-naphthyl	**4t**	93	94:6
21	**2u**,2-furyl	**4u**	70	94:6
22	**2v**,2-thienyl	**4v**	79	93:7
23	**2w**,Benzyl	**4w**	76	77:23
24	**2x**,*^t^*Bu	**4x**	72	72:28

^a^ All reactions were carried out with **1a** (0.2mmol), **2** (0.2 mmol), **3a** (0.22 mmol), TEA (0.2 mmol) in CH_2_Cl_2_ (2.0 mL) at reflux for 24 h. ^b^ Isolated yield. ^c^ Determined by ^1^H-NMR analysis of the crude reaction mixture.

**Table 3:** Scope of 3-amino oxindoles **1** and maleimides **3** ^a^.

Entry	1	2,R^2^	3	5	Yield ^b^ (%)	dr ^c^
1	**1b**	**2a**,Ph	**3a**	**5a**	82	83:17
2	**1b**	**2l**,4-NO_2_-Ph	**3a**	**5b**	80	82:18
3	**1c**	**2a**,Ph	**3a**	**5c**	85	75:25
4	**1c**	**2l**,4-NO_2_-Ph	**3a**	**5d**	82	78:22
5	**1d**	**2a**,Ph	**3a**	**5e**	68	69:31
6	**1e**	**2a**,Ph	**3a**	**5f**	80	83:17
7	**1e**	**2l**,4-NO_2_-Ph	**3a**	**5g**	81	82:18
8	**1a**	**2a**,Ph	**3b**	**5h**	66	67:33
9	**1a**	**2l**,4-NO_2_-Ph	**3b**	**5i**	81	82:18
10	**1a**	**2l**,4-NO_2_-Ph	**3c**	**5j**	80	81:19
11	**1a**	**2l**,4-NO_2_-Ph	**3d**	**5k**	80	78:22
12	**1a**	**2l**,4-NO_2_-Ph	**3e**	**5l**	85	84:16
13	**1a**	**2l**,4-NO_2_-Ph	**3f**	**5m**	81	82:18

^a^ All reactions were carried out with **1** (0.2mmol), **2** (0.2 mmol), **3** (0.22 mmol), TEA (0.2 mmol) in CH_2_Cl_2_ (2.0 mL) at reflux for 24 h. ^b^ Isolated yield. ^c^ Determined by ^1^H-NMRanalysis of the crude reaction mixture.

## Supplementary Materials

The NMR spectra of the products (**4** and **5**) are available online; the crystallographic data of **4k** is available online.
