# Supplementary material for: A Facile One-Pot Construction of Succinimide-Fused Spiro[Pyrrolidine-2,3′-Oxindoles] via 1,3-Dipolar Cycloaddition Involving 3-Amino Oxindoles and Maleimides
Source: Molecules. 2018 Mar 5;23(3):582. doi: 10.3390/molecules23030582 (PMC6017913; doi:10.3390/molecules23030582)
Supplement: Supplementary file 1 [file molecules-23-00582-s001.pdf]

Supplementary Materials

A facile one-pot construction of succimide-fused spiro[pyrrolidine-2,3'-oxindoles] via 1,3-dipolar cycloaddition involving 3-amino oxindoles and maleimides

Lunqiang Jin, Feng Liang*

The State Key Laboratory of Refractories and Metallurgy, School of Chemistry & Chemical Engineering,Wuhan University of Science and Technology, Wuhan 430081, China

NMR Spectra

4a

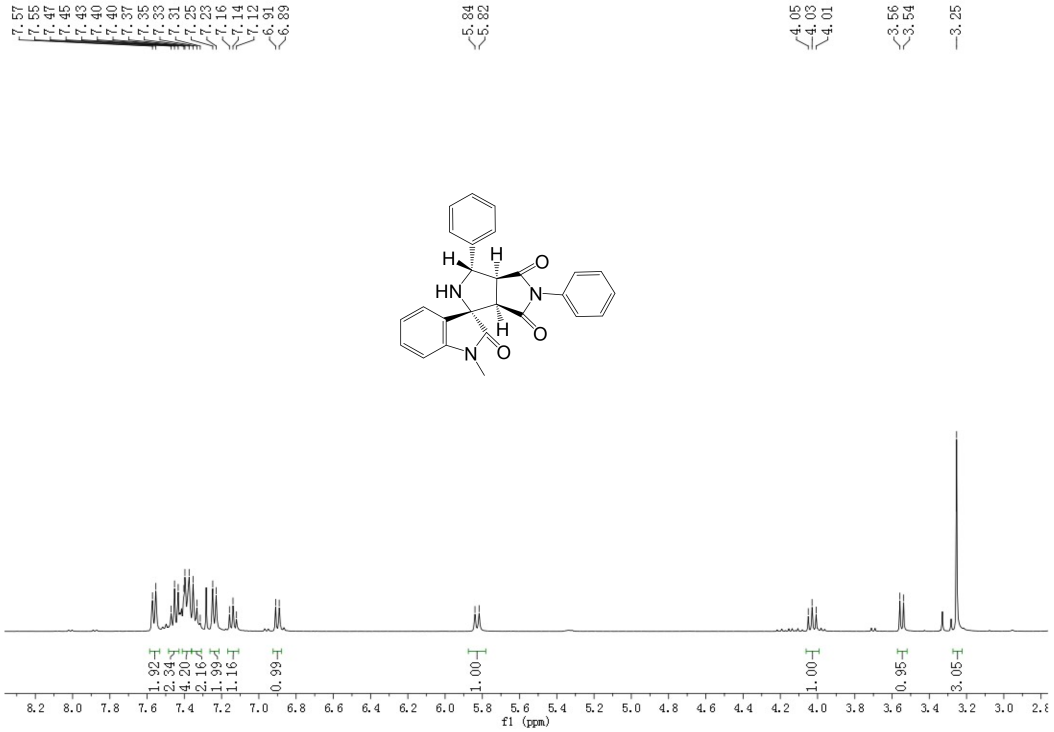

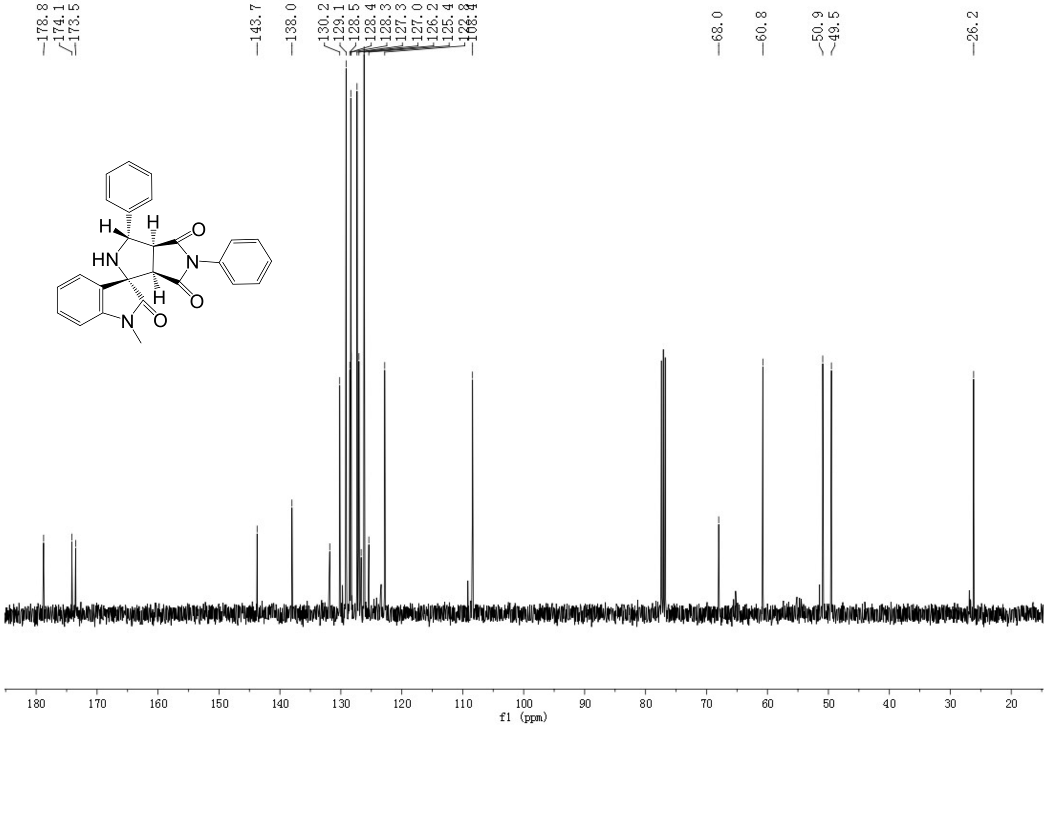

4b

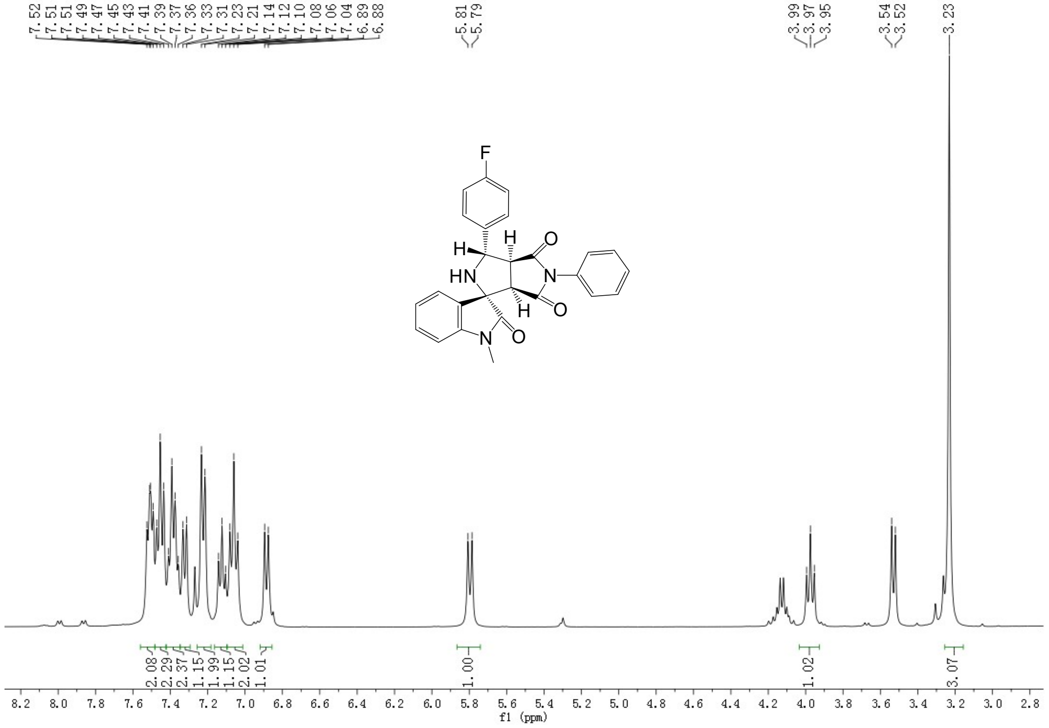
7.52
7.51
7.51
7.49
7.47
7.45
7.43
7.41
7.39
7.37
7.36
7.33
7.31
7.23
7.21
7.14
7.12
7.10
7.08
7.06
7.04
6.89
6.88
5.81
5.79
3.99
3.97
3.95
3.54
3.52
3.23
F
H
H
O
HN
N
H
O
N
O
2.08
2.29
2.37
1.15
1.99
1.15
2.02
1.01
1.00
1.02
3.07
8.2 8.0 7.8 7.6 7.4 7.2 7.0 6.8 6.6 6.4 6.2 6.0 5.8 5.6 5.4 5.2 5.0 4.8 4.6 4.4 4.2 4.0 3.8 3.6 3.4 3.2 3.0 2.8
f1 (ppm)

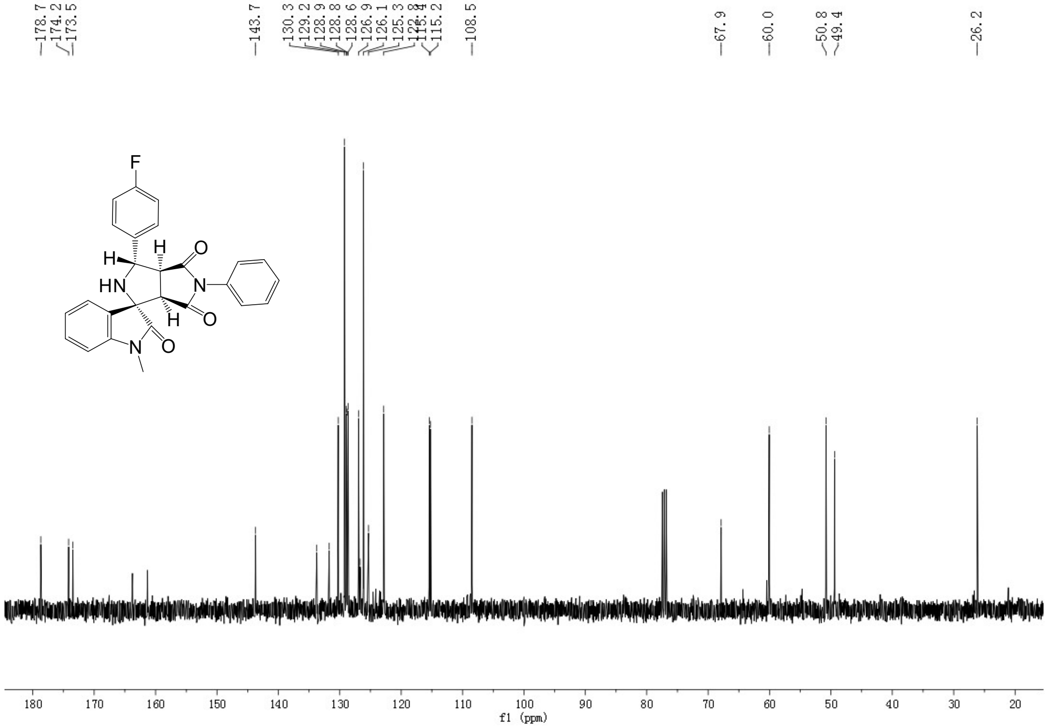
178.7
174.2
173.5
143.7
130.3
129.2
128.9
128.8
128.6
126.9
126.1
125.3
115.2
108.5
67.9
60.0
50.8
49.4
26.2
F
H
H
O
HN
N
H
O
N
O
180 170 160 150 140 130 120 110 100 90 80 70 60 50 40 30 20
f1 (ppm)

4c

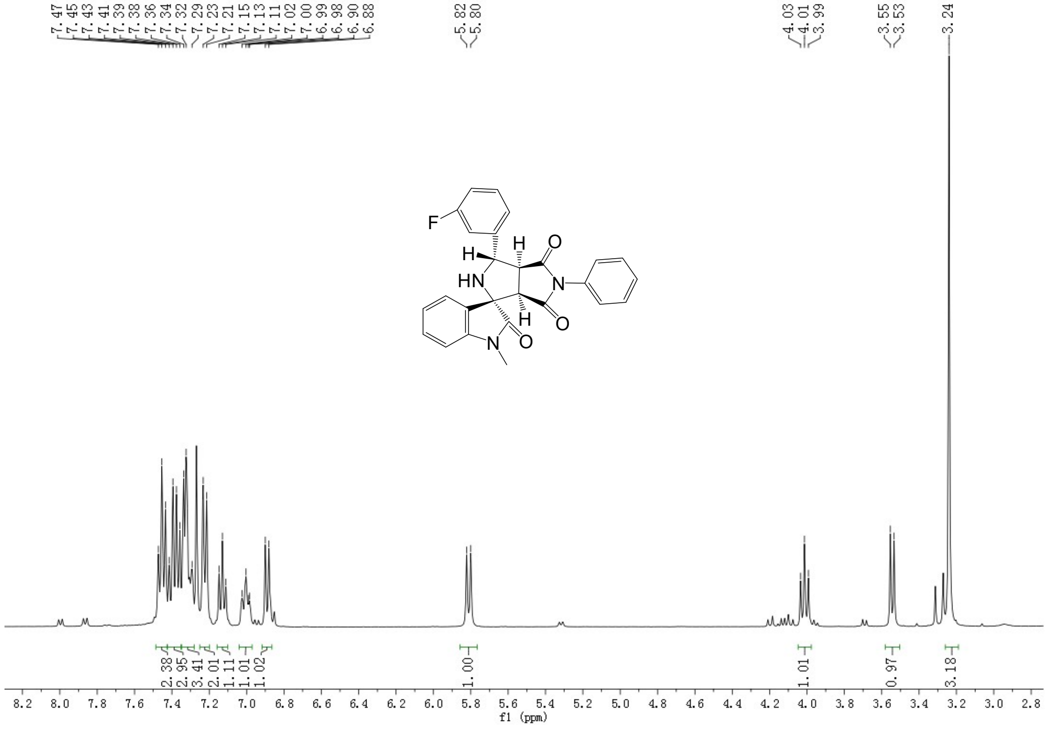
7.47
7.45
7.43
7.41
7.39
7.38
7.36
7.34
7.32
7.29
7.23
7.21
7.15
7.13
7.11
7.02
7.00
6.99
6.98
6.90
6.88
5.82
5.80
4.03
4.01
3.99
3.55
3.53
3.24
F
H
H
O
HN
N
H
O
O
N
2.38
2.95
3.41
2.01
1.11
1.01
1.02
1.00
1.01
0.97
3.18
f1 (ppm)

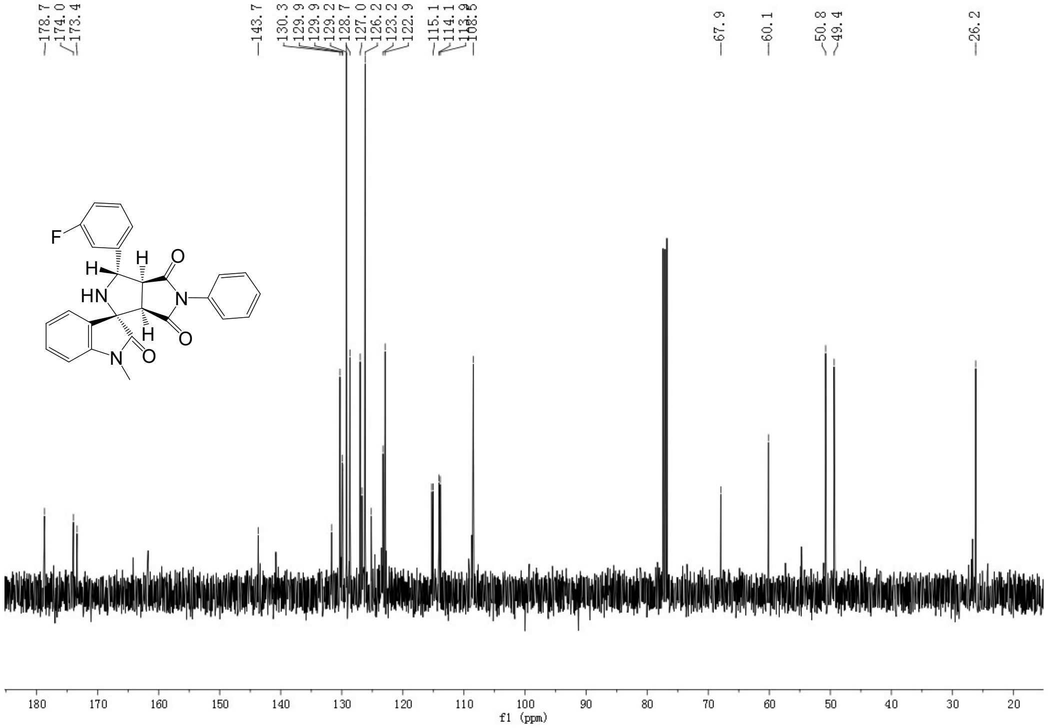
178.7
174.0
173.4
143.7
130.3
129.9
129.9
129.2
128.7
127.0
126.2
123.2
122.9
115.1
114.1
113.9
108.5
67.9
60.1
50.8
49.4
26.2
F
H
H
O
HN
N
H
O
O
N
f1 (ppm)

4d

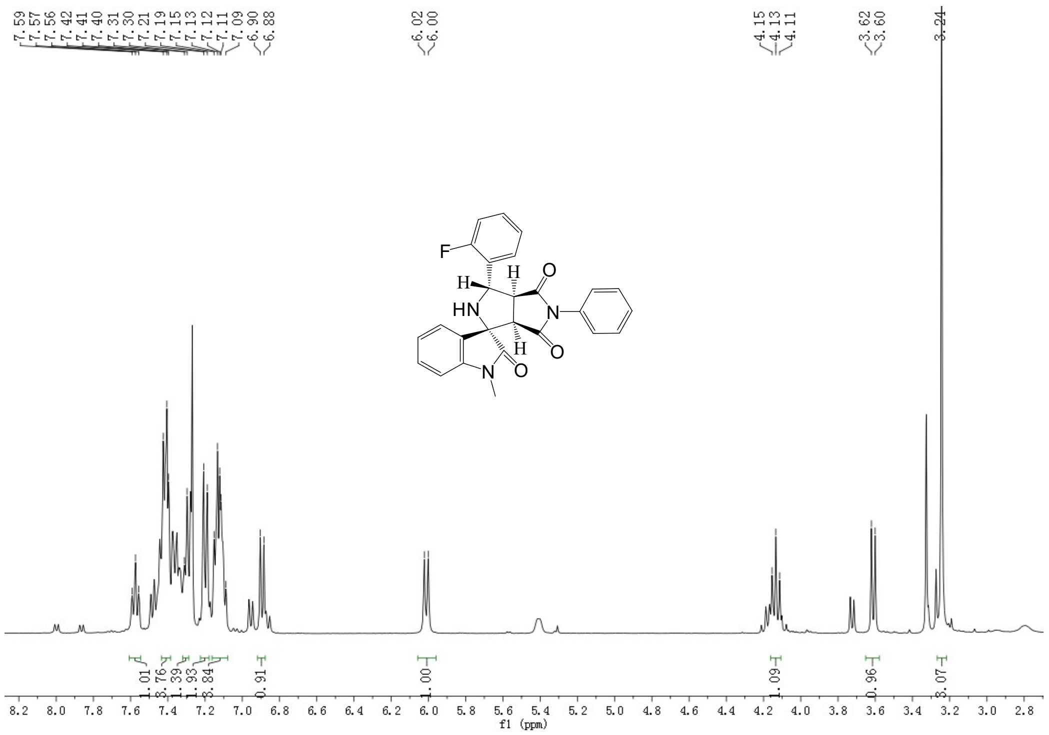
7.59
7.57
7.56
7.42
7.41
7.40
7.31
7.30
7.21
7.19
7.15
7.13
7.12
7.11
7.09
6.90
6.88
6.02
6.00
4.15
4.13
4.11
3.62
3.60
3.24
F
H
HN
N
O
1.01
3.76
1.39
1.93
3.84
0.91
1.00
1.09
0.96
3.07
8.2 8.0 7.8 7.6 7.4 7.2 7.0 6.8 6.6 6.4 6.2 6.0 5.8 5.6 5.4 5.2 5.0 4.8 4.6 4.4 4.2 4.0 3.8 3.6 3.4 3.2 3.0 2.8
f1 (ppm)

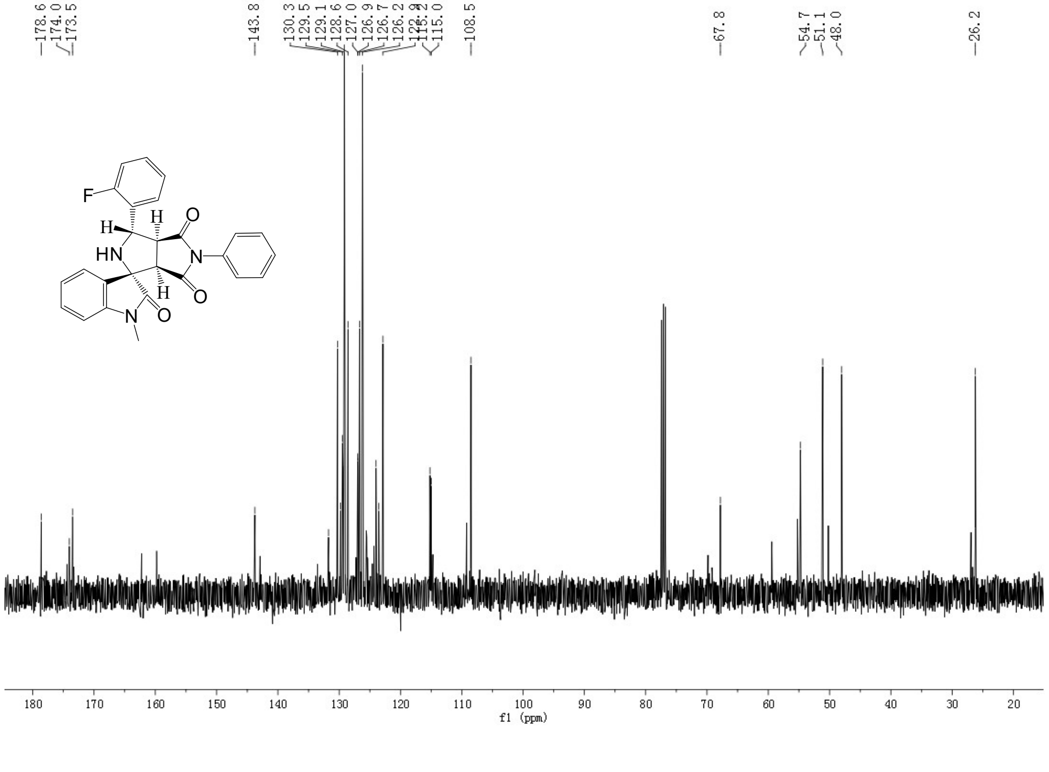
178.6
174.0
173.5
143.8
130.3
129.5
129.1
128.6
127.0
126.9
126.7
126.2
115.0
108.5
67.8
54.7
51.1
48.0
26.2
F
H
HN
N
O
180 170 160 150 140 130 120 110 100 90 80 70 60 50 40 30 20
f1 (ppm)

4e

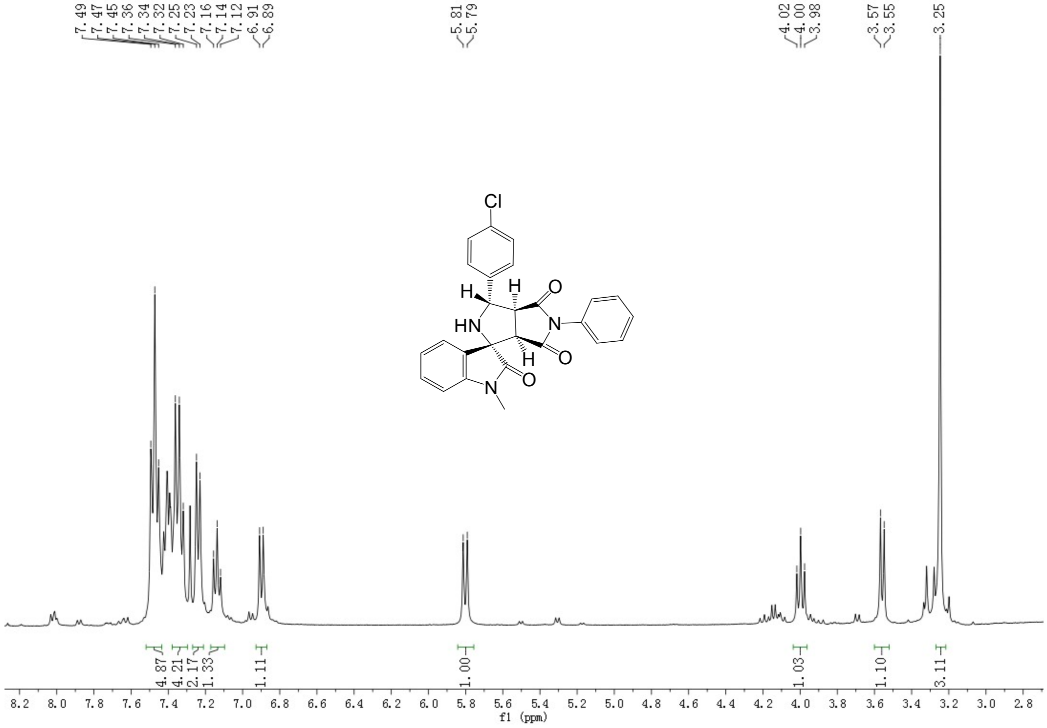

7.49
7.47
7.45
7.36
7.34
7.32
7.25
7.23
7.16
7.14
7.12
6.91
6.89
5.81
5.79
4.02
4.00
3.98
3.57
3.55
3.25
Cl
H
H
HN
H
N
O
O
O
N
4.87
4.21
2.17
1.33
1.11
1.00
1.03
1.10
3.11
f1 (ppm)

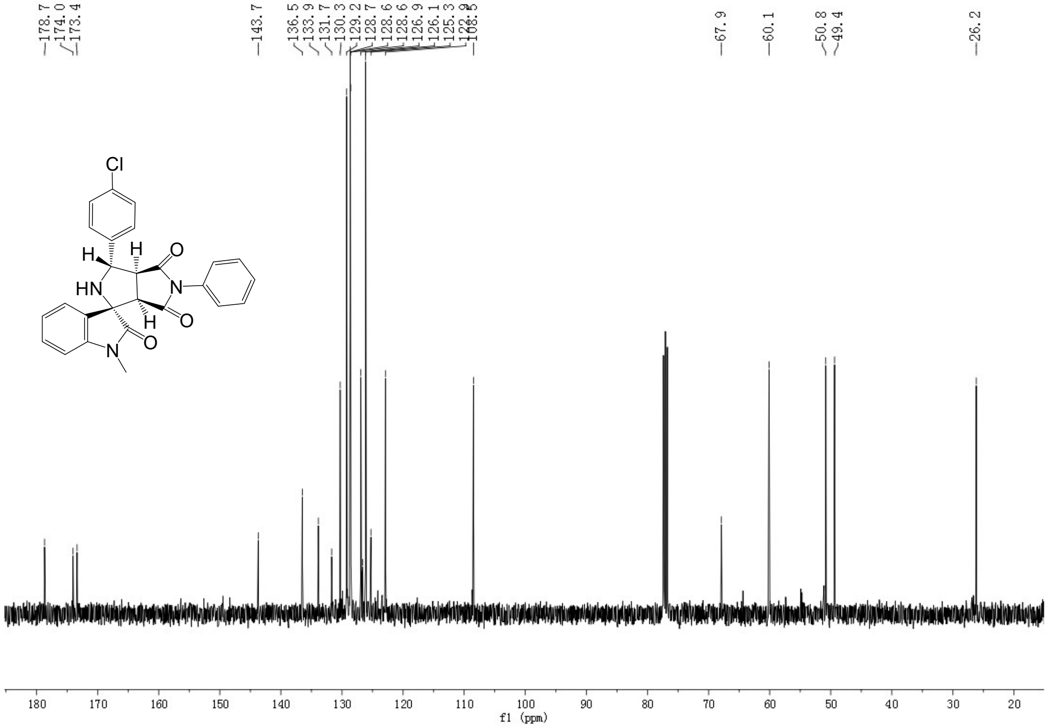

178.7
174.0
173.4
143.7
136.5
133.9
131.7
130.3
129.2
128.7
128.6
126.9
126.1
125.3
122.9
108.5
67.9
60.1
50.8
49.4
26.2
Cl
H
H
HN
H
N
O
O
O
N
f1 (ppm)

4f

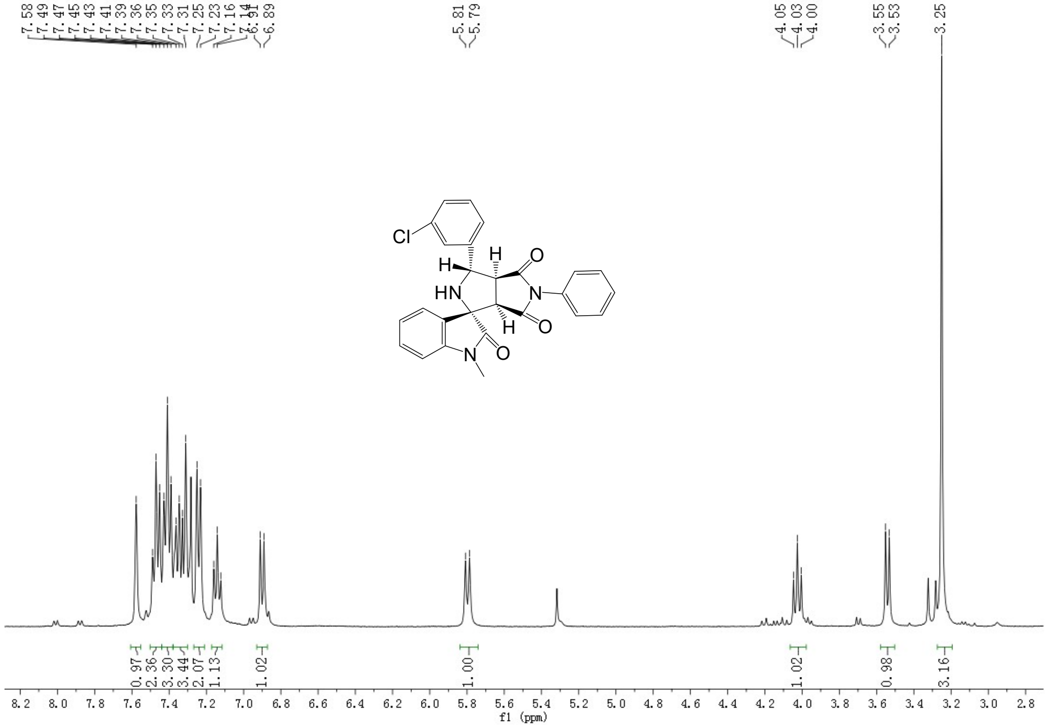
7.58
7.49
7.47
7.45
7.43
7.41
7.39
7.36
7.35
7.33
7.31
7.25
7.23
7.16
7.14
6.91
6.89
5.81
5.79
4.05
4.03
4.00
3.55
3.53
3.25
Cl
H
H
HN
N
O
O
H
N
O
0.97
2.36
3.30
3.44
2.07
1.13
1.02
1.00
1.02
0.98
3.16
f1 (ppm)

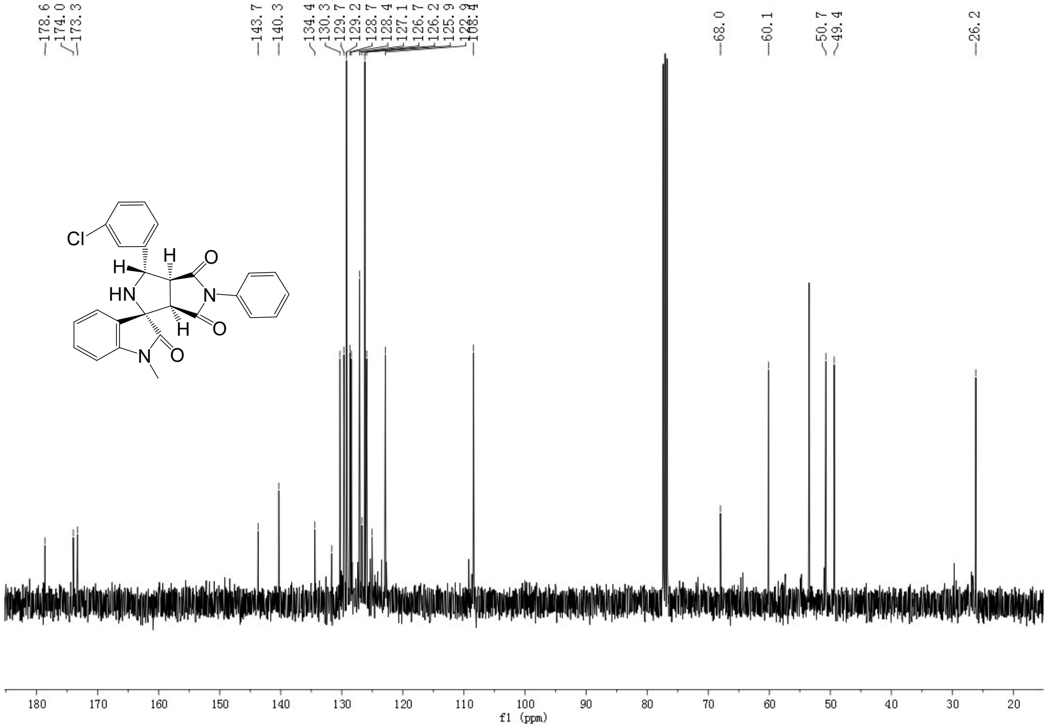
178.6
174.0
173.3
143.7
140.3
134.4
130.3
129.7
129.2
128.7
128.4
127.1
126.7
126.2
125.9
122.9
108.4
68.0
60.1
50.7
49.4
26.2
Cl
H
H
HN
N
O
O
H
N
O
f1 (ppm)

4g

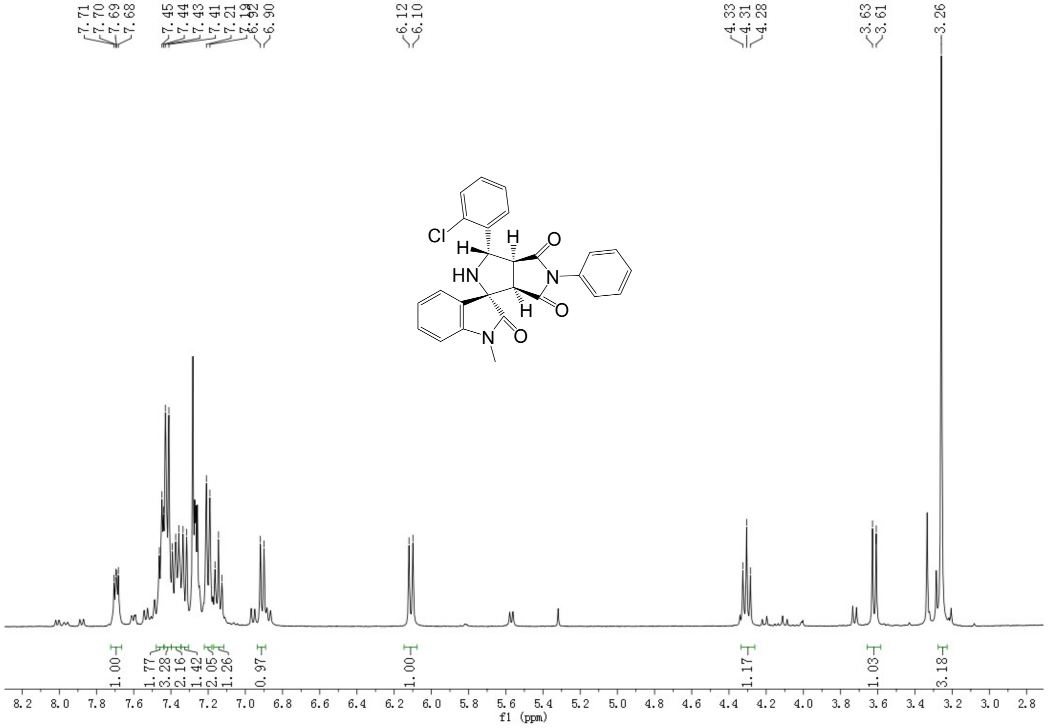
7.71
7.70
7.69
7.68
7.45
7.44
7.43
7.41
7.21
7.19
6.92
6.90
6.12
6.10
4.33
4.31
4.28
3.63
3.61
3.26
Cl
H
H
O
HN
N
H
O
N
O
1.00
1.77
3.28
2.16
1.42
2.05
1.26
0.97
1.00
1.17
1.03
3.18
8.2 8.0 7.8 7.6 7.4 7.2 7.0 6.8 6.6 6.4 6.2 6.0 5.8 5.6 5.4 5.2 5.0 4.8 4.6 4.4 4.2 4.0 3.8 3.6 3.4 3.2 3.0 2.8
f1 (ppm)

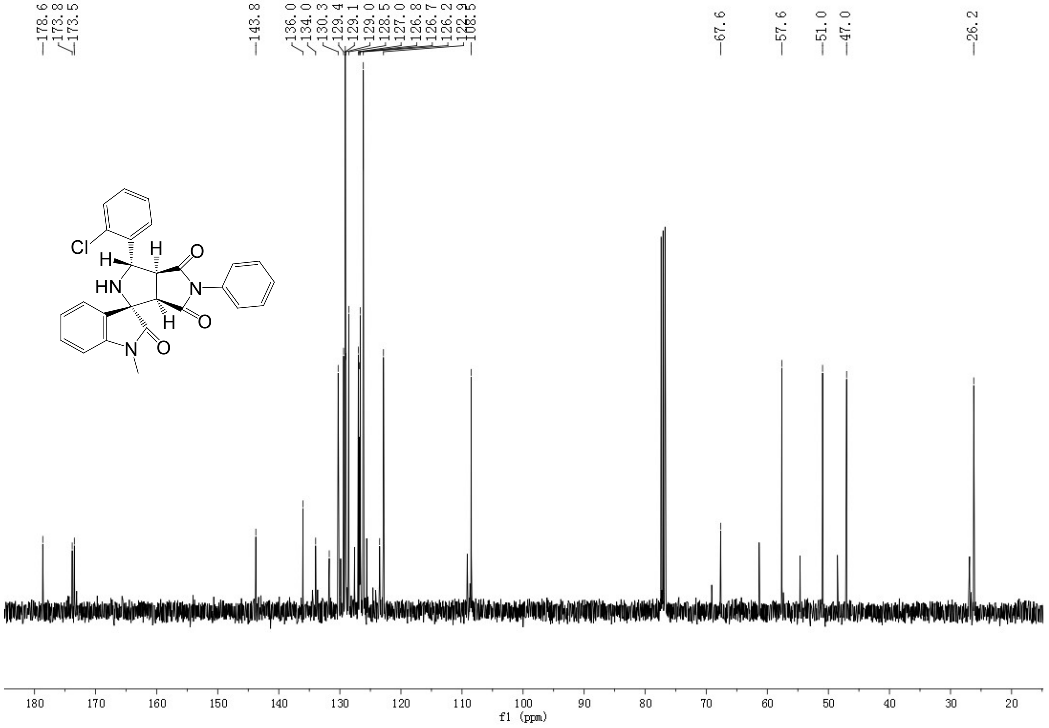
178.6
173.8
173.5
143.8
136.0
134.0
130.3
129.4
129.1
129.0
128.5
127.0
126.8
126.7
126.2
108.5
67.6
57.6
51.0
47.0
26.2
Cl
H
H
O
HN
N
H
O
N
O
180 170 160 150 140 130 120 110 100 90 80 70 60 50 40 30 20
f1 (ppm)

4h

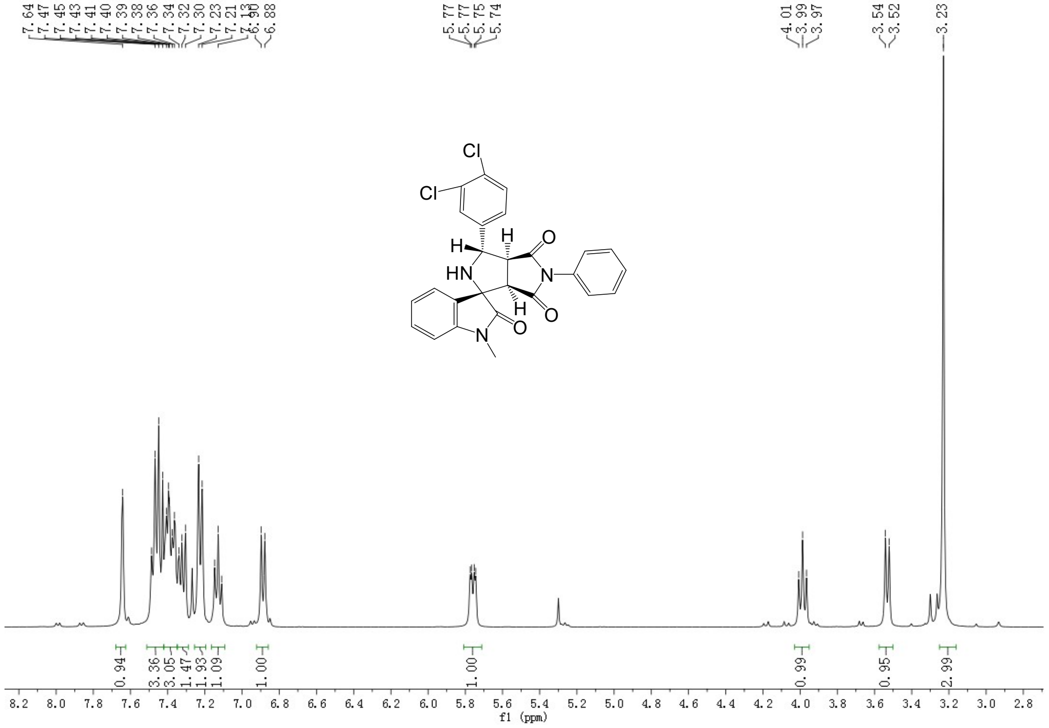
7.64
7.47
7.45
7.43
7.41
7.40
7.39
7.38
7.36
7.34
7.32
7.30
7.23
7.21
7.13
6.90
6.88
5.77
5.77
5.75
5.74
4.01
3.99
3.97
3.54
3.52
3.23
Cl
Cl
H
H
O
HN
N
H
O
N
O
0.94
3.36
3.05
1.47
1.93
1.09
1.00
1.00
0.99
0.95
2.99
f1 (ppm)

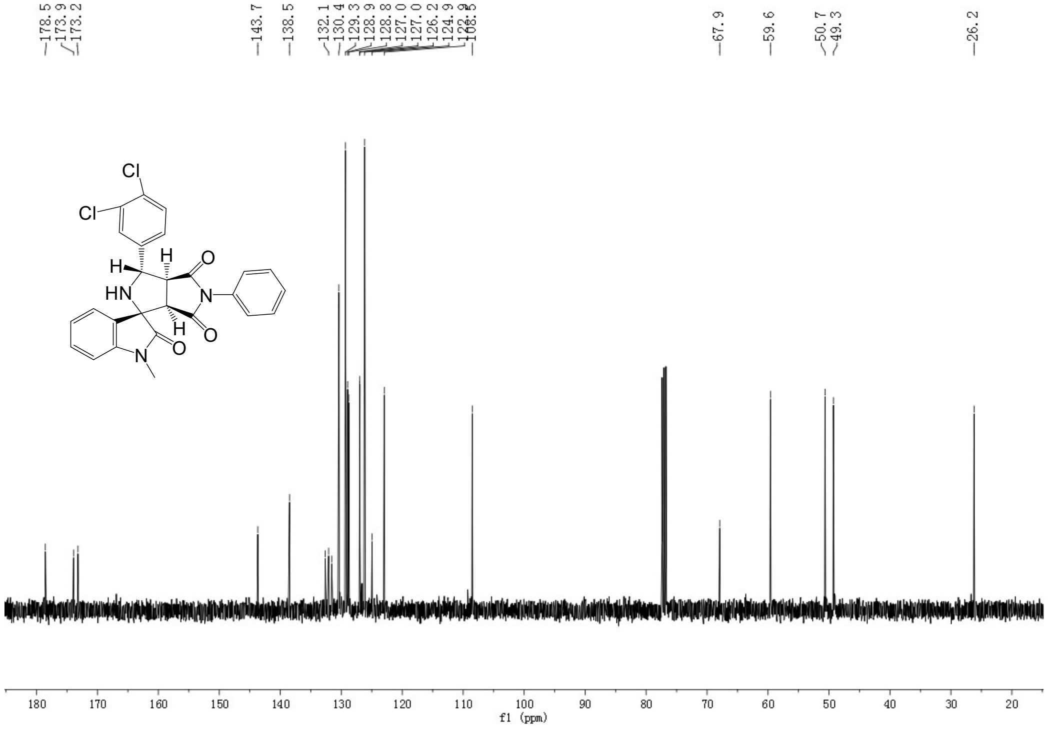
178.5
173.9
173.2
143.7
138.5
132.1
130.4
129.3
128.9
128.8
127.0
126.2
124.9
122.9
108.5
67.9
59.6
50.7
49.3
26.2
Cl
Cl
H
H
O
HN
N
H
O
N
O
f1 (ppm)

4i

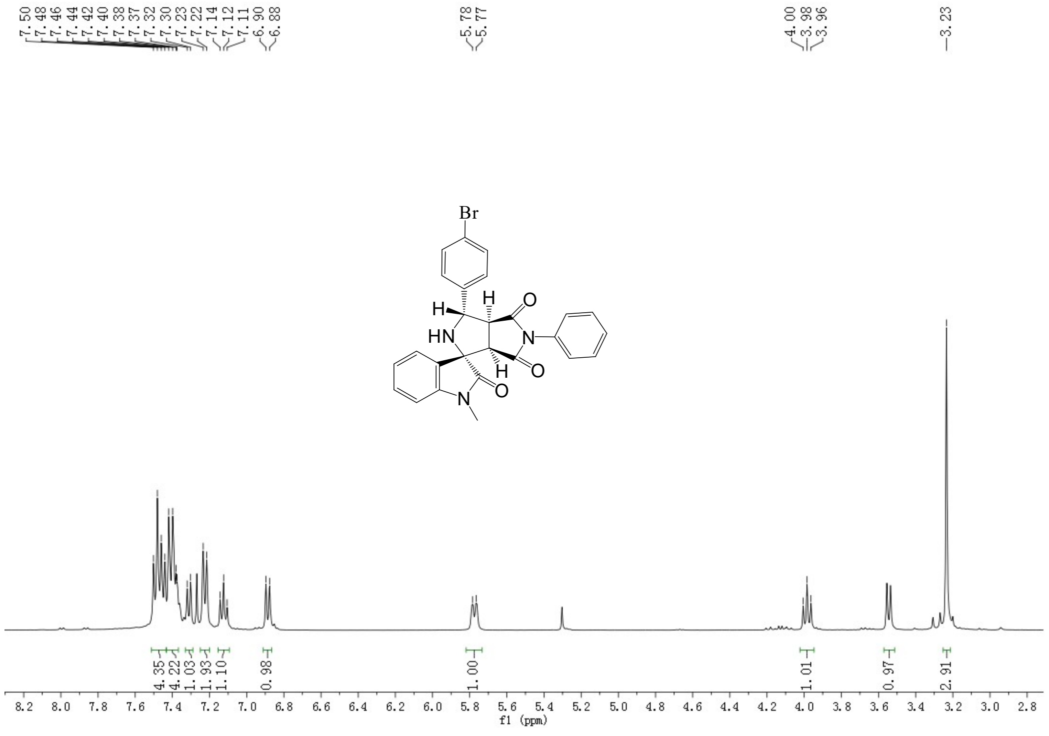
7.50 7.48 7.46 7.44 7.42 7.40 7.38 7.37 7.32 7.30 7.23 7.22 7.14 7.12 7.11 6.90 6.88
5.78 5.77
4.00 3.98 3.96
3.23
Br
H
HN
N
O
4.35 4.22 1.03 1.93 1.10 0.98 1.00 1.01 0.97 2.91
f1 (ppm)

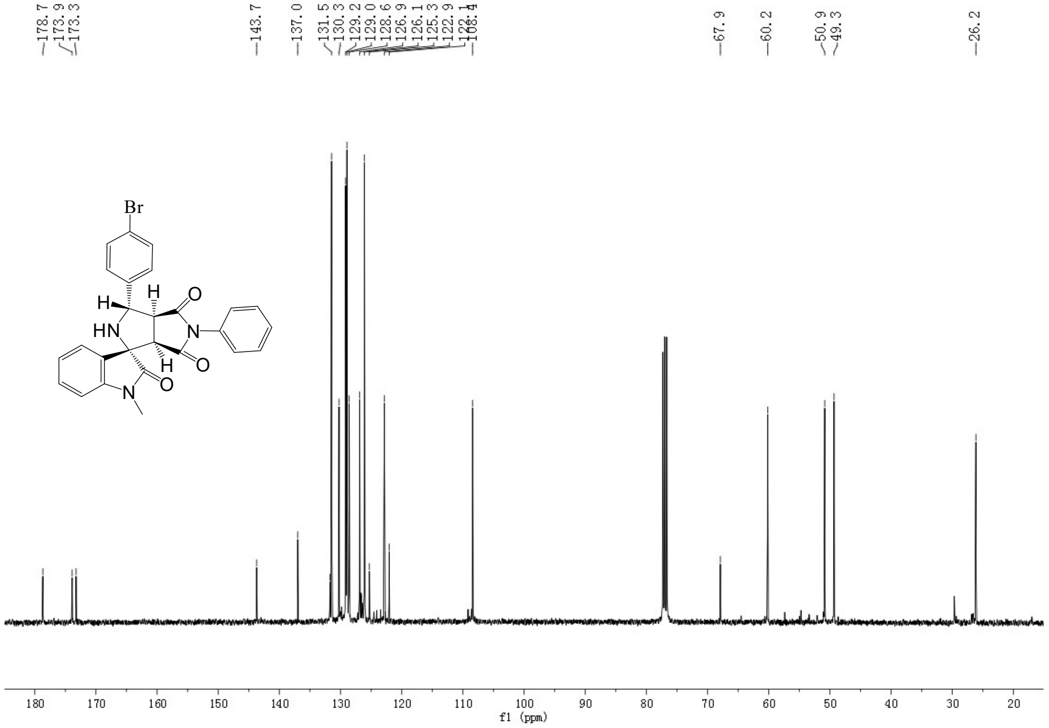
178.7 173.9 173.3
143.7
137.0
131.5 130.3 129.2 129.0 128.6 126.9 126.1 125.3 122.9 122.1 108.4
67.9
60.2
50.9 49.3
26.2
Br
H
HN
N
O
f1 (ppm)

4j

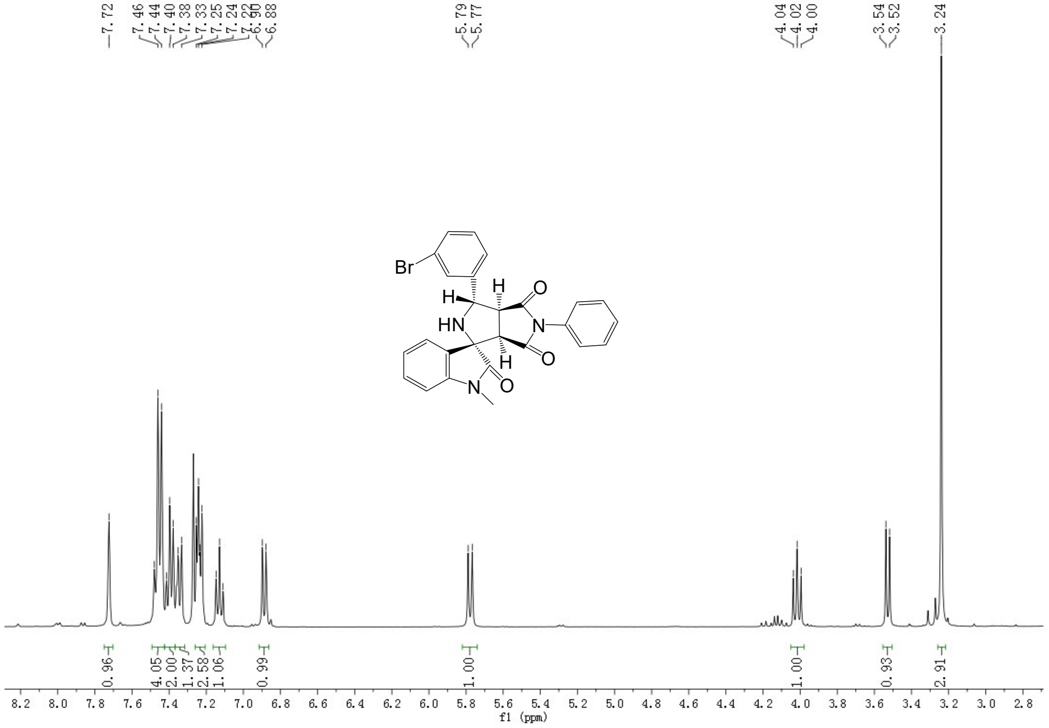
7.72
7.46
7.44
7.40
7.38
7.33
7.25
7.24
7.22
6.90
6.88
5.79
5.77
4.04
4.02
4.00
3.54
3.52
3.24
0.96
4.05
2.00
1.37
2.58
1.06
0.99
1.00
1.00
0.93
2.91
f1 (ppm)

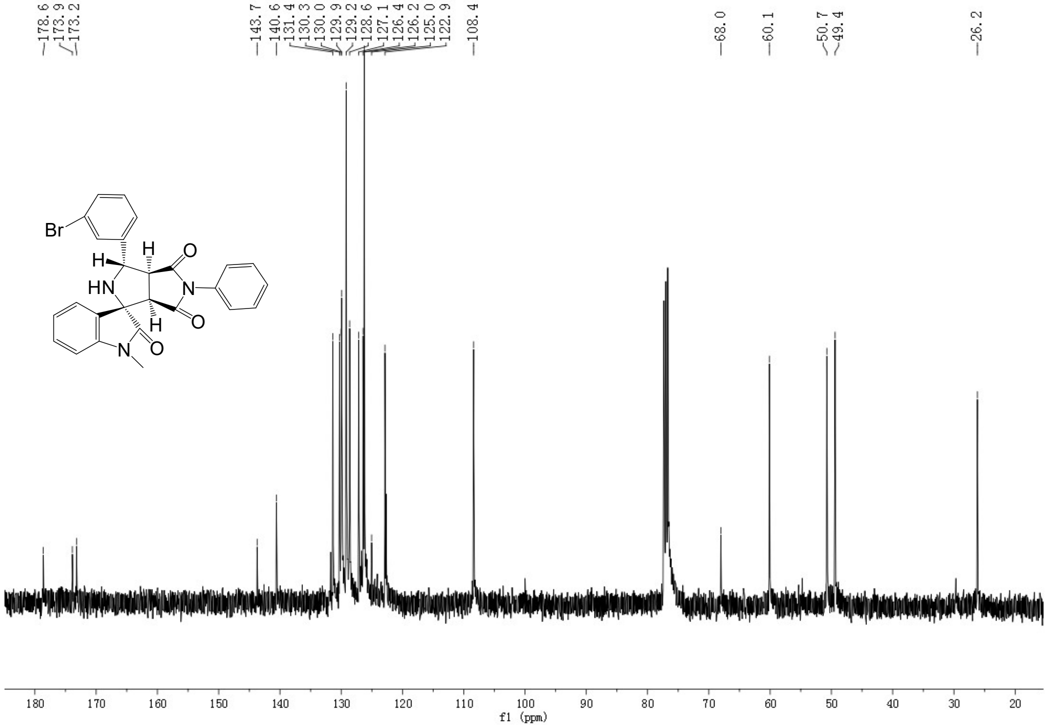
178.6
173.9
173.2
143.7
140.6
131.4
130.3
130.0
129.9
129.2
128.6
127.1
126.4
126.2
125.0
122.9
108.4
68.0
60.1
50.7
49.4
26.2
f1 (ppm)

4k

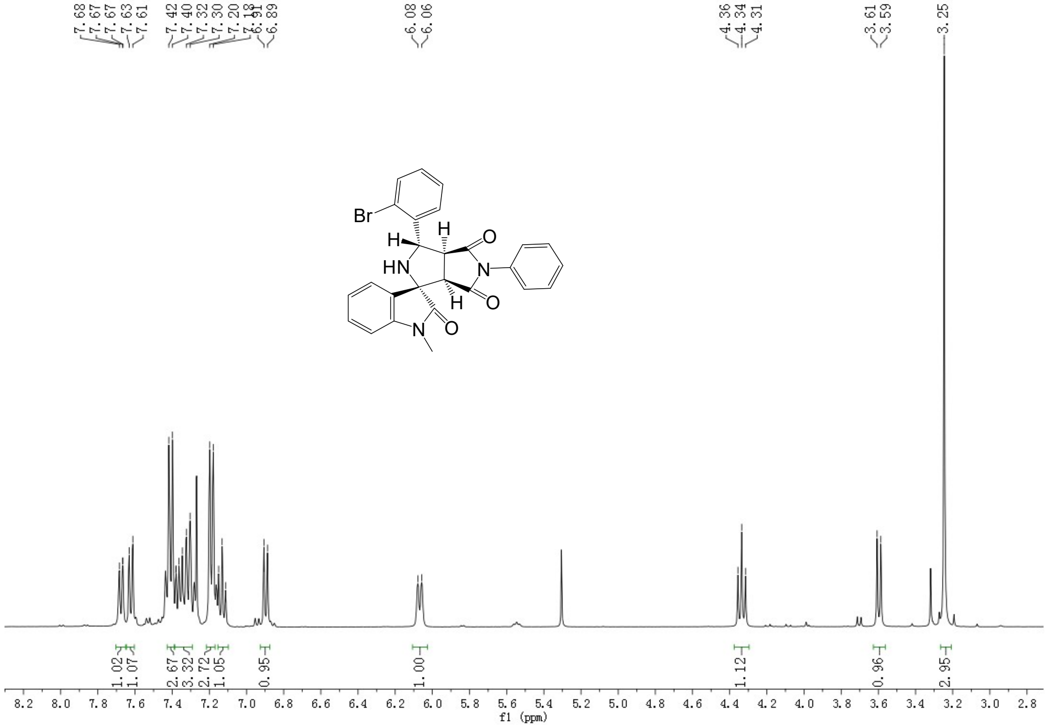

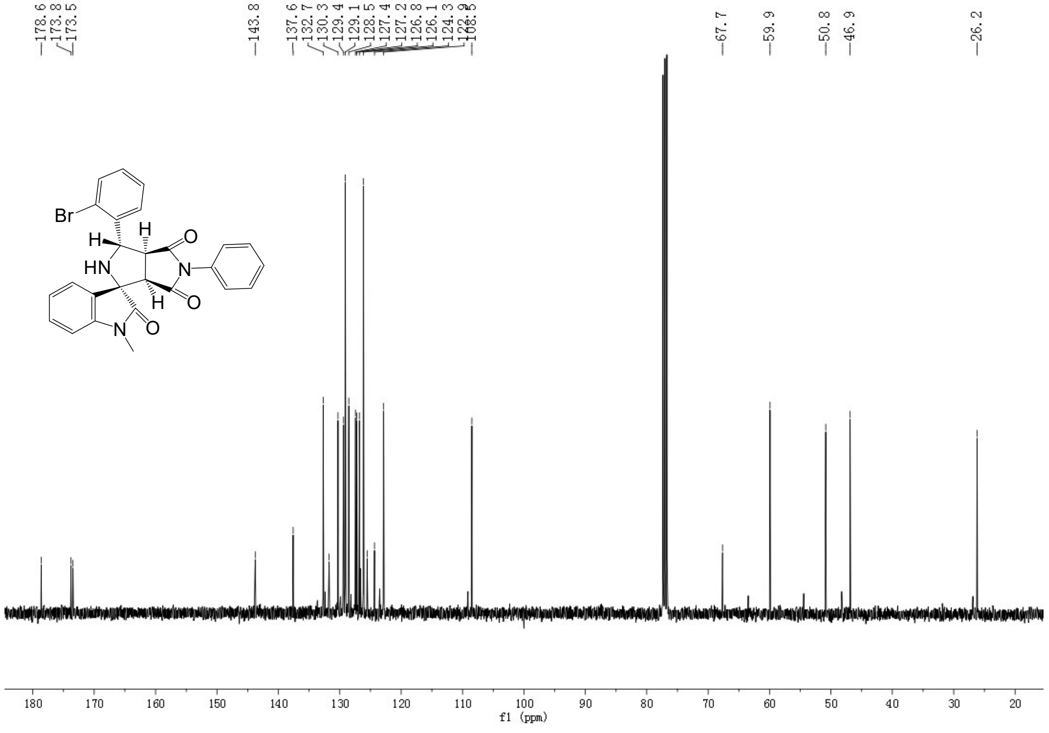

4l

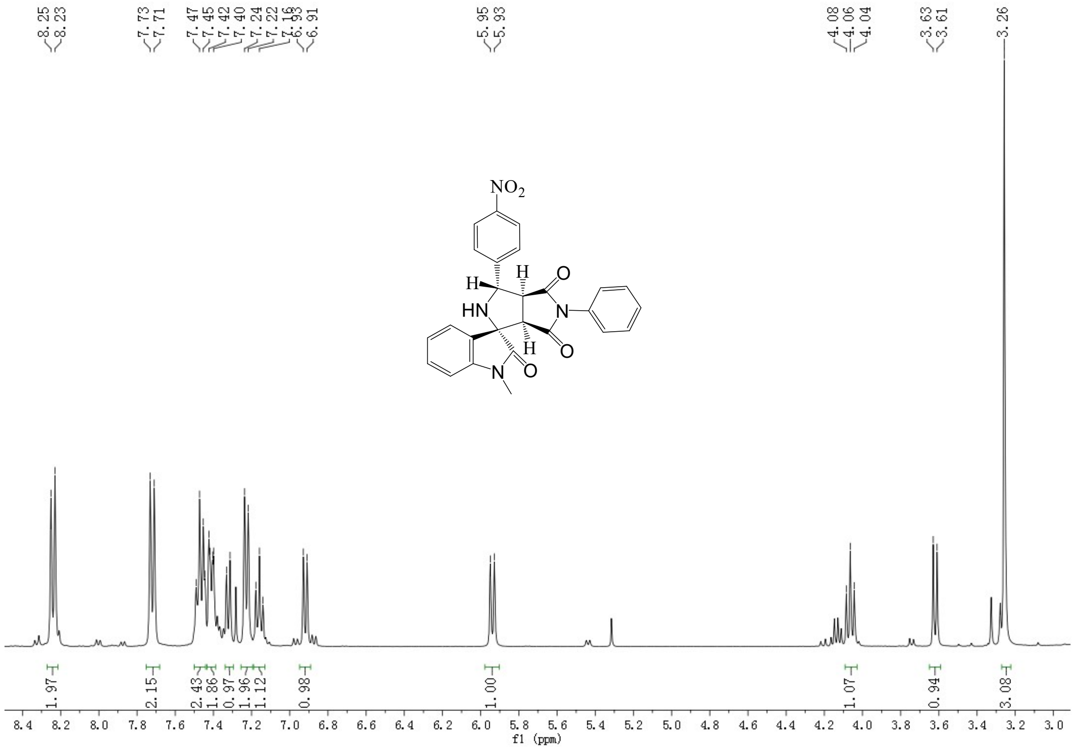
8.25
8.23
7.73
7.71
7.47
7.45
7.42
7.40
7.24
7.22
7.18
6.93
6.91
5.95
5.93
4.08
4.06
4.04
3.63
3.61
3.26
NO_2
H
H
HN
N
O
O
H
N
O
1.97
2.15
2.43
1.86
0.97
1.96
1.12
0.98
1.00
1.07
0.94
3.08
f1 (ppm)

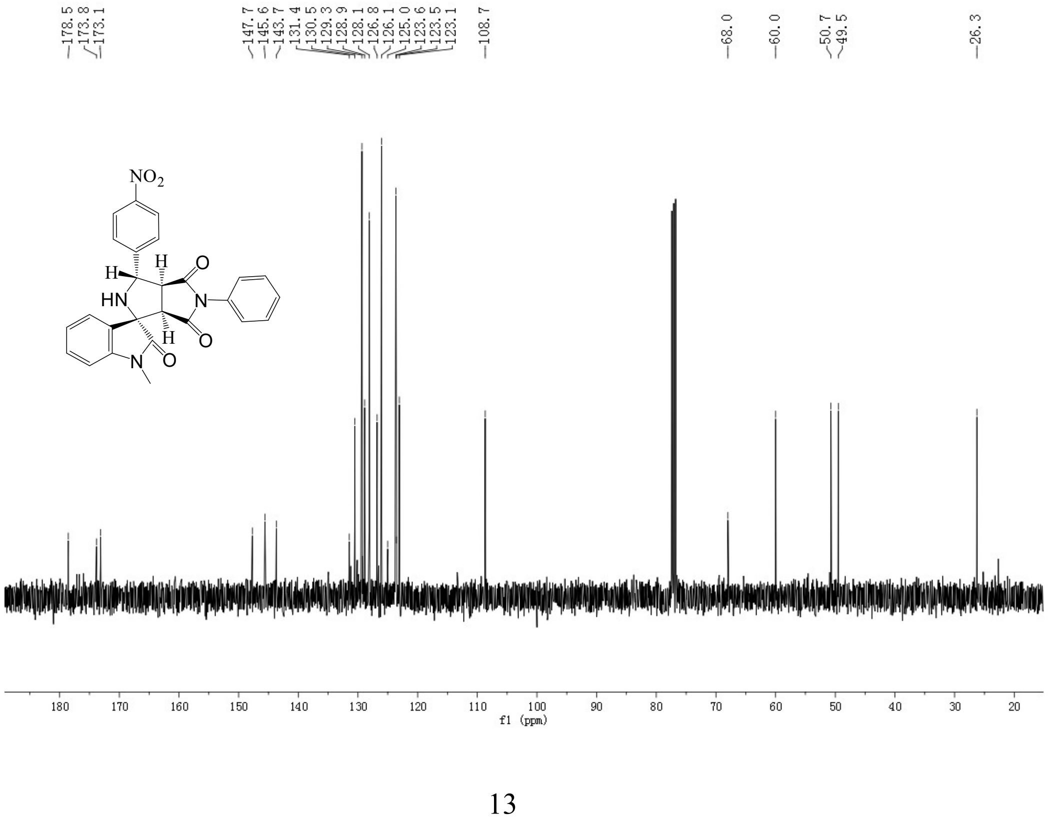
178.5
173.8
173.1
147.7
145.6
143.7
131.4
130.5
129.3
128.9
128.1
126.8
126.1
125.0
123.6
123.5
123.1
108.7
68.0
60.0
50.7
49.5
26.3
NO_2
f1 (ppm)

4m

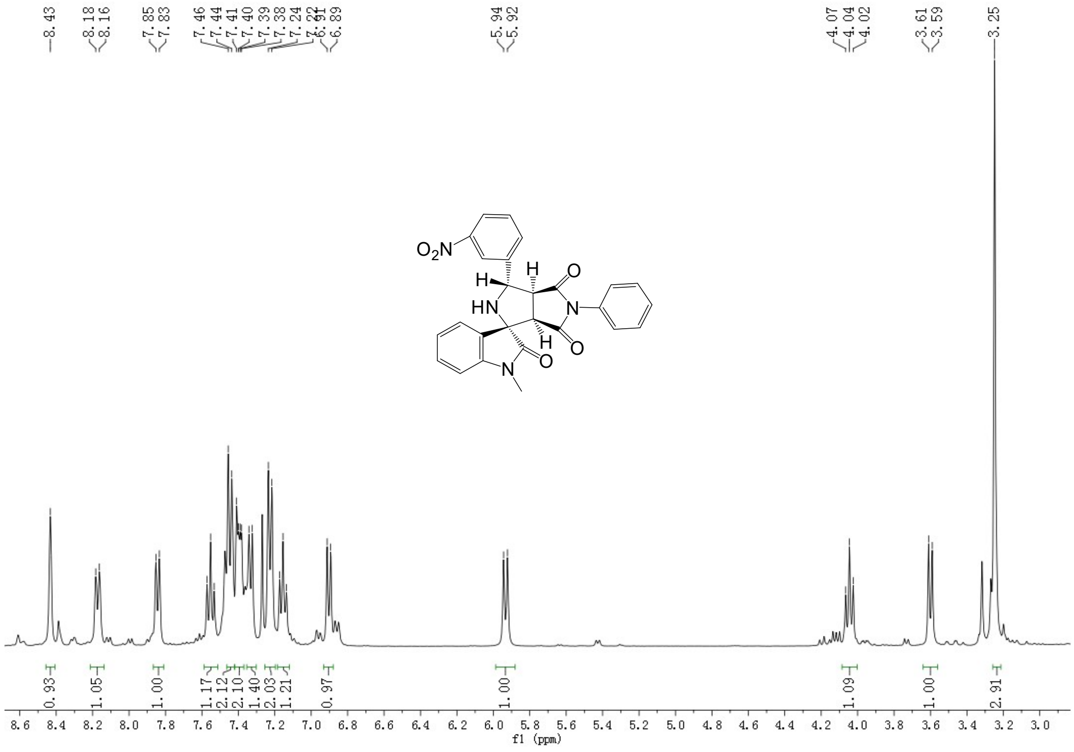
8.43
8.18
8.16
7.85
7.83
7.46
7.44
7.41
7.40
7.39
7.38
7.24
7.22
6.91
6.89
5.94
5.92
4.07
4.04
4.02
3.61
3.59
3.25
O_2N
H
HN
N
O
0.93
1.05
1.00
1.17
2.12
2.10
1.40
2.03
1.21
0.97
1.00
1.09
1.00
2.91
f1 (ppm)

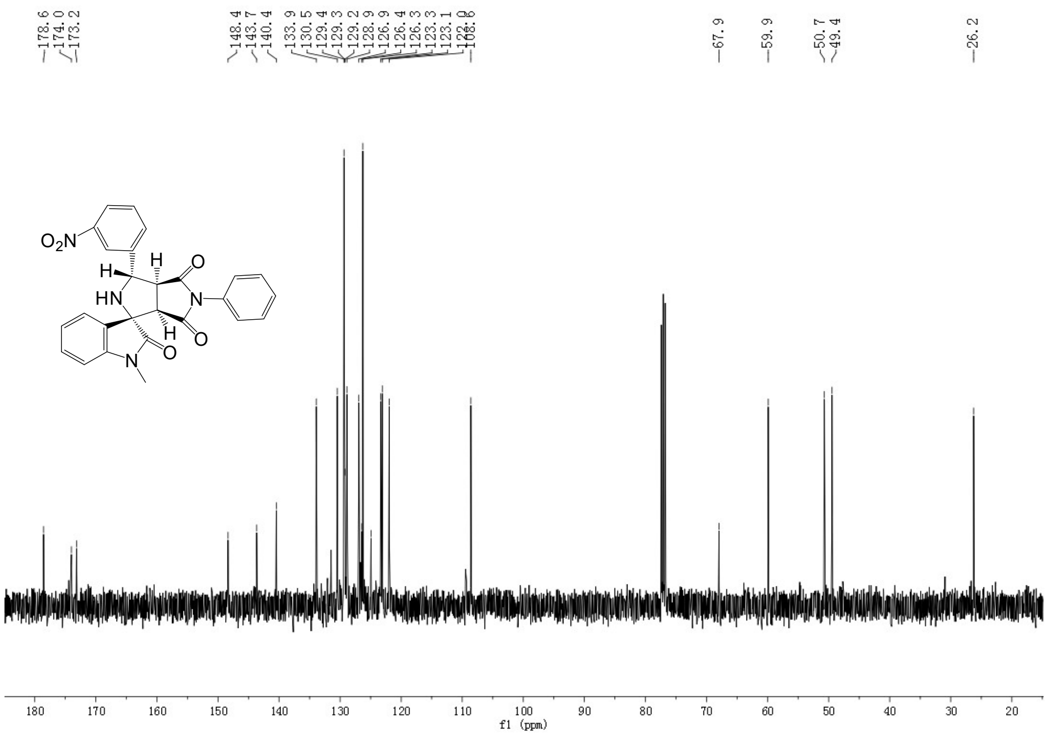
178.6
174.0
173.2
148.4
143.7
140.4
133.9
130.5
129.4
129.3
129.2
128.9
126.9
126.4
126.3
123.3
123.1
O_2N
H
HN
N
O
67.9
59.9
50.7
49.4
26.2
f1 (ppm)

4n

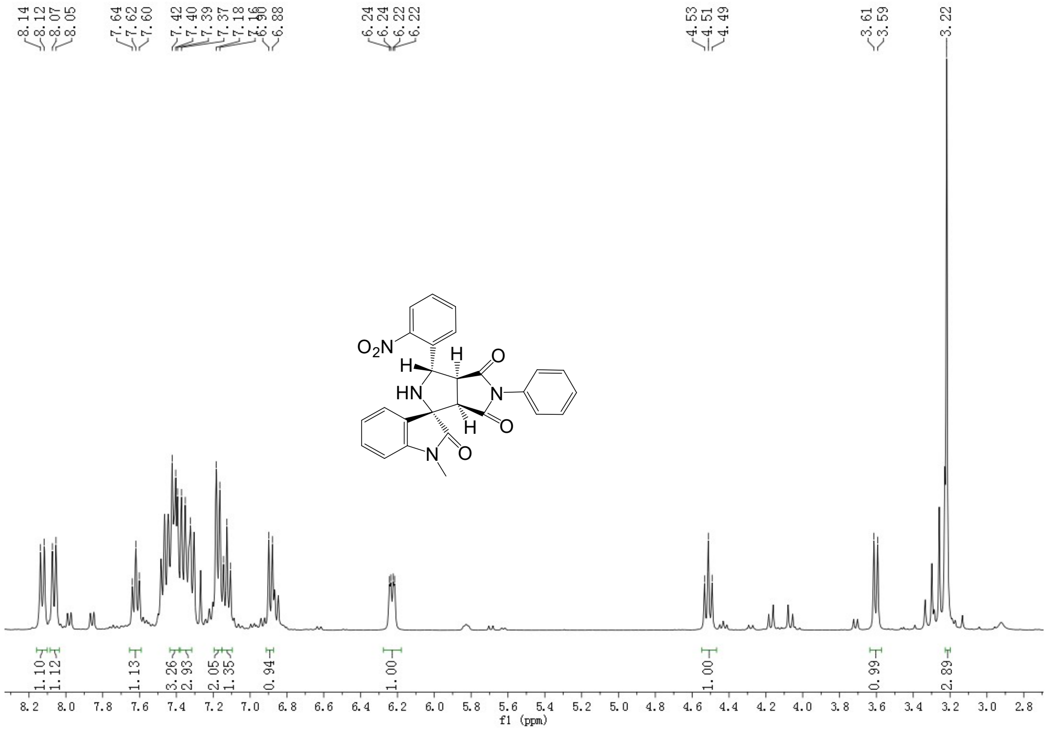
8.14
8.12
8.07
8.05
7.64
7.62
7.60
7.42
7.40
7.39
7.37
7.18
7.16
6.90
6.88
6.24
6.24
6.22
6.22
4.53
4.51
4.49
3.61
3.59
3.22
O_2N
H
HN
H
O
N
H
O
N
O
1.10
1.12
1.13
3.26
2.93
2.05
1.35
0.94
1.00
1.00
0.99
2.89
8.2 8.0 7.8 7.6 7.4 7.2 7.0 6.8 6.6 6.4 6.2 6.0 5.8 5.6 5.4 5.2 5.0 4.8 4.6 4.4 4.2 4.0 3.8 3.6 3.4 3.2 3.0 2.8
f1 (ppm)

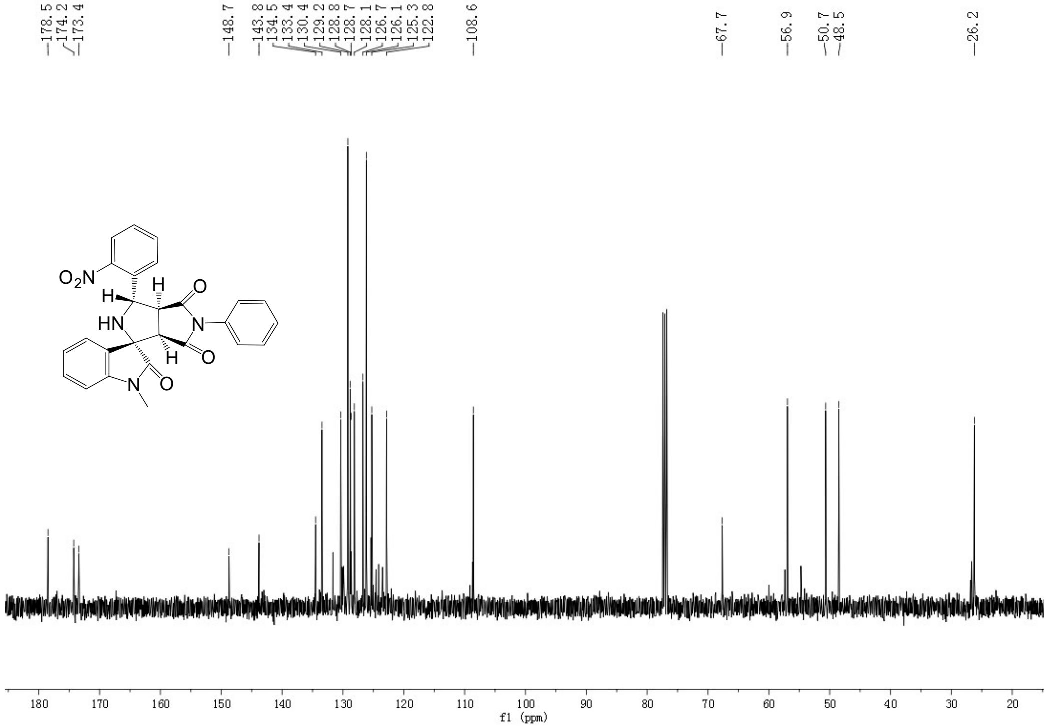
178.5
174.2
173.4
148.7
143.8
134.5
133.4
130.4
129.2
128.8
128.7
128.1
126.7
126.1
125.3
122.8
108.6
67.7
56.9
50.7
48.5
26.2
O_2N
H
H
O
HN
N
H
O
N
O
180 170 160 150 140 130 120 110 100 90 80 70 60 50 40 30 20
f1 (ppm)

4o

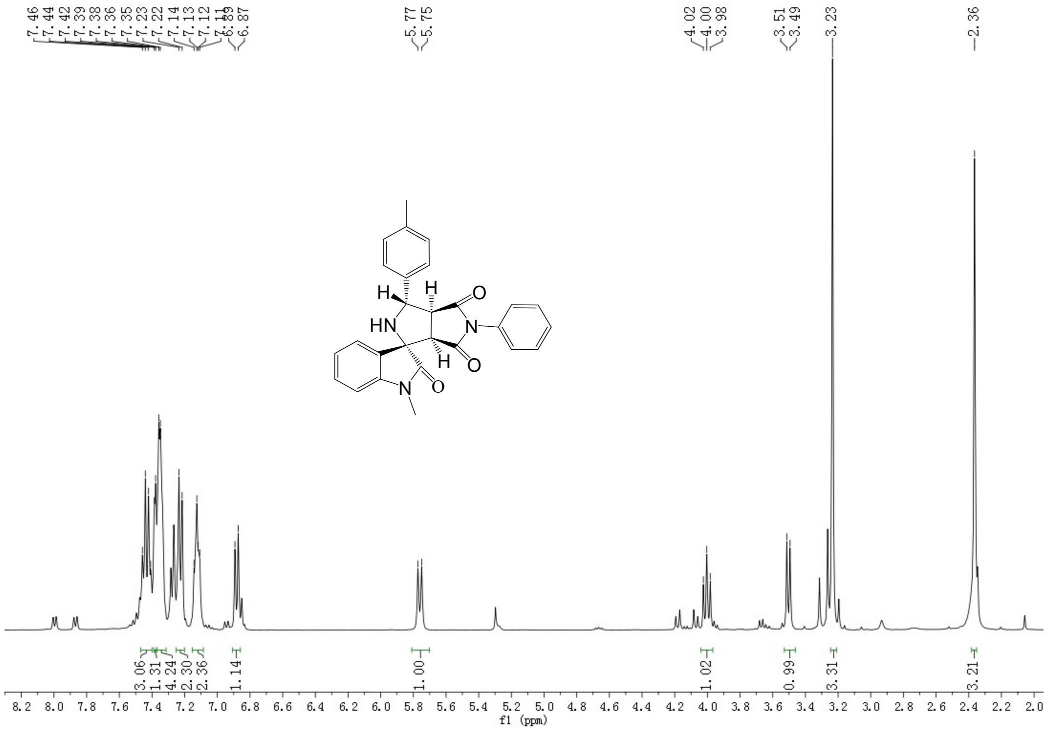
7.46
7.44
7.42
7.39
7.38
7.36
7.35
7.23
7.22
7.14
7.13
7.12
7.11
6.89
6.87
5.77
5.75
4.02
4.00
3.98
3.51
3.49
3.23
2.36
H
H
O
HN
N
H
O
N
O
3.06
1.31
4.24
2.30
2.36
1.14
1.00
1.02
0.99
3.31
3.21
8.2 8.0 7.8 7.6 7.4 7.2 7.0 6.8 6.6 6.4 6.2 6.0 5.8 5.6 5.4 5.2 5.0 4.8 4.6 4.4 4.2 4.0 3.8 3.6 3.4 3.2 3.0 2.8 2.6 2.4 2.2 2.0
f1 (ppm)

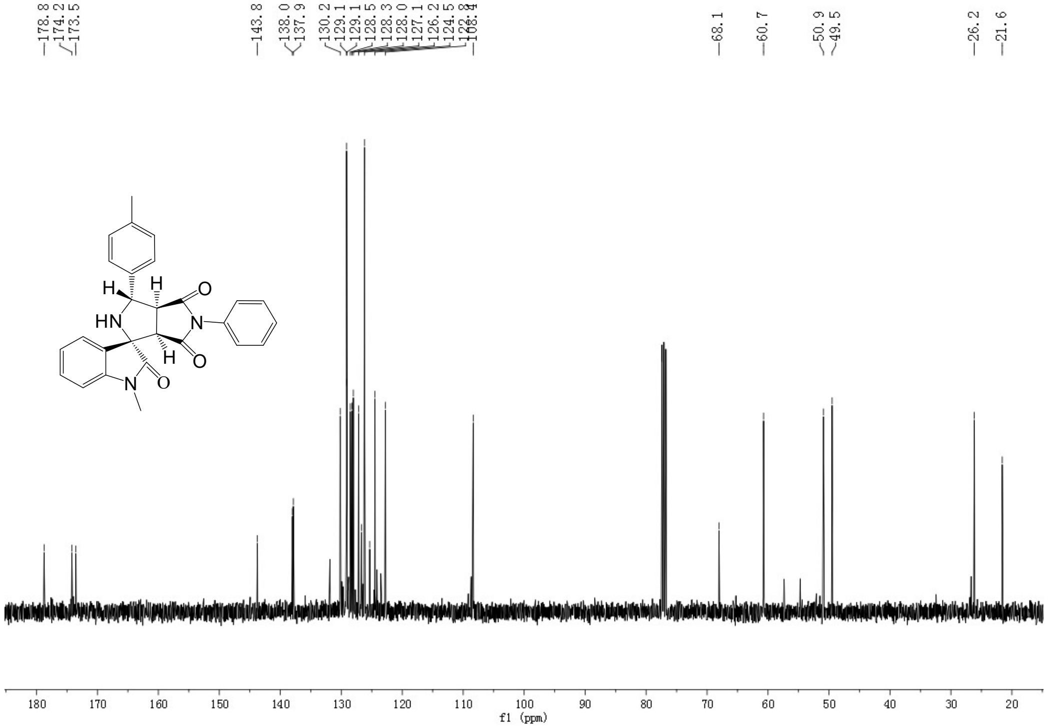
178.8
174.2
173.5
143.8
138.0
137.9
130.2
129.1
129.1
128.5
128.3
128.0
127.1
126.2
124.5
122.8
108.4
68.1
60.7
50.9
49.5
26.2
21.6
H
H
O
HN
N
H
O
N
O
180 170 160 150 140 130 120 110 100 90 80 70 60 50 40 30 20
f1 (ppm)

4p

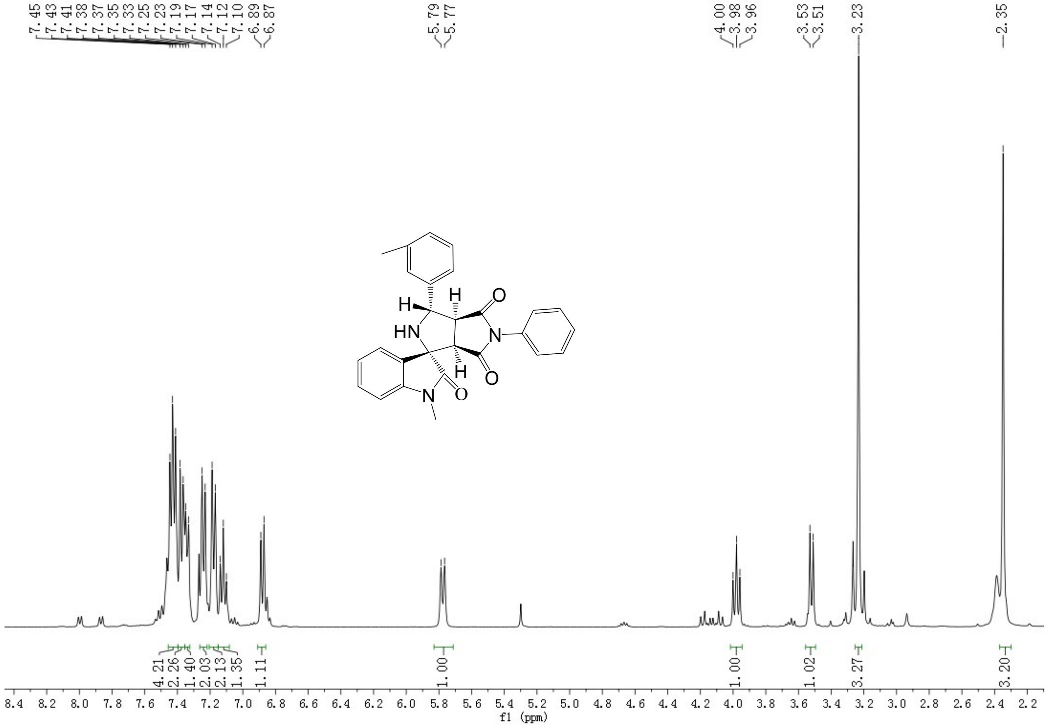
7.45
7.43
7.41
7.38
7.37
7.35
7.33
7.25
7.23
7.19
7.17
7.14
7.12
7.10
6.89
6.87
5.79
5.77
4.00
3.98
3.96
3.53
3.51
3.23
2.35
H
HN
H
O
N
H
O
N
O
4.21
2.26
1.40
2.03
2.13
1.35
1.11
1.00
1.00
1.02
3.27
3.20
8.4 8.2 8.0 7.8 7.6 7.4 7.2 7.0 6.8 6.6 6.4 6.2 6.0 5.8 5.6 5.4 5.2 5.0 4.8 4.6 4.4 4.2 4.0 3.8 3.6 3.4 3.2 3.0 2.8 2.6 2.4 2.2
f1 (ppm)

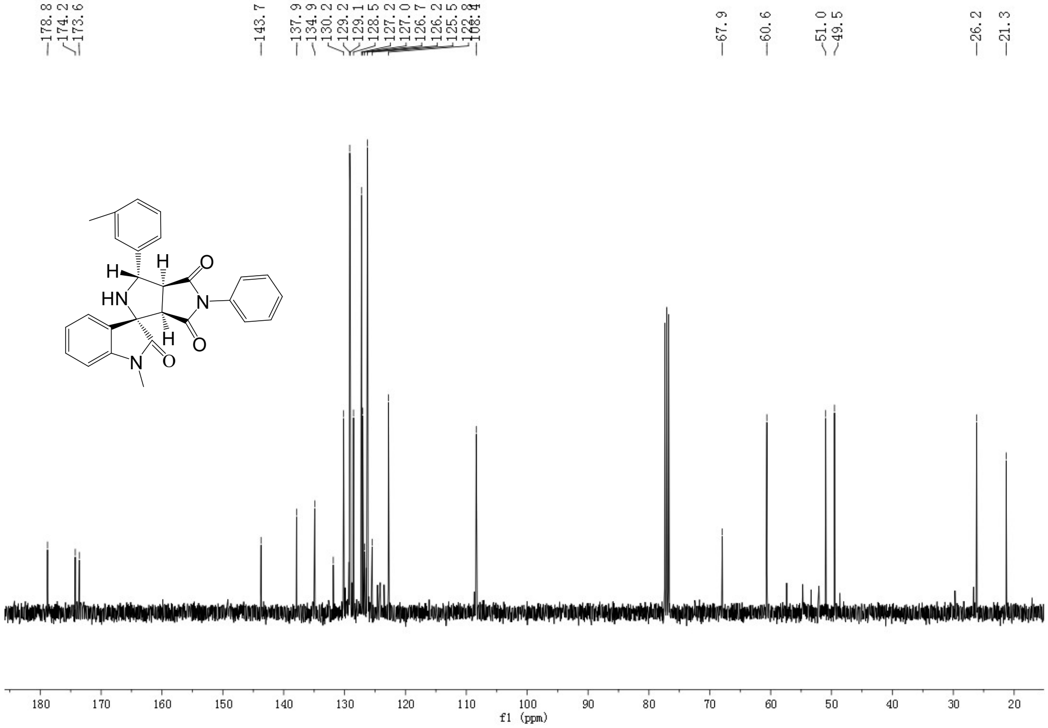
178.8
174.2
173.6
143.7
137.9
134.9
130.2
129.2
129.1
128.5
127.2
127.0
126.7
126.2
125.5
67.9
60.6
51.0
49.5
26.2
21.3
H
HN
H
O
N
H
O
N
O
180 170 160 150 140 130 120 110 100 90 80 70 60 50 40 30 20
f1 (ppm)

4q

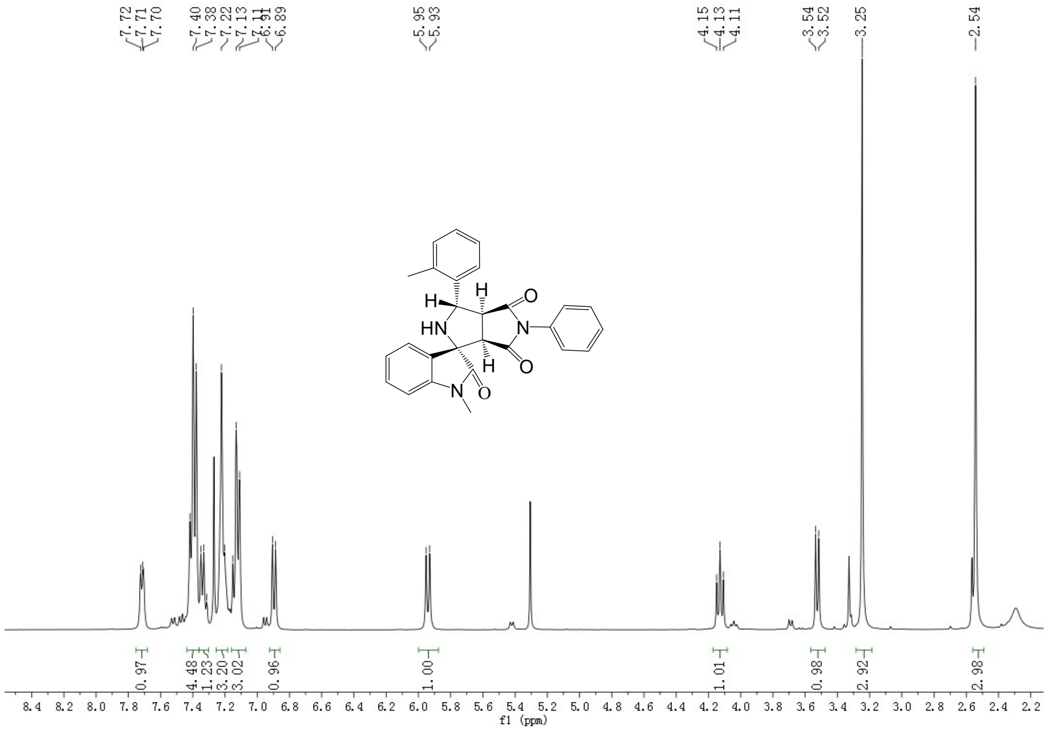
7.72
7.71
7.70
7.40
7.38
7.22
7.13
7.11
6.91
6.89
5.95
5.93
4.15
4.13
4.11
3.54
3.52
3.25
2.54
0.97
4.48
1.23
3.20
3.02
0.96
1.00
1.01
0.98
2.92
2.98
f1 (ppm)

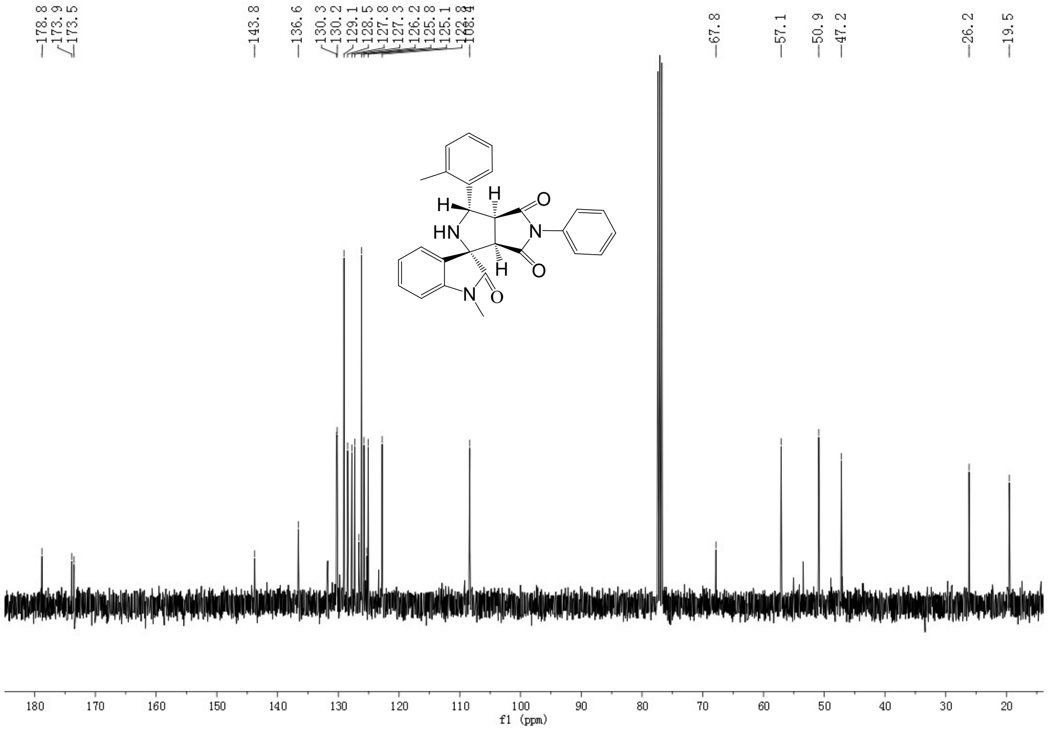
178.8
173.9
173.5
143.8
136.6
130.3
130.2
129.1
128.5
127.8
127.3
126.2
125.8
125.1
67.8
57.1
50.9
47.2
26.2
19.5
f1 (ppm)

4r

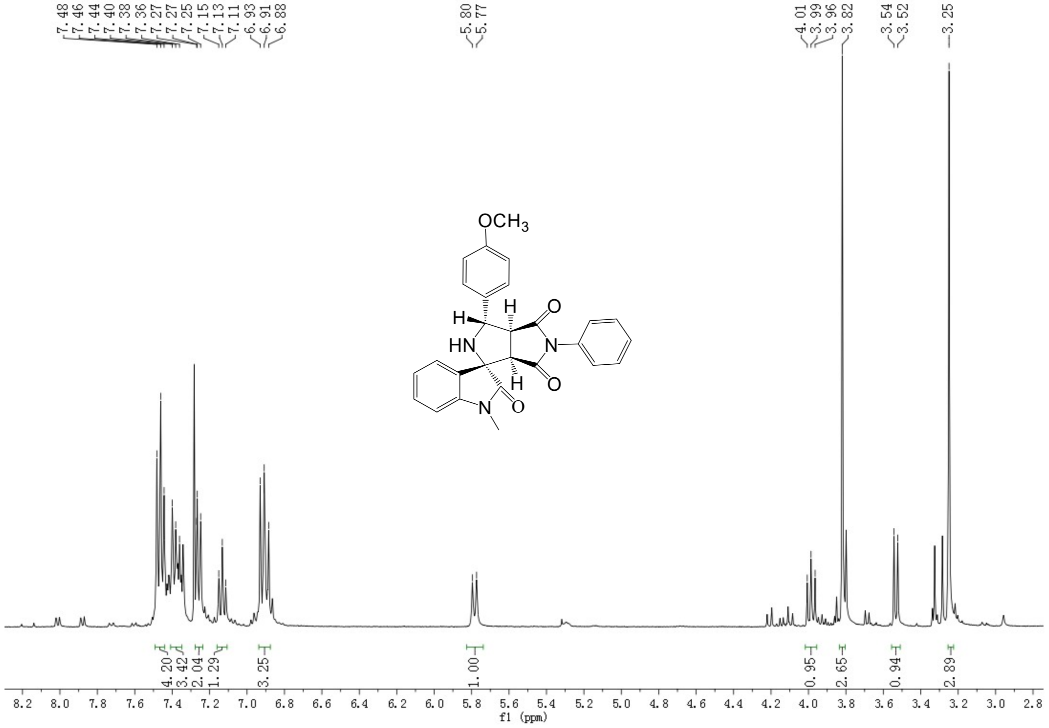

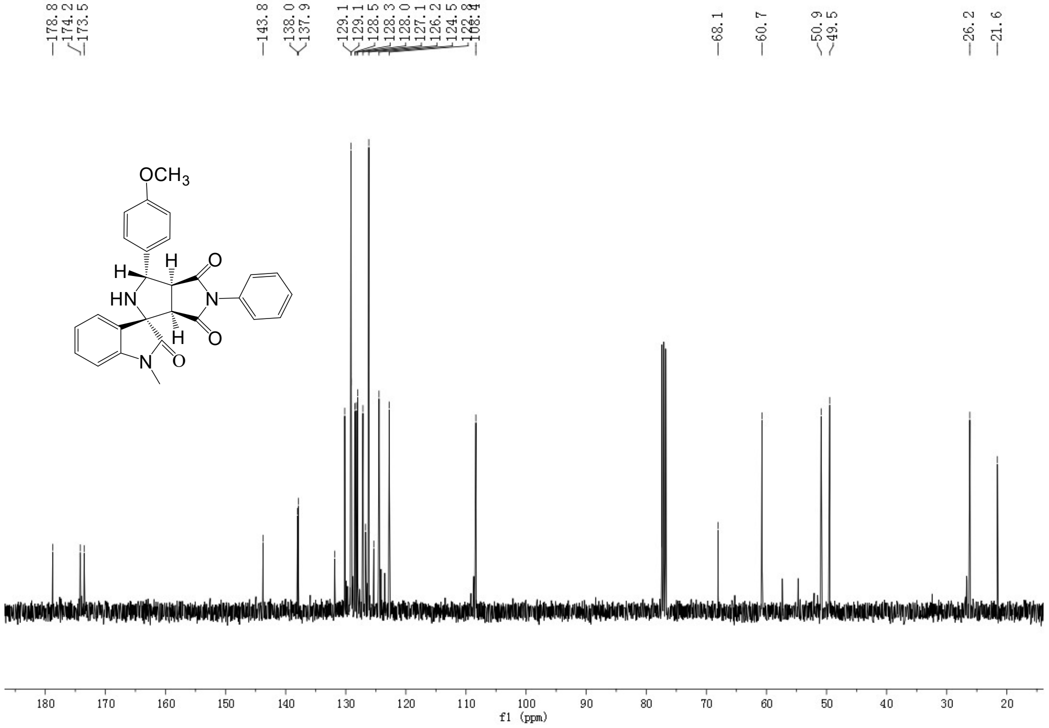

4s

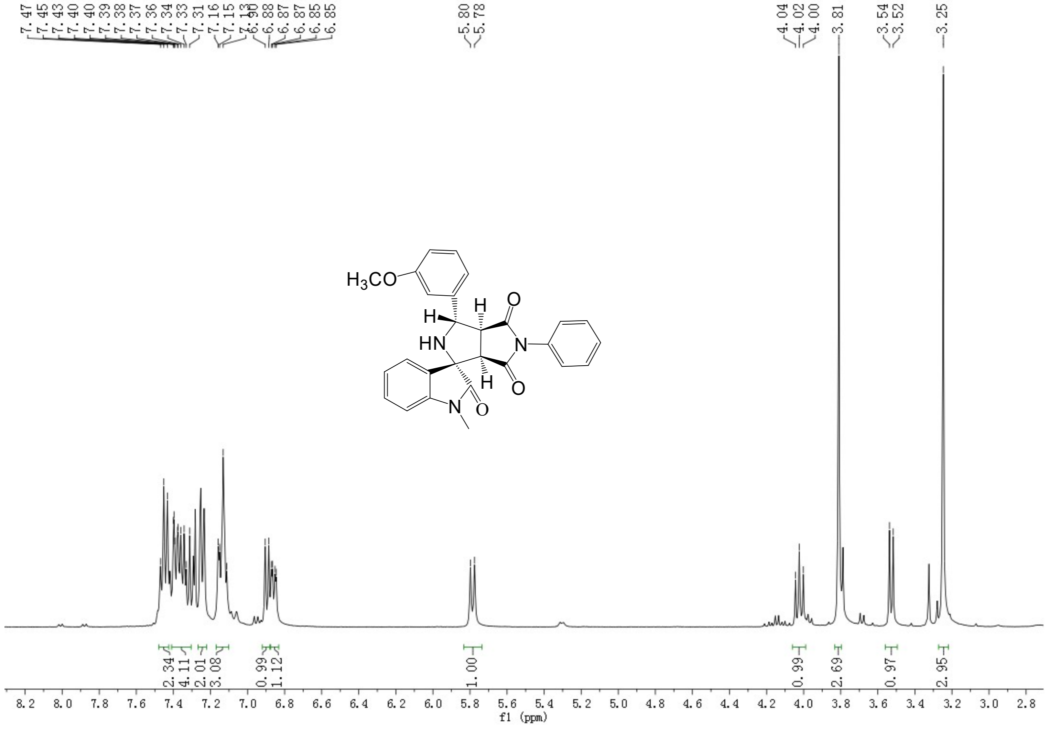

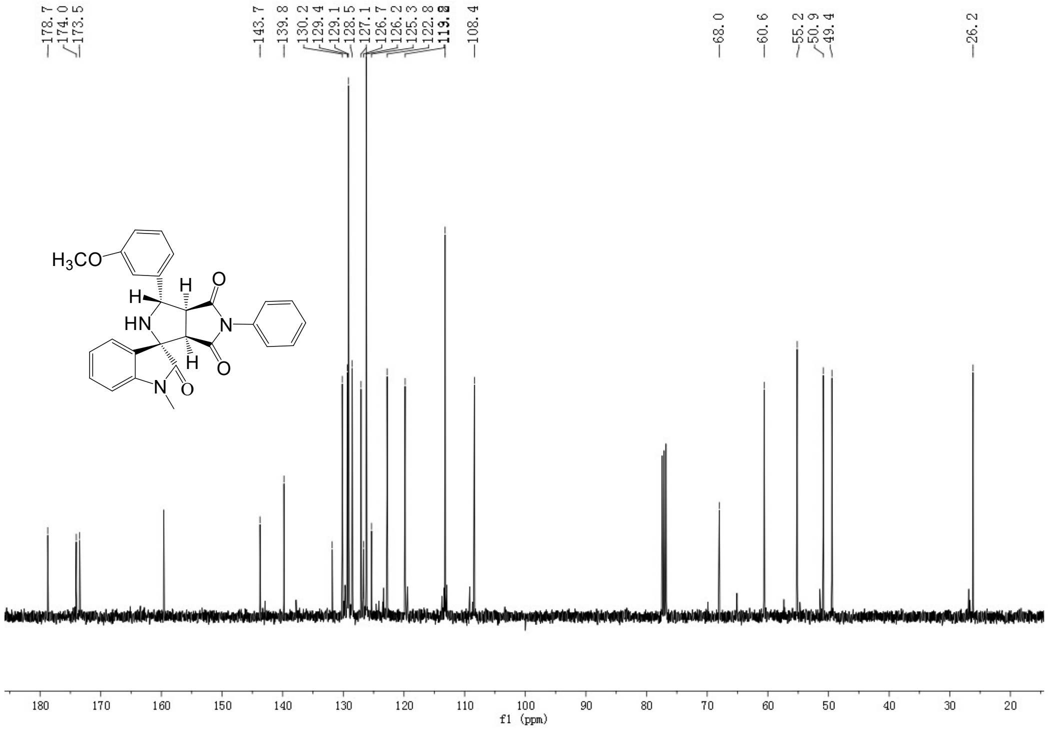

4t

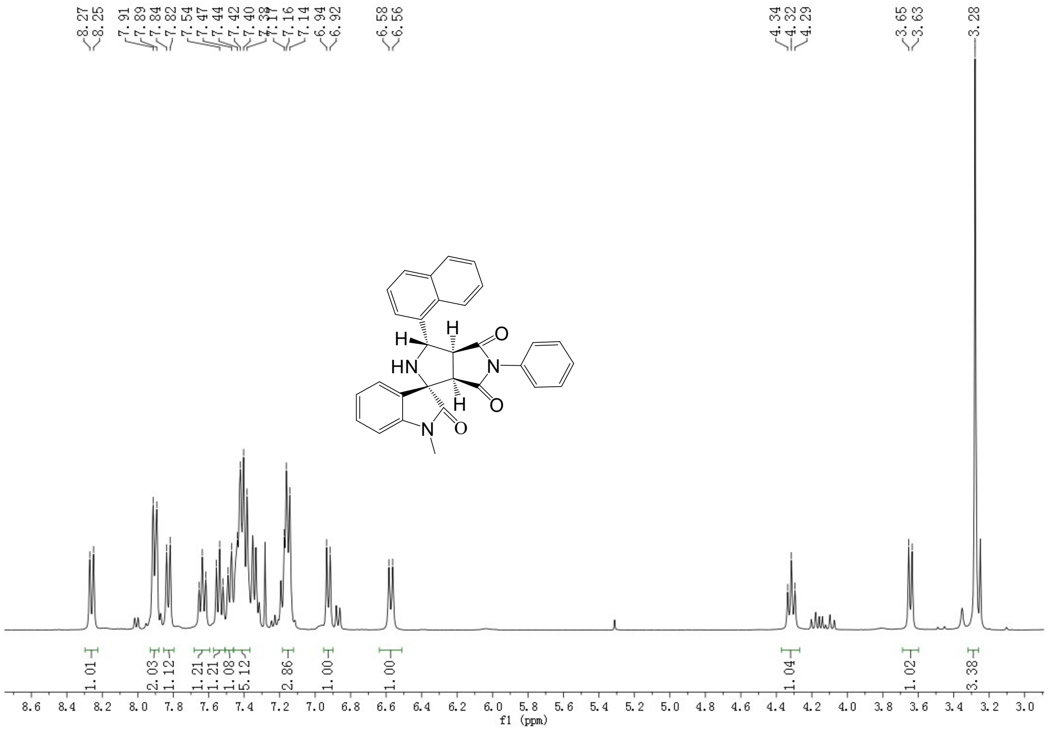

8.27
8.25
7.91
7.89
7.84
7.82
7.54
7.47
7.44
7.42
7.40
7.38
7.17
7.16
7.14
6.94
6.92
6.58
6.56
4.34
4.32
4.29
3.65
3.63
3.28
HN
H
O
N
1.01
2.03
1.12
1.21
1.21
1.08
5.12
2.86
1.00
1.00
1.04
1.02
3.38
f1 (ppm)

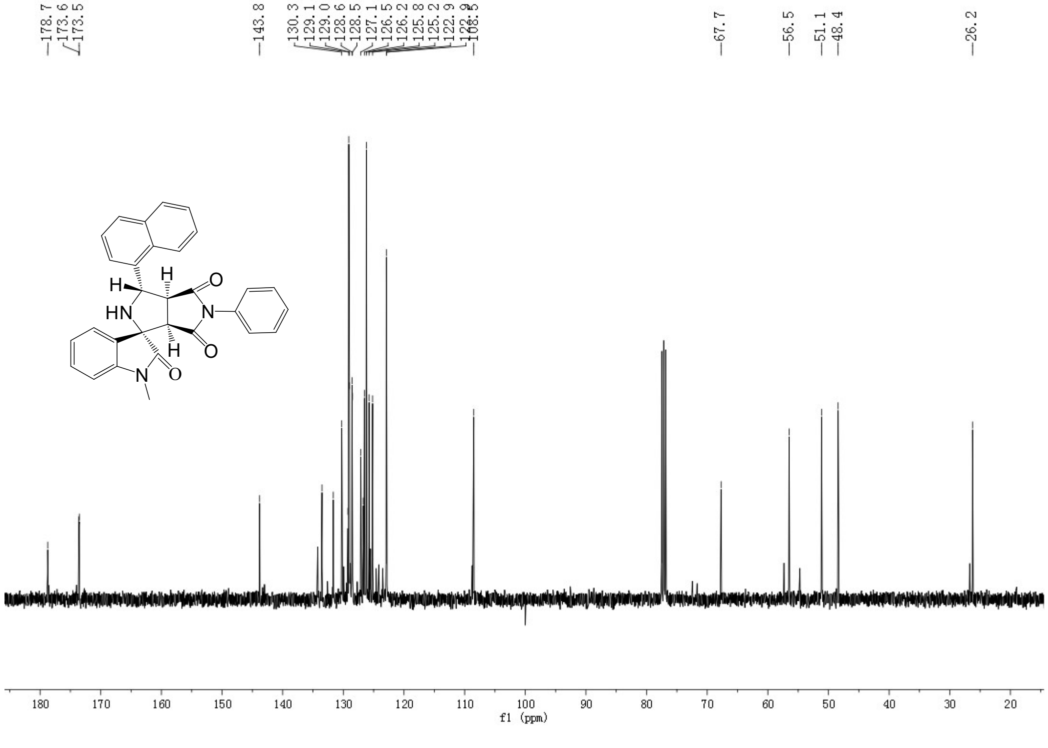

178.7
173.6
173.5
143.8
130.3
129.1
129.0
128.6
128.5
127.1
126.5
126.2
125.8
125.2
122.9
108.5
67.7
56.5
51.1
48.4
26.2
HN
H
O
N
f1 (ppm)

4u

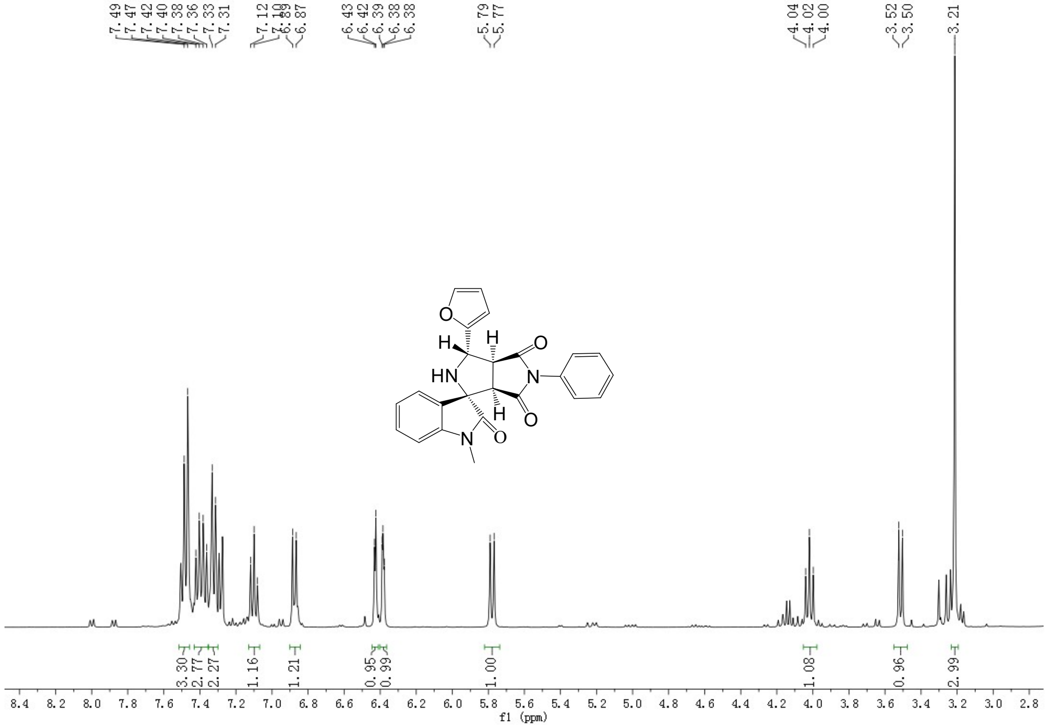

7.49
7.47
7.42
7.40
7.38
7.36
7.33
7.31
7.12
7.10
6.87
6.43
6.42
6.39
6.38
6.38
5.79
5.77
4.04
4.02
4.00
3.52
3.50
3.21
HN
O
N
3.30
2.77
2.27
1.16
1.21
0.95
0.99
1.00
1.08
0.96
2.99
f1 (ppm)

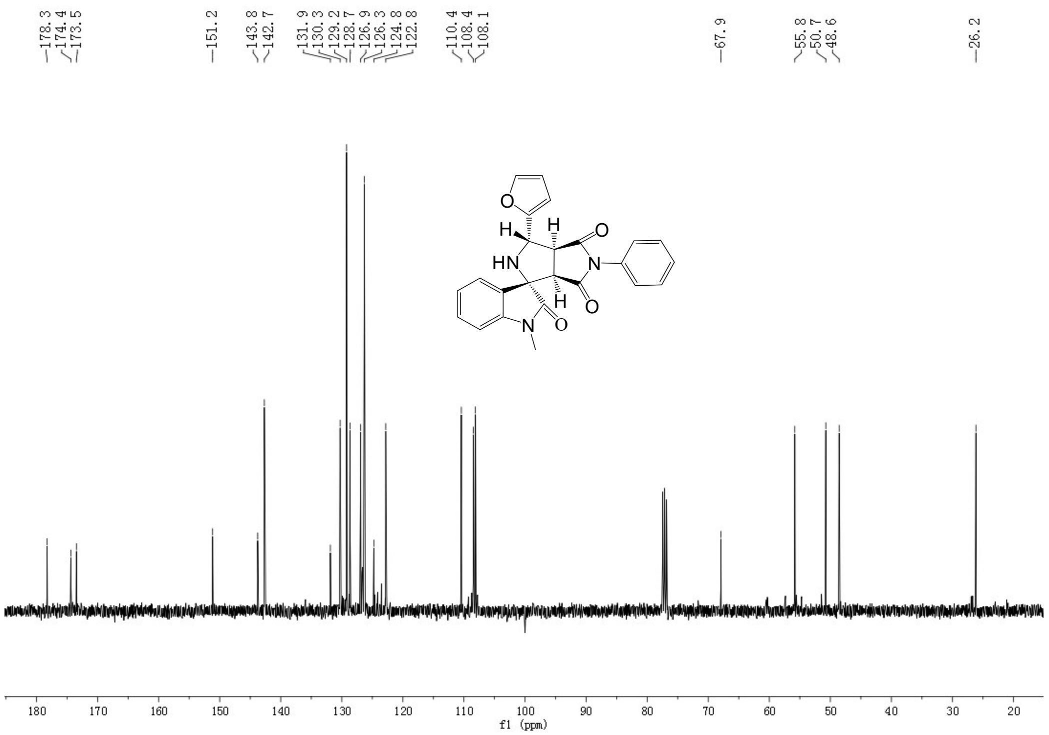

178.3
174.4
173.5
151.2
143.8
142.7
131.9
130.3
129.2
128.7
126.9
126.3
124.8
122.8
110.4
108.4
108.1
67.9
55.8
50.7
48.6
26.2
HN
O
N
f1 (ppm)

4v

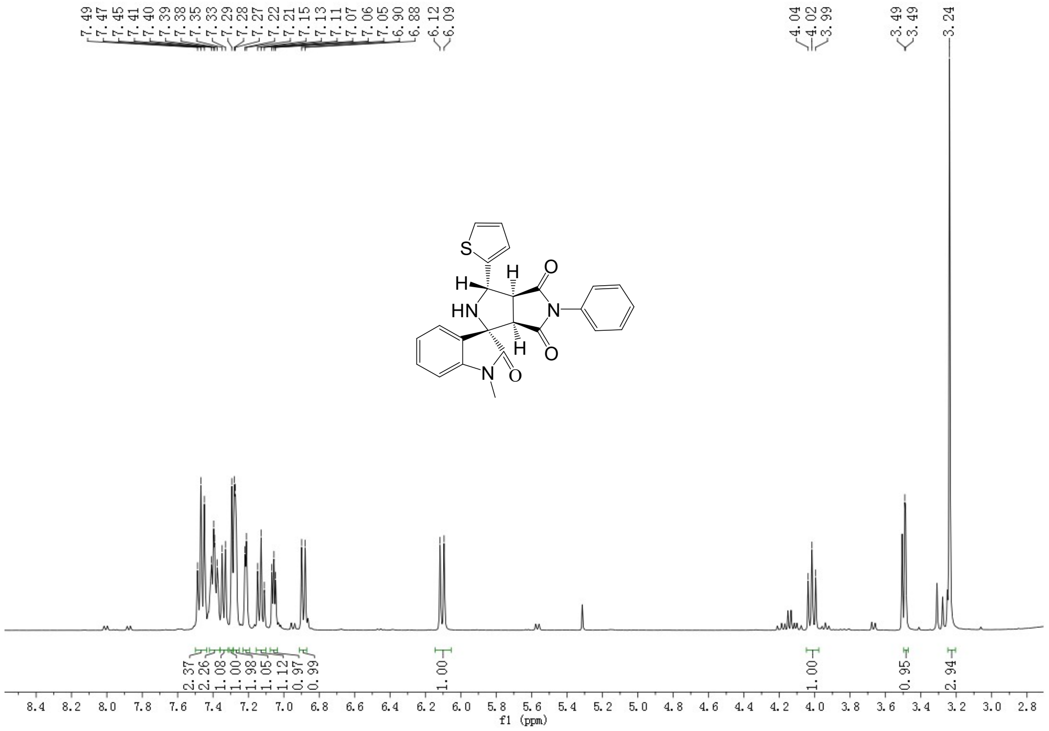
7.49
7.47
7.45
7.41
7.40
7.39
7.38
7.35
7.33
7.29
7.28
7.27
7.22
7.21
7.15
7.13
7.11
7.07
7.06
7.05
6.90
6.88
6.12
6.09
4.04
4.02
3.99
3.49
3.49
3.24
S
H
HN
H
O
N
H
O
N
O
2.37
2.26
1.08
1.00
1.98
1.05
1.12
0.97
0.99
1.00
1.00
0.95
2.94
8.4 8.2 8.0 7.8 7.6 7.4 7.2 7.0 6.8 6.6 6.4 6.2 6.0 5.8 5.6 5.4 5.2 5.0 4.8 4.6 4.4 4.2 4.0 3.8 3.6 3.4 3.2 3.0 2.8
f1 (ppm)

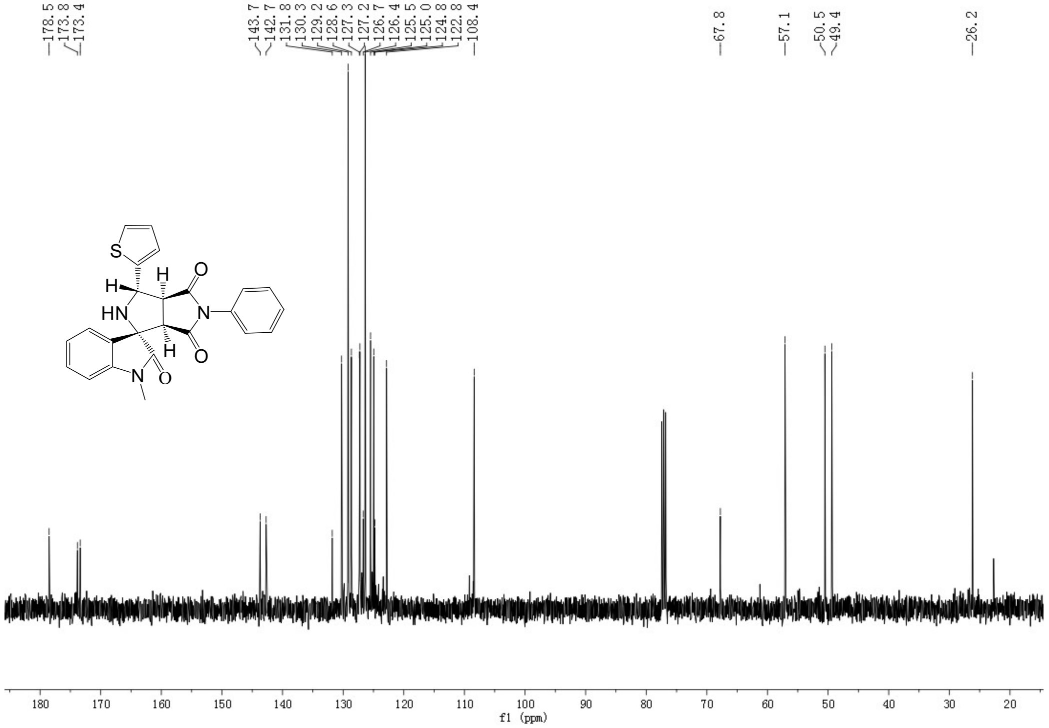
178.5
173.8
173.4
143.7
142.7
131.8
130.3
129.2
128.6
127.3
127.2
126.7
126.4
125.5
125.0
124.8
122.8
108.4
67.8
57.1
50.5
49.4
26.2
S
H
HN
H
O
N
H
O
N
O
180 170 160 150 140 130 120 110 100 90 80 70 60 50 40 30 20
f1 (ppm)

4w

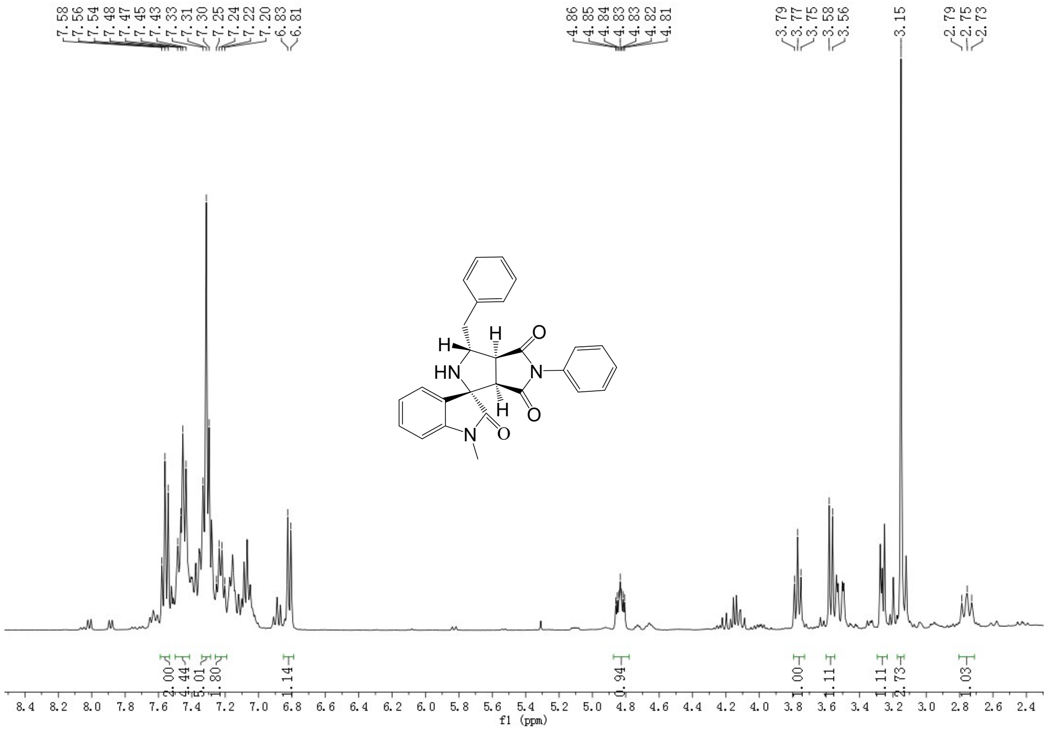
7.58
7.56
7.54
7.48
7.47
7.45
7.43
7.33
7.31
7.30
7.25
7.24
7.22
7.20
6.83
6.81
4.86
4.85
4.84
4.83
4.83
4.82
4.81
3.79
3.77
3.75
3.58
3.56
3.15
2.79
2.75
2.73
H
H
O
HN
N
H
O
N
O
2.00
4.44
5.01
1.80
1.14
0.94
1.00
1.11
1.11
2.73
1.03
f1 (ppm)

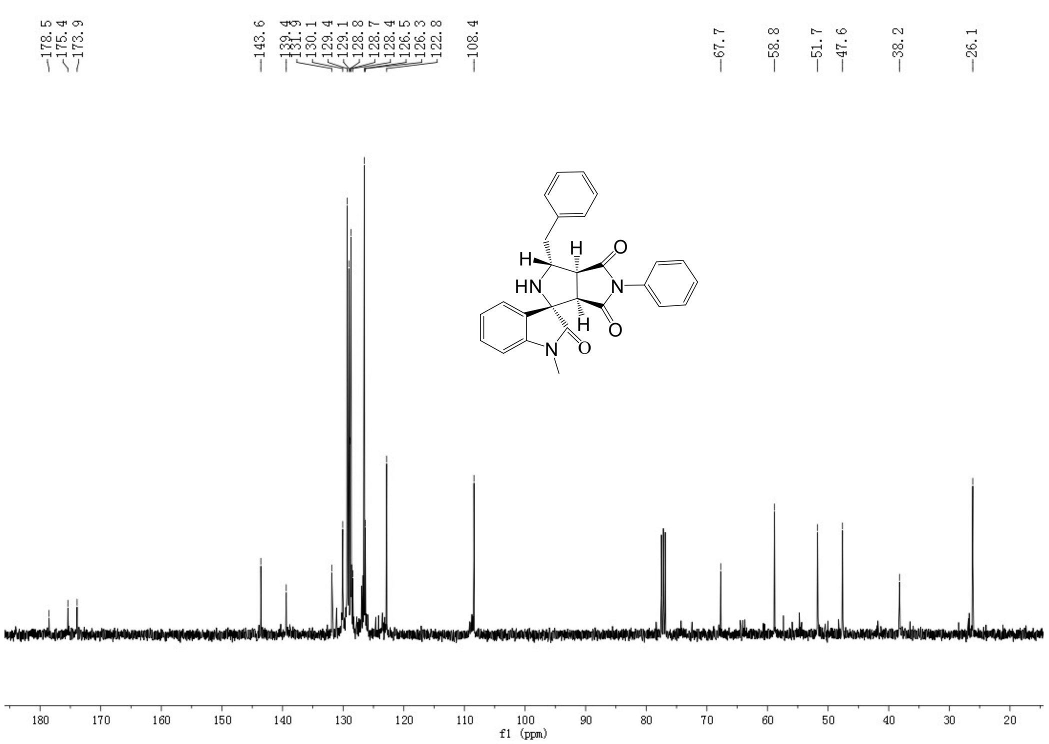
178.5
175.4
173.9
143.6
139.4
131.9
130.1
129.4
129.1
128.8
128.7
128.4
126.5
126.3
122.8
108.4
67.7
58.8
51.7
47.6
38.2
26.1
H
H
O
HN
N
H
O
N
O
f1 (ppm)

4x

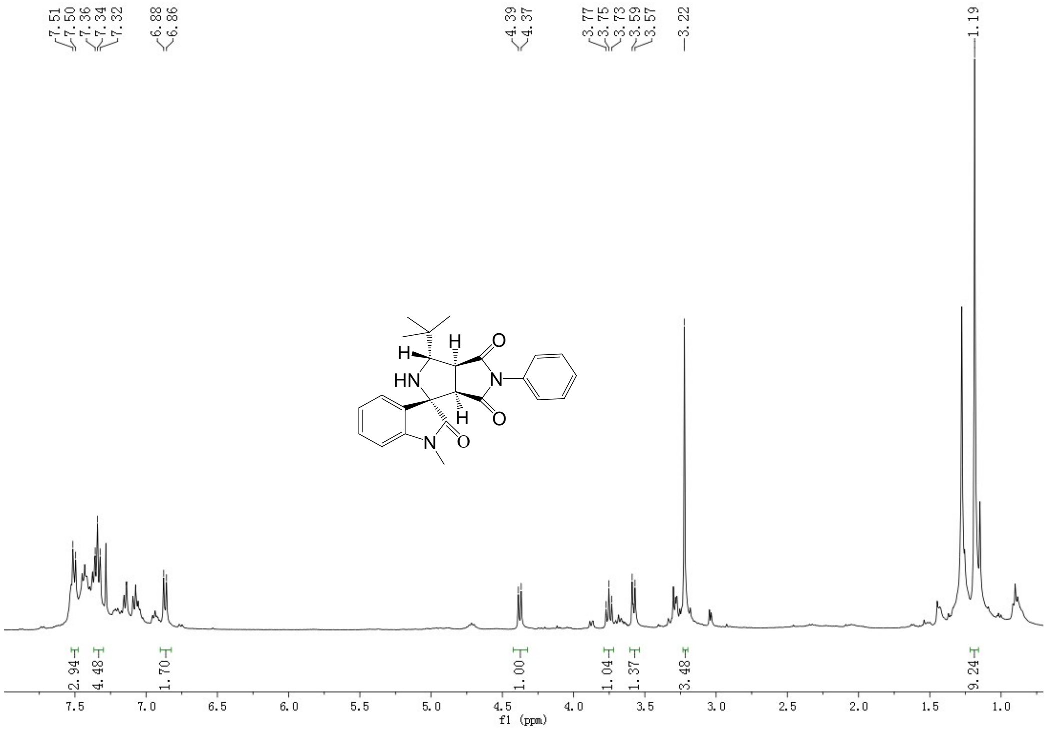
7.51
7.50
7.36
7.34
7.32
6.88
6.86
4.39
4.37
3.77
3.75
3.73
3.59
3.57
3.22
1.19
H
H
HN
N
O
O
N
O
H
2.94
4.48
1.70
1.00
1.04
1.37
3.48
9.24
7.5 7.0 6.5 6.0 5.5 5.0 4.5 4.0 3.5 3.0 2.5 2.0 1.5 1.0
f1 (ppm)

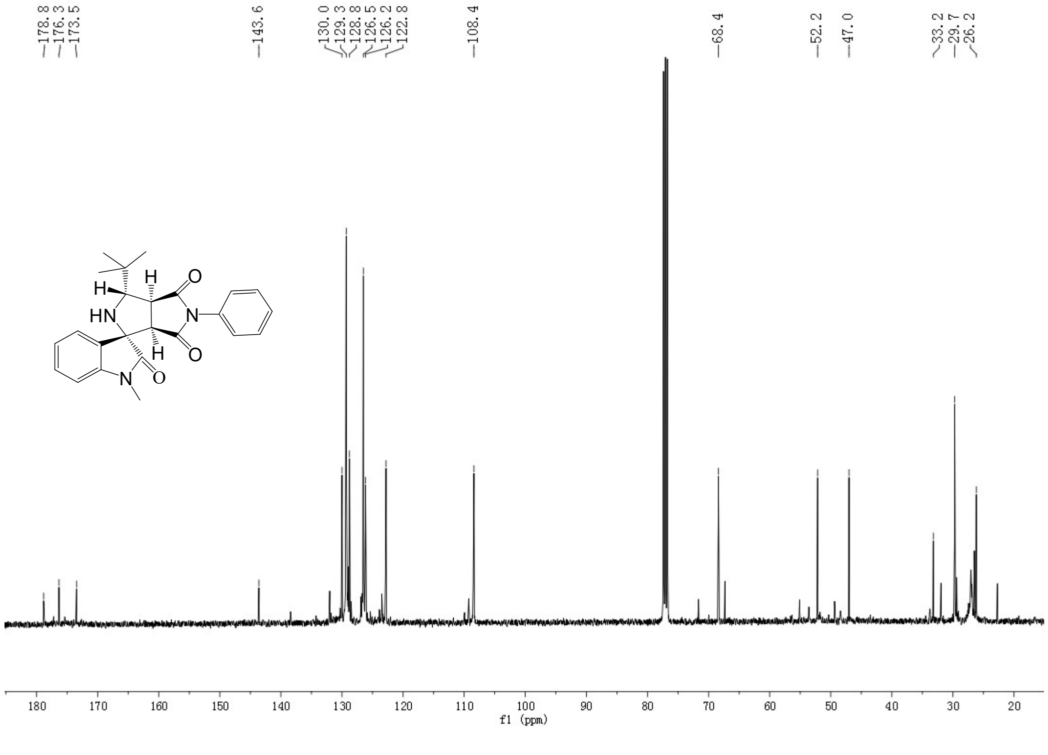
178.8
176.3
173.5
143.6
130.0
129.3
128.8
126.5
126.2
122.8
108.4
68.4
52.2
47.0
33.2
29.7
26.2
H
H
HN
N
O
O
N
O
H
180 170 160 150 140 130 120 110 100 90 80 70 60 50 40 30 20
f1 (ppm)

5a

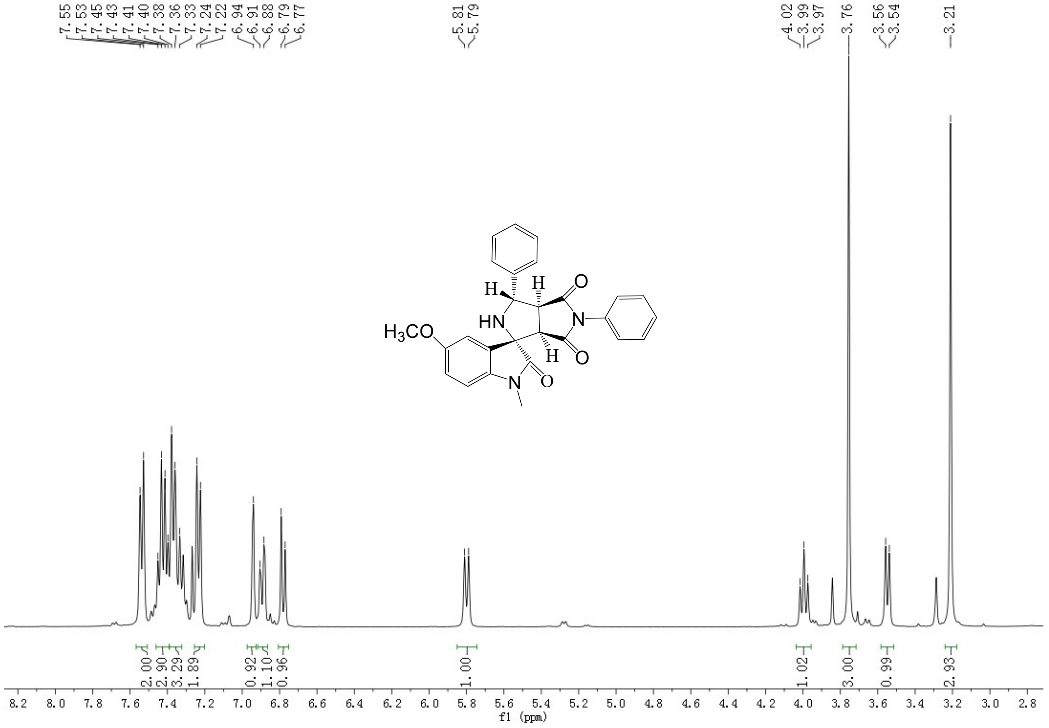
7.55
7.53
7.45
7.43
7.41
7.40
7.38
7.36
7.33
7.24
7.22
6.94
6.91
6.88
6.79
6.77
5.81
5.79
4.02
3.99
3.97
3.76
3.56
3.54
3.21
H
H
HN
N
O
O
O
H_3CO
H
N
2.00
2.90
3.29
1.89
0.92
1.10
0.96
1.00
1.02
3.00
0.99
2.93
f1 (ppm)

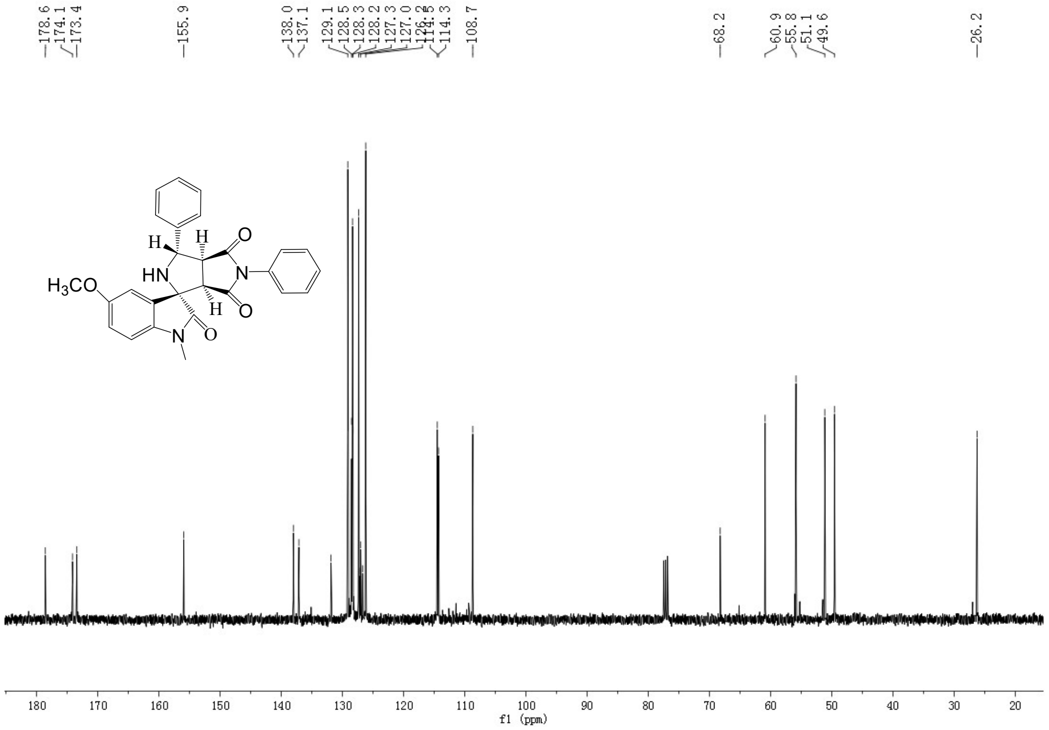
178.6
174.1
173.4
155.9
138.0
137.1
129.1
128.5
128.3
128.2
127.3
127.0
126.2
114.5
114.3
108.7
68.2
60.9
55.8
51.1
49.6
26.2
H
H
HN
N
O
O
O
H_3CO
H
N
f1 (ppm)

5b

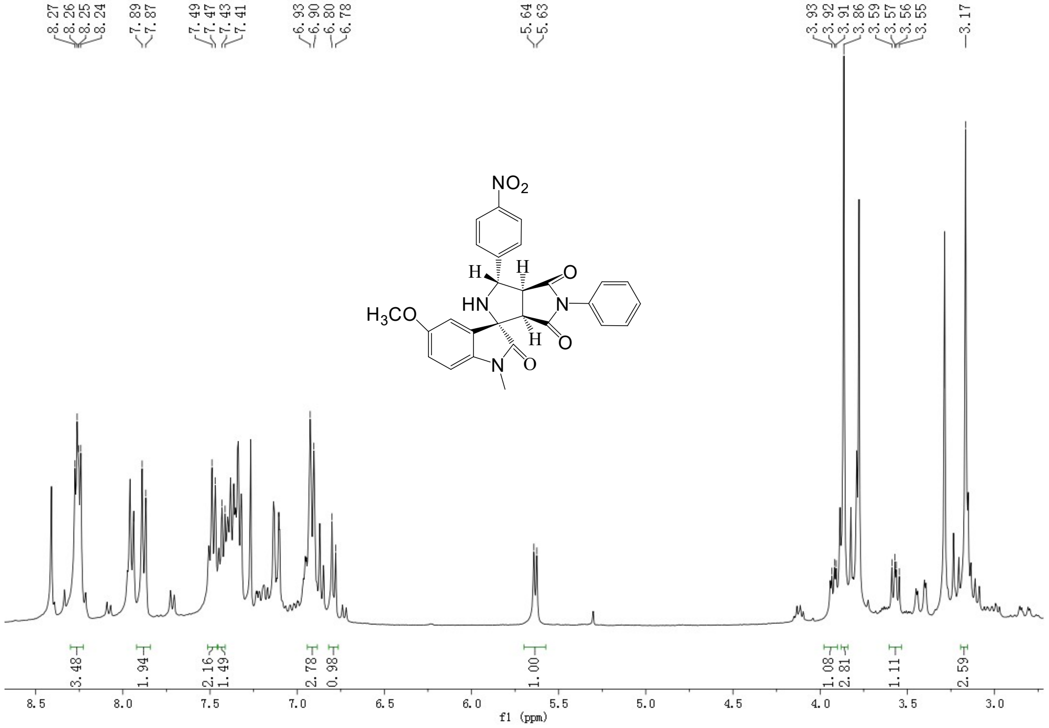
8.27
8.26
8.25
8.24
7.89
7.87
7.49
7.47
7.43
7.41
6.93
6.90
6.80
6.78
5.64
5.63
3.93
3.92
3.91
3.86
3.59
3.57
3.56
3.55
3.17
NO2
H
H
O
HN
N
H3CO
H
O
N
O
3.48
1.94
2.16
1.49
2.78
0.98
1.00
1.08
2.81
1.11
2.59
8.5
8.0
7.5
7.0
6.5
6.0
5.5
5.0
4.5
4.0
3.5
3.0
f1 (ppm)

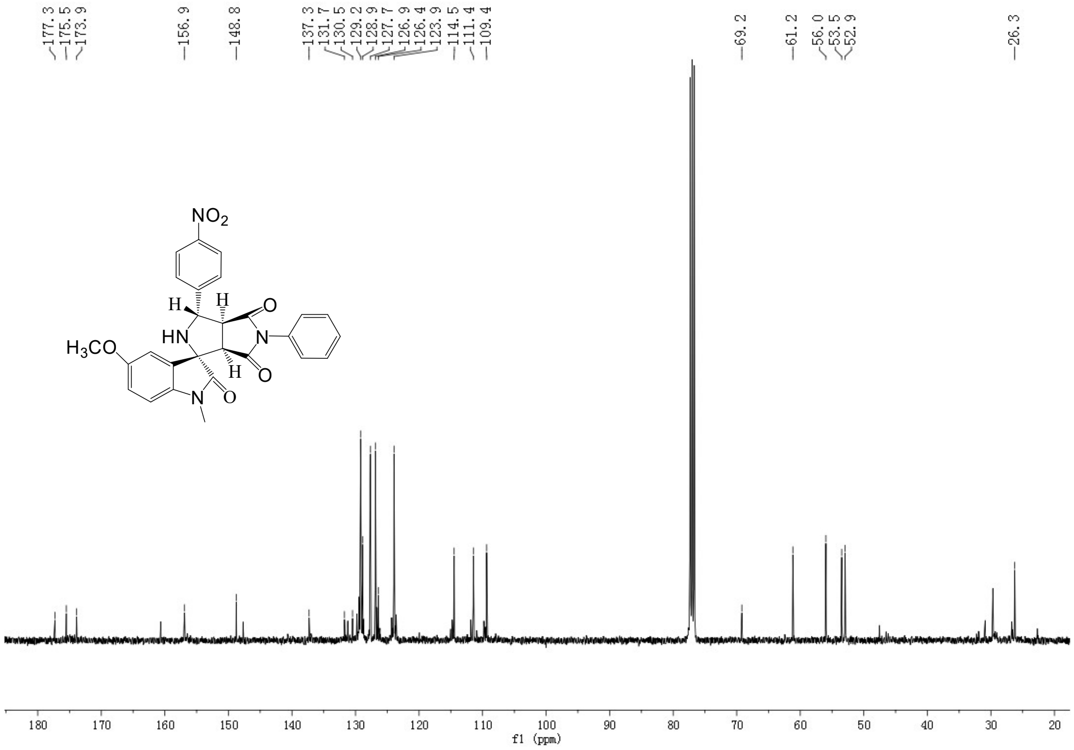
177.3
175.5
173.9
156.9
148.8
137.3
131.7
130.5
129.2
128.9
127.7
126.9
126.4
123.9
114.5
111.4
109.4
69.2
61.2
56.0
53.5
52.9
26.3
NO2
H
H
O
HN
N
H3CO
H
O
N
O
180
170
160
150
140
130
120
110
100
90
80
70
60
50
40
30
20
f1 (ppm)

5c

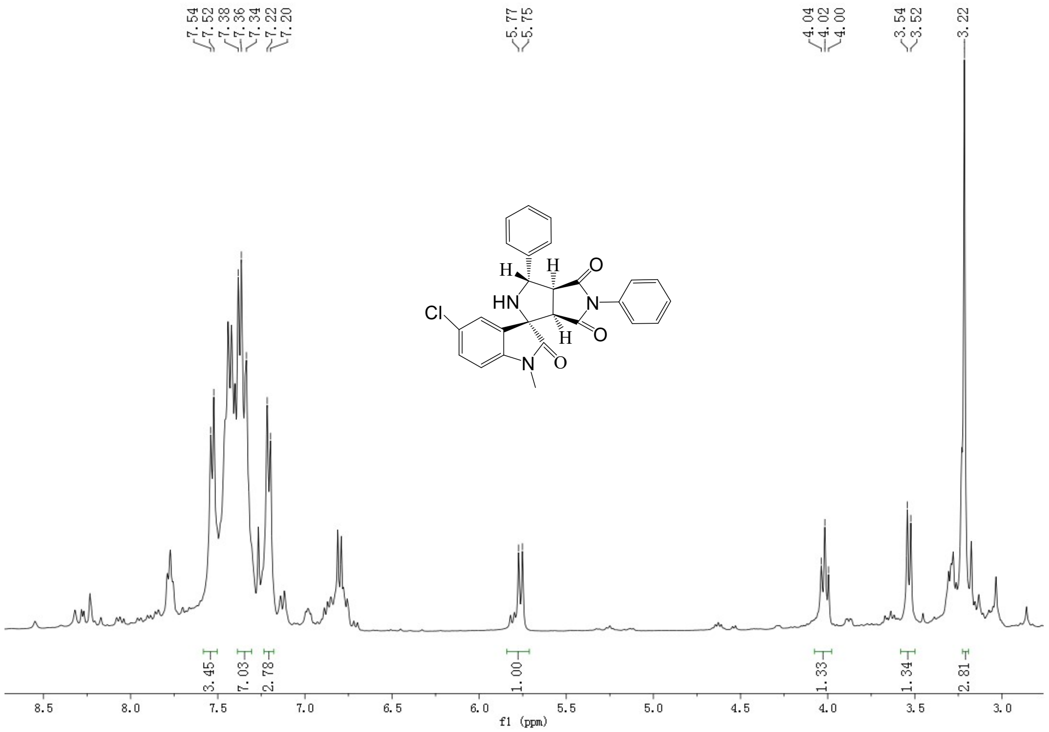
7.54
7.52
7.38
7.36
7.34
7.22
7.20
5.77
5.75
4.04
4.02
4.00
3.54
3.52
3.22
Cl
HN
H
N
O
3.45
7.03
2.78
1.00
1.33
1.34
2.81
8.5
8.0
7.5
7.0
6.5
6.0
5.5
5.0
4.5
4.0
3.5
3.0
f1 (ppm)

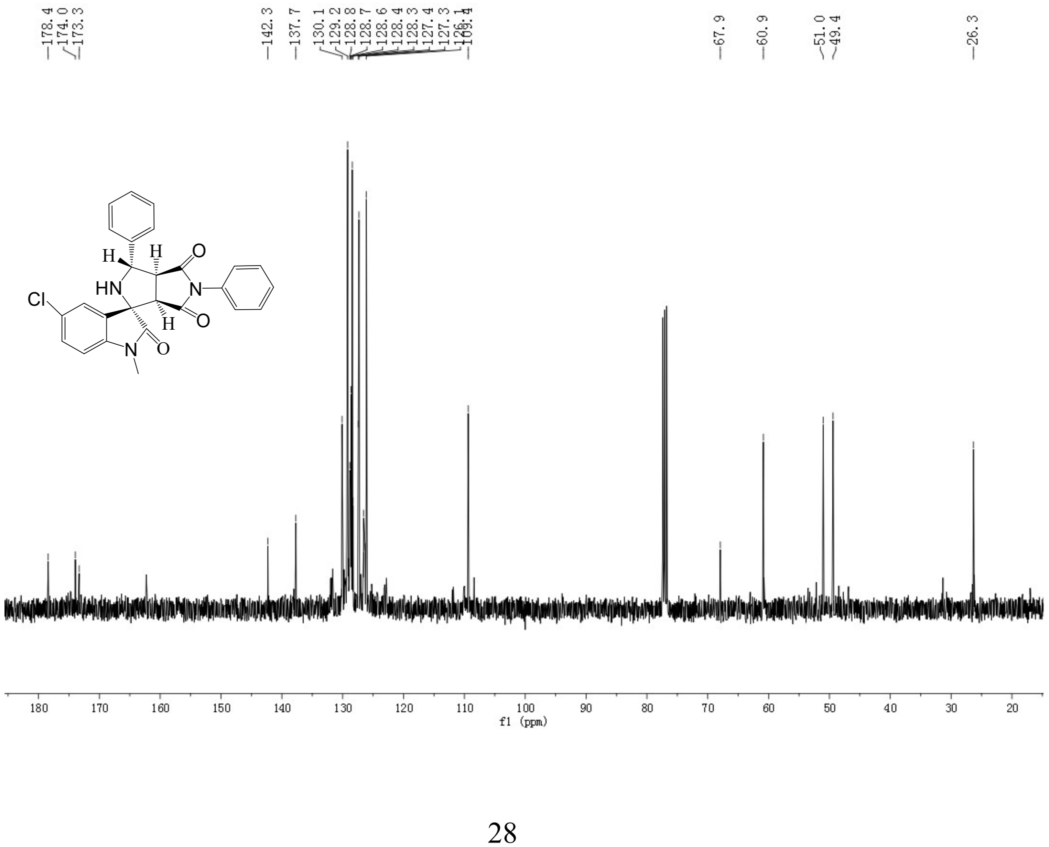
178.4
174.0
173.3
142.3
137.7
130.1
129.2
128.8
128.7
128.6
128.4
128.3
127.4
127.3
109.4
67.9
60.9
51.0
49.4
26.3
Cl
HN
H
N
O
180
170
160
150
140
130
120
110
100
90
80
70
60
50
40
30
20
f1 (ppm)

5d

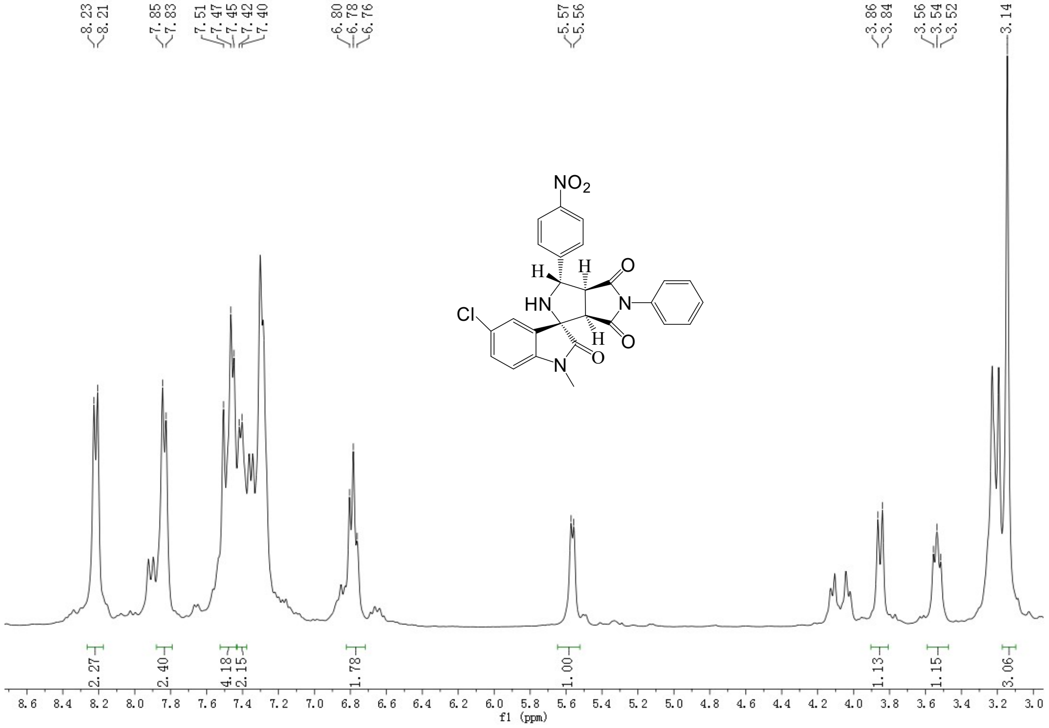
8.23
8.21
7.85
7.83
7.51
7.47
7.45
7.42
7.40
6.80
6.78
6.76
5.57
5.56
3.86
3.84
3.56
3.54
3.52
3.14
NO2
H
H
O
HN
N
Cl
H
O
O
N
2.27
2.40
4.18
2.15
1.78
1.00
1.13
1.15
3.06
f1 (ppm)

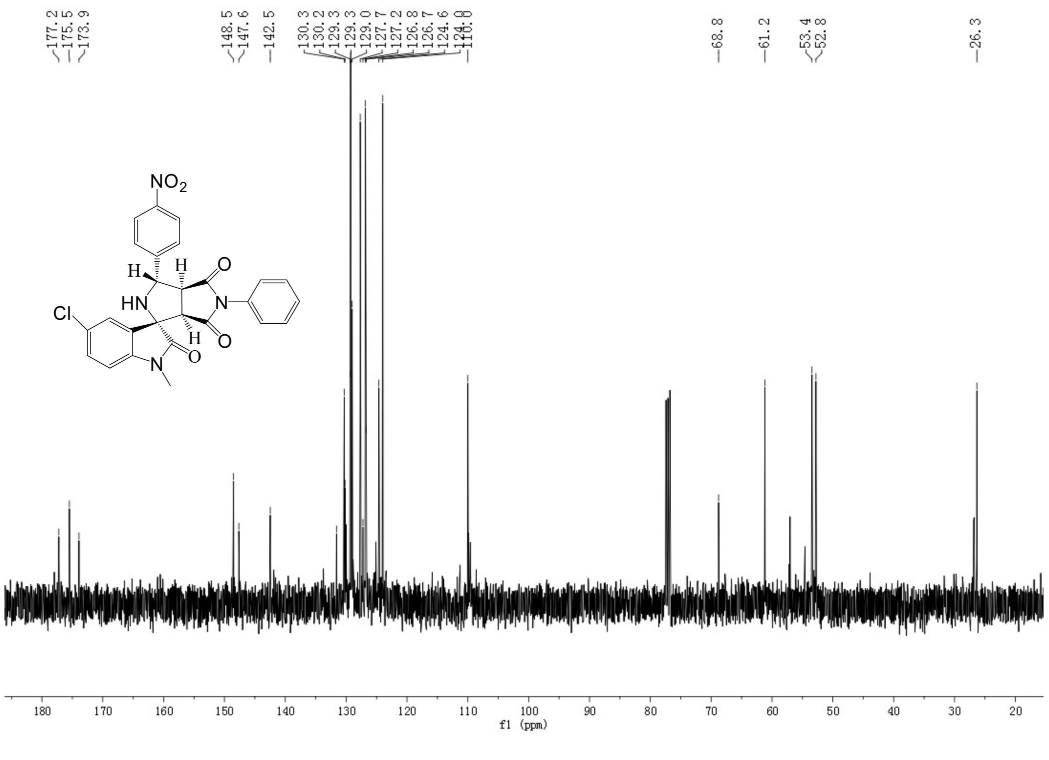
177.2
175.5
173.9
148.5
147.6
142.5
130.3
130.2
129.3
129.3
129.0
127.7
127.2
126.8
126.7
124.6
124.0
110.0
68.8
61.2
53.4
52.8
26.3
NO2
H
H
O
HN
N
Cl
H
O
O
N
f1 (ppm)

5e

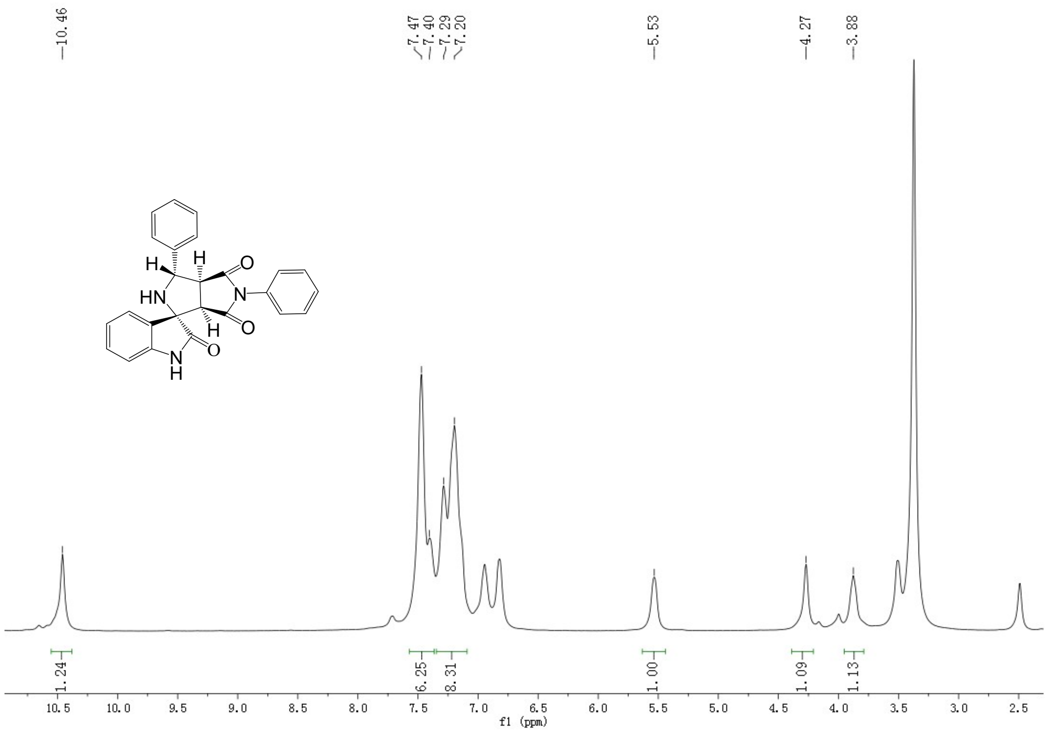
10.46
7.47
7.40
7.29
7.20
5.53
4.27
3.88
1.24
6.25
8.31
1.00
1.09
1.13
f1 (ppm)

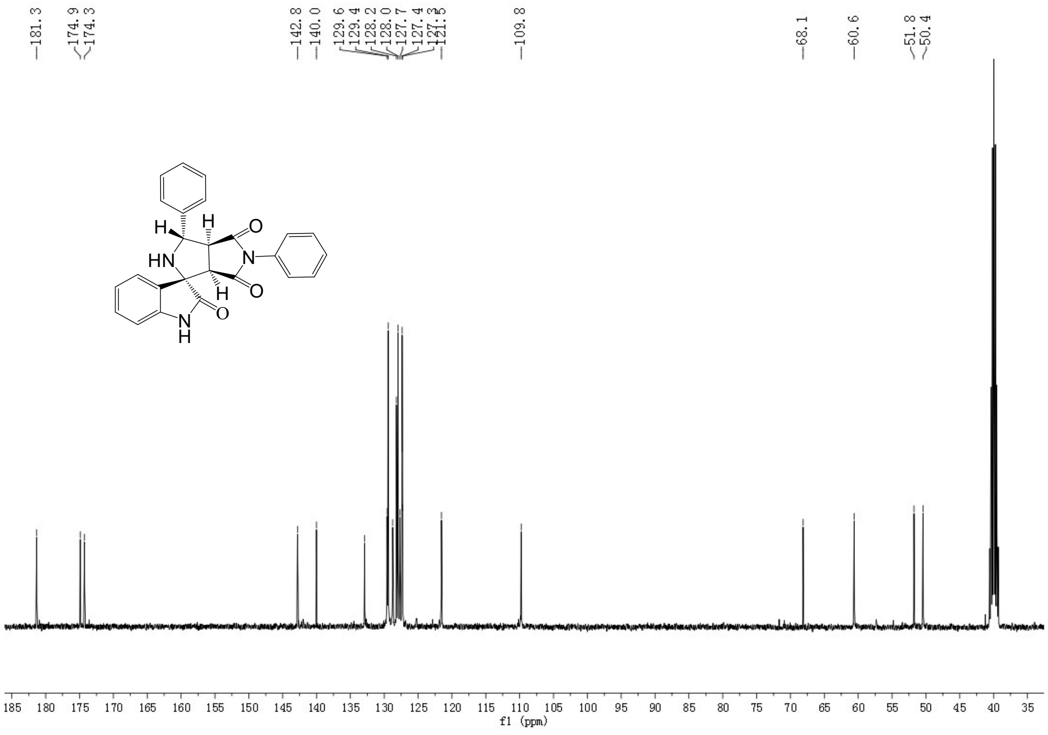
181.3
174.9
174.3
142.8
140.0
129.6
129.4
128.2
128.0
127.7
127.4
f1 (ppm)

5f

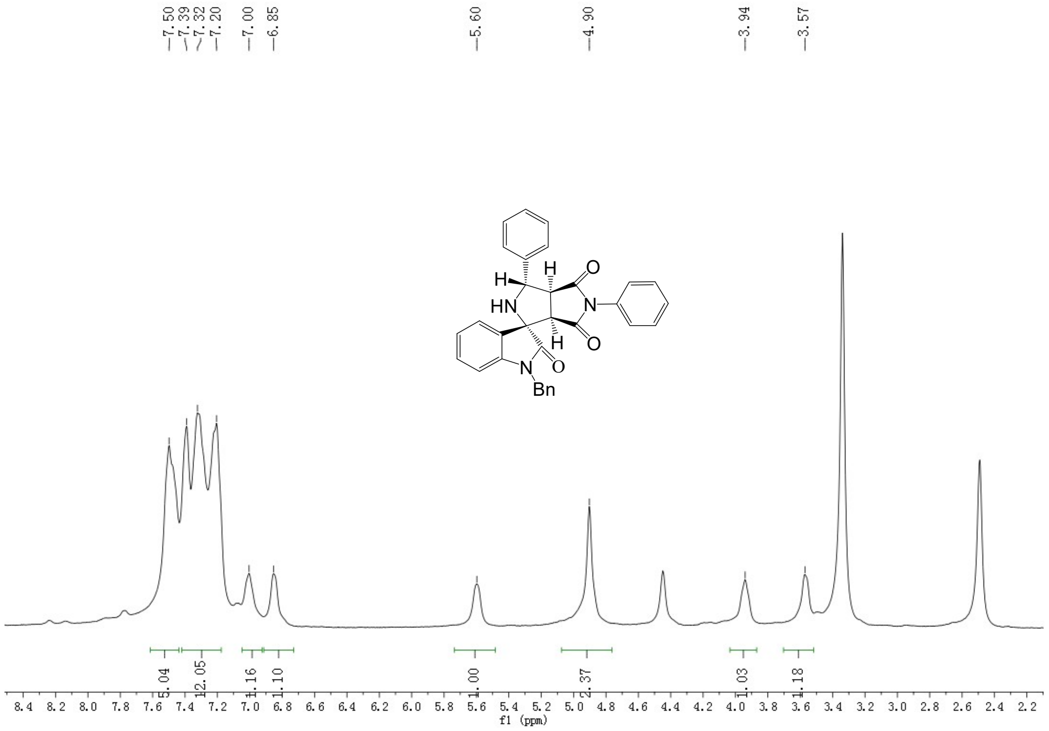
7.50
7.39
7.32
7.20
7.00
6.85
5.60
4.90
3.94
3.57
5.04
12.05
1.16
1.10
1.00
2.37
1.03
1.18
H
H
O
HN
N
H
O
N
O
Bn
f1 (ppm)

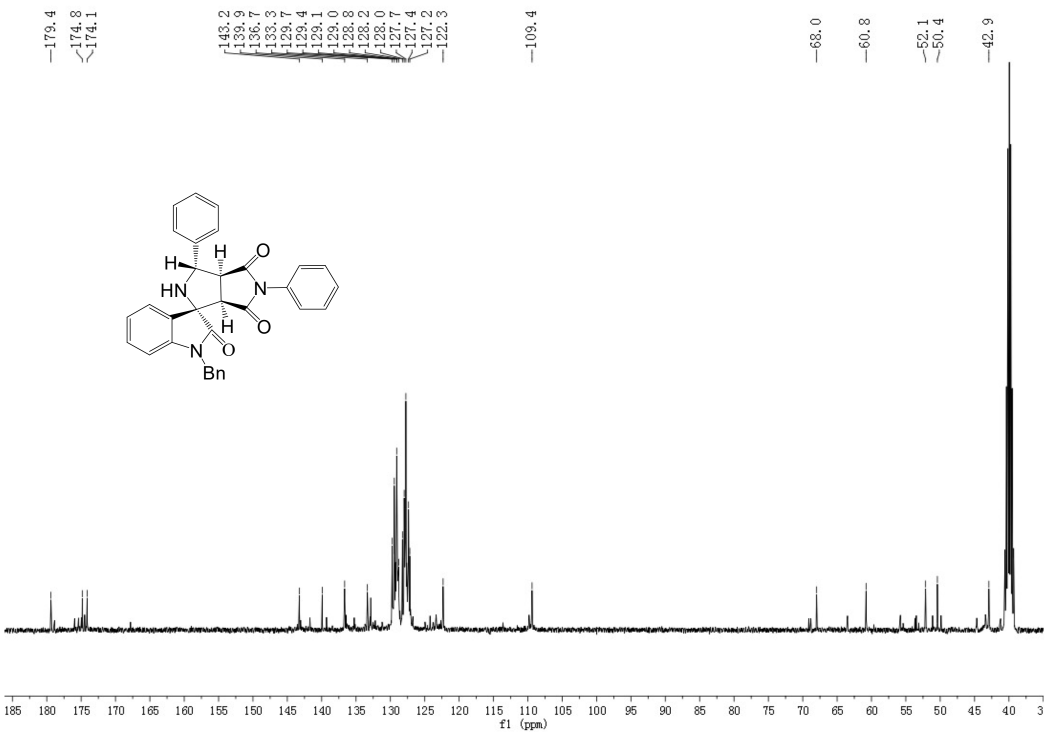
179.4
174.8
174.1
143.2
139.9
136.7
133.3
129.7
129.4
129.1
129.0
128.8
128.2
128.0
127.7
127.4
127.2
122.3
109.4
68.0
60.8
52.1
50.4
42.9
H
H
O
HN
N
H
O
N
O
Bn
f1 (ppm)

5g

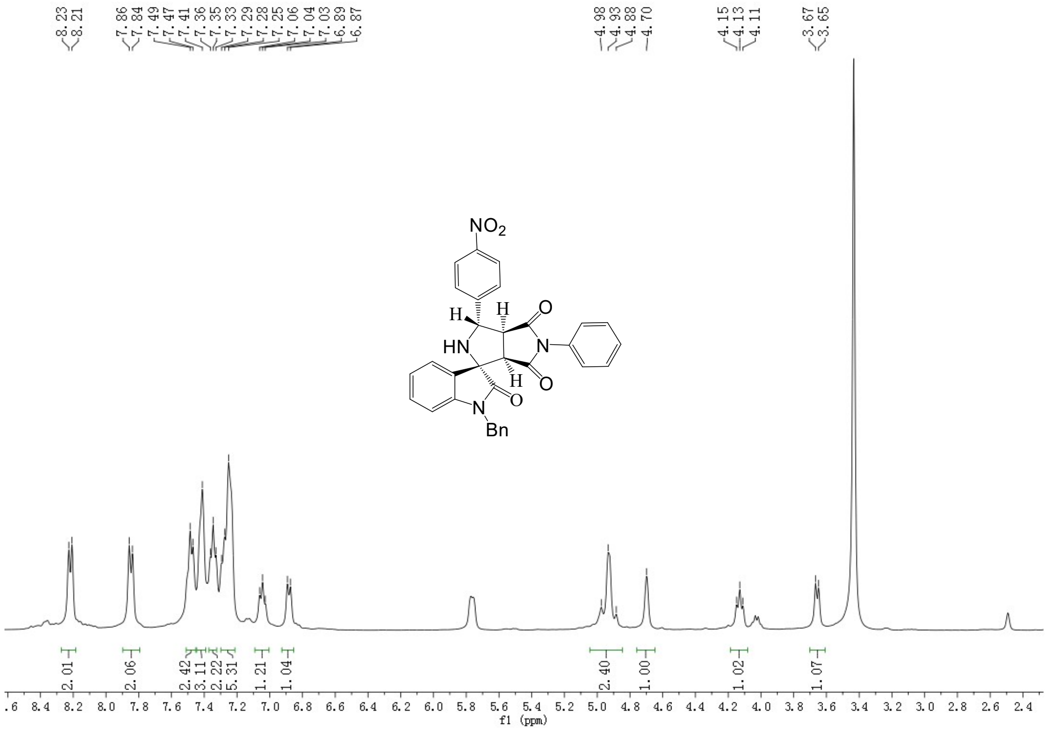
8.23
8.21
7.86
7.84
7.49
7.47
7.41
7.36
7.35
7.33
7.29
7.28
7.25
7.06
7.04
7.03
6.89
6.87
4.98
4.93
4.88
4.70
4.15
4.13
4.11
3.67
3.65
NO2
H
H
O
HN
N
H
O
N
O
Bn
2.01
2.06
2.42
3.11
2.22
5.31
1.21
1.04
2.40
1.00
1.02
1.07
f1 (ppm)

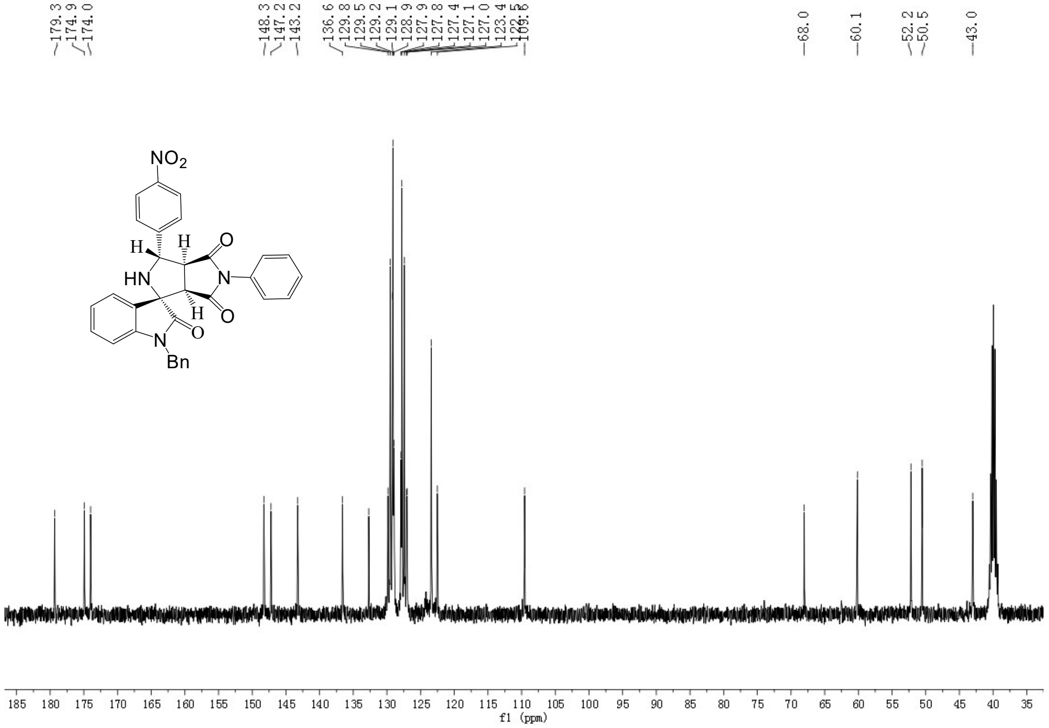
179.3
174.9
174.0
148.3
147.2
143.2
136.6
129.8
129.5
129.2
129.1
128.9
127.9
127.8
127.4
127.1
127.0
123.4
68.0
60.1
52.2
50.5
43.0
NO2
H
H
O
HN
N
H
O
N
O
Bn
f1 (ppm)

5h

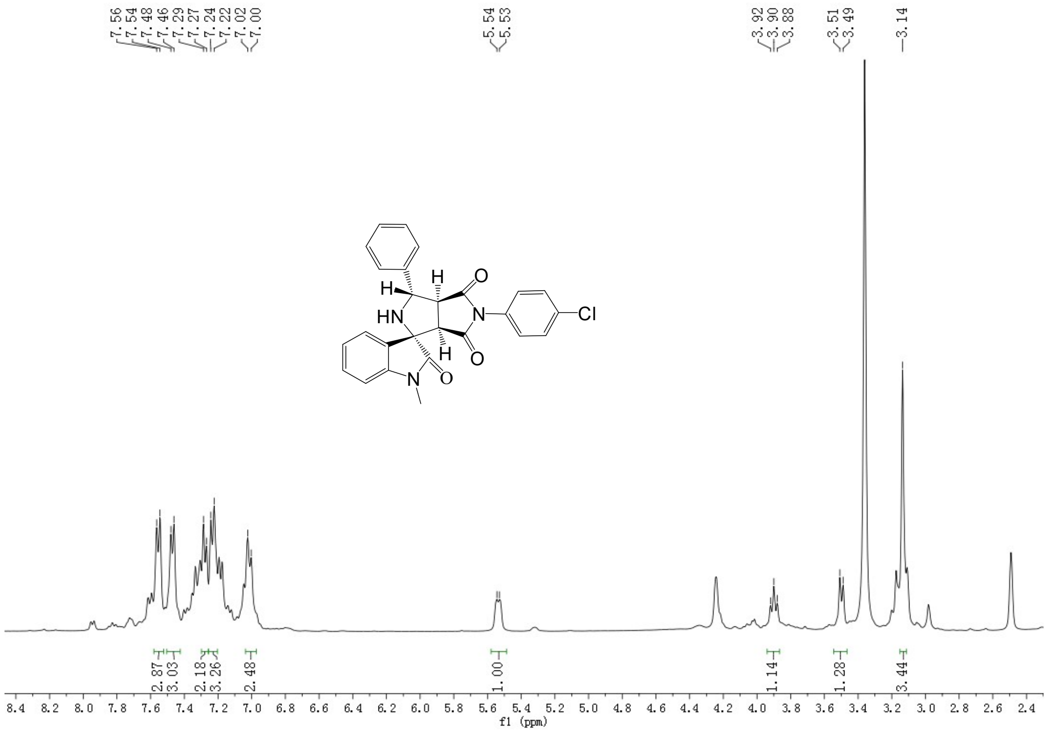
7.56
7.54
7.48
7.46
7.29
7.27
7.24
7.22
7.02
7.00
5.54
5.53
3.92
3.90
3.88
3.51
3.49
3.14
H
H
H
HN
N
N
O
O
O
Cl
2.87
3.03
2.18
3.26
2.48
1.00
1.14
1.28
3.44
8.4 8.2 8.0 7.8 7.6 7.4 7.2 7.0 6.8 6.6 6.4 6.2 6.0 5.8 5.6 5.4 5.2 5.0 4.8 4.6 4.4 4.2 4.0 3.8 3.6 3.4 3.2 3.0 2.8 2.6 2.4
f1 (ppm)

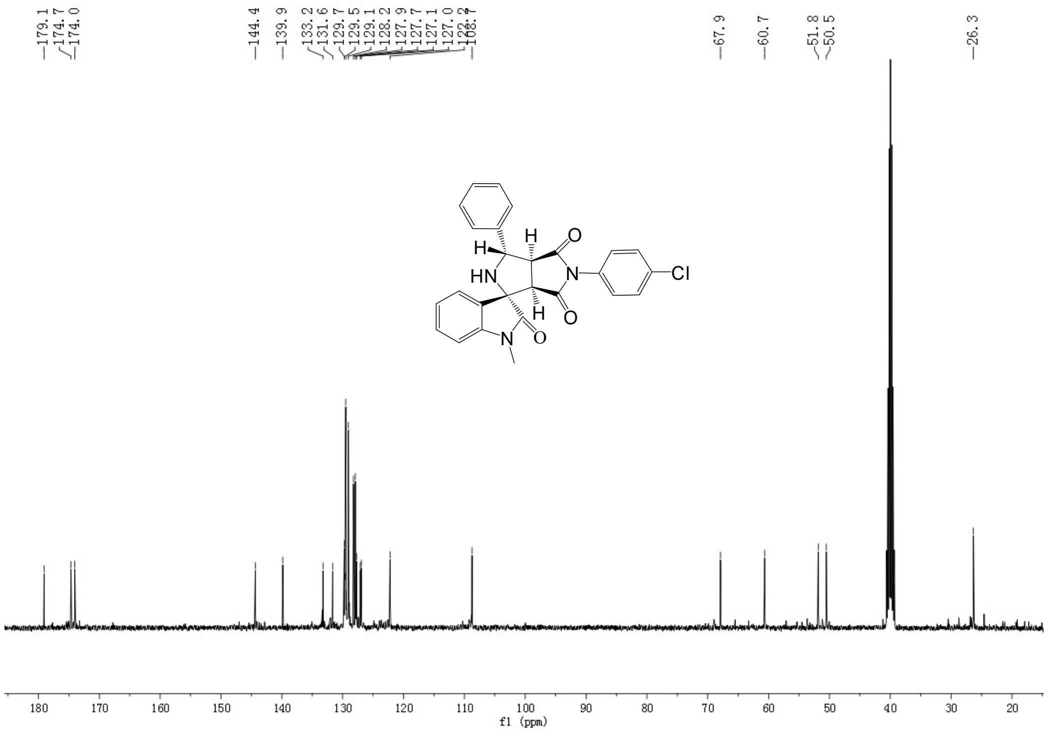
179.1
174.7
174.0
144.4
139.9
133.2
131.6
129.7
129.5
129.1
128.2
127.9
127.7
127.1
127.0
122.2
108.7
67.9
60.7
51.8
50.5
26.3
H
H
H
HN
N
N
O
O
O
Cl
180 170 160 150 140 130 120 110 100 90 80 70 60 50 40 30 20
f1 (ppm)

5i

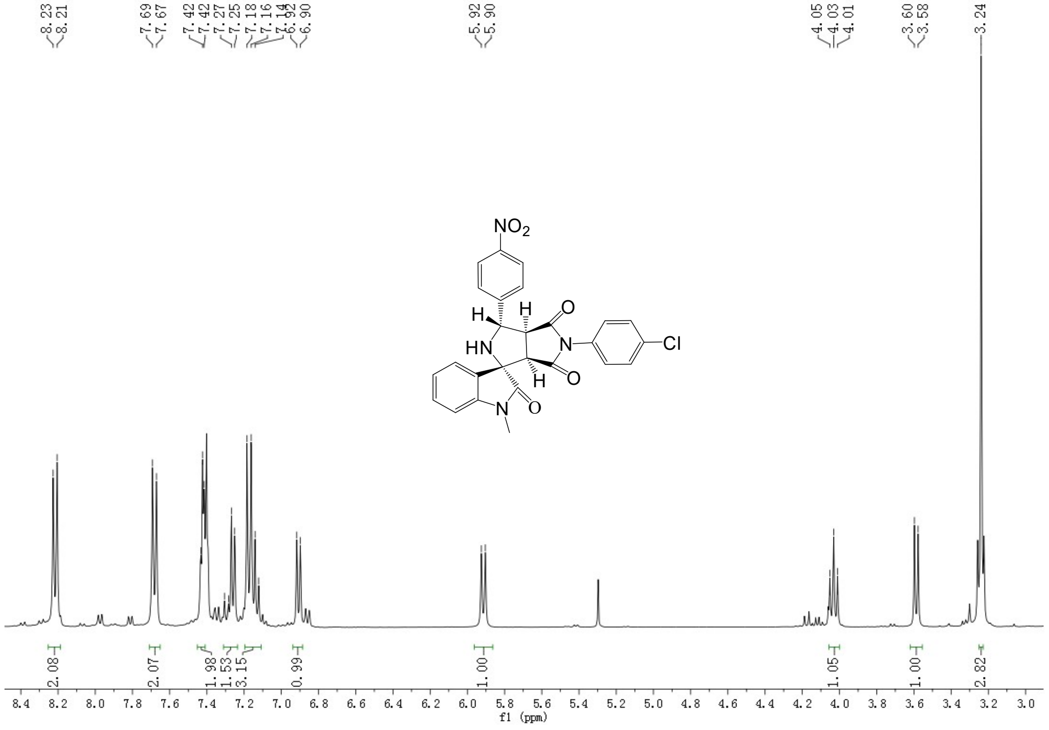
8.23
8.21
7.69
7.67
7.42
7.42
7.27
7.25
7.18
7.16
7.14
6.92
6.90
5.92
5.90
4.05
4.03
4.01
3.60
3.58
3.24
NO_2
H
H
O
HN
N
Cl
H
O
N
O
2.08
2.07
1.98
1.53
3.15
0.99
1.00
1.05
1.00
2.82
f1 (ppm)

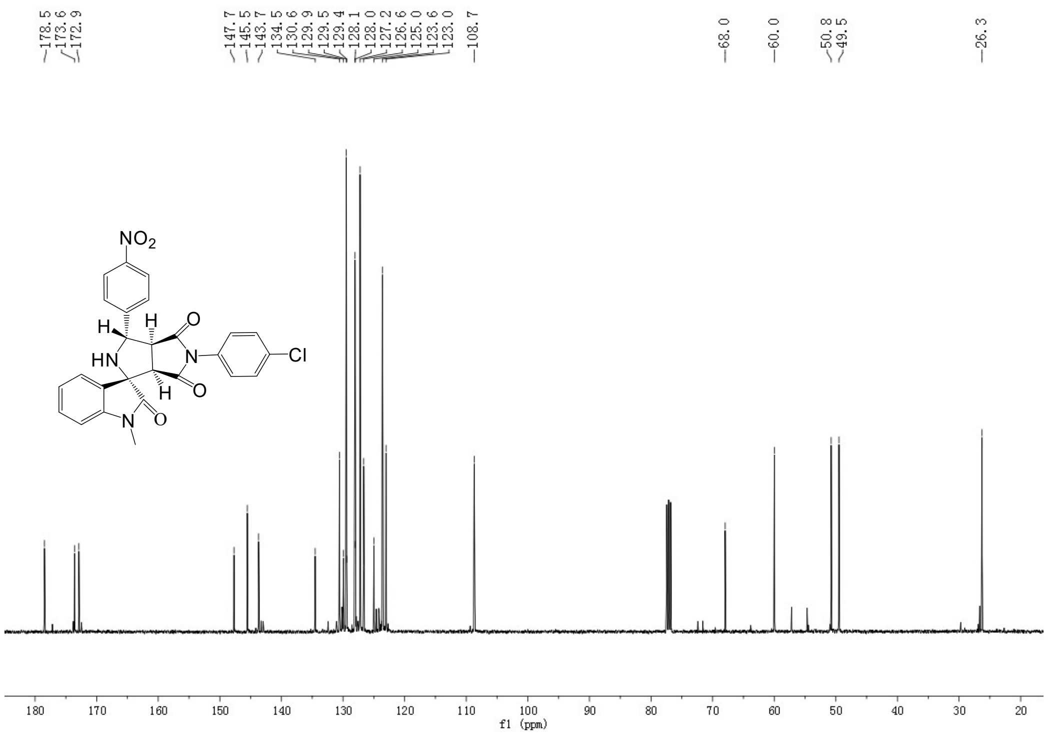
178.5
173.6
172.9
147.7
145.5
143.7
134.5
130.6
129.9
129.5
129.4
128.1
128.0
127.2
126.6
125.0
123.6
123.0
108.7
68.0
60.0
50.8
49.5
26.3
NO_2
H
H
O
HN
N
Cl
H
O
N
O
f1 (ppm)

5j

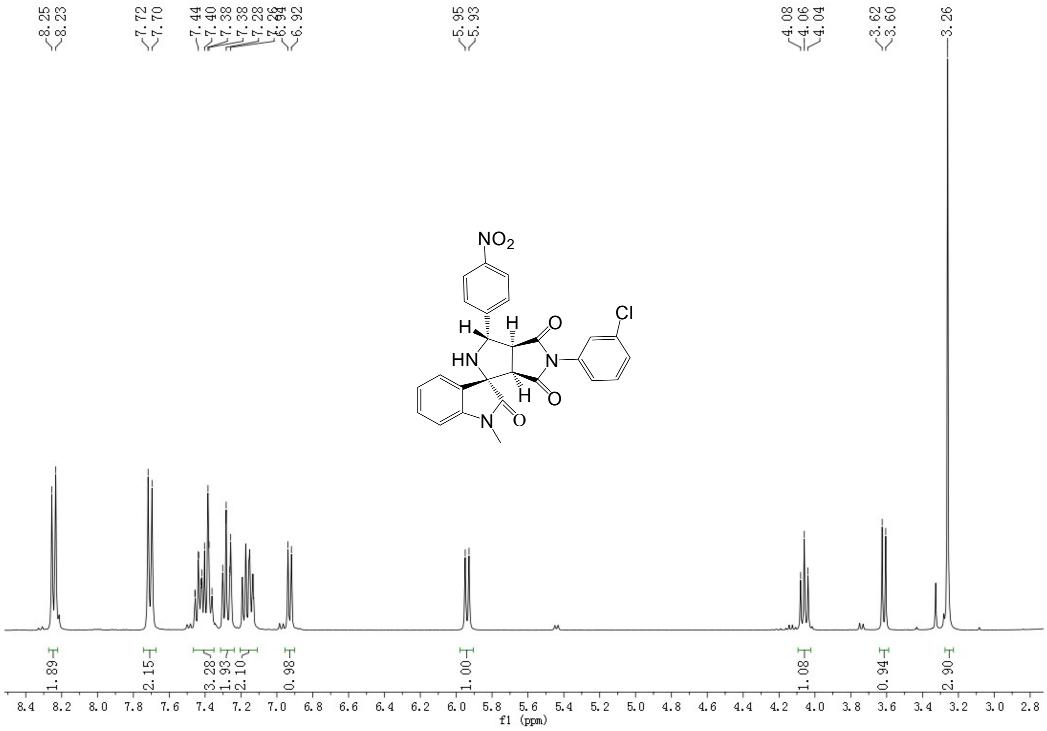
8.25
8.23
7.72
7.70
7.44
7.40
7.38
7.38
7.28
7.26
6.94
6.92
5.95
5.93
4.08
4.06
4.04
3.62
3.60
3.26
NO2
Cl
H
H
O
HN
N
H
O
N
O
1.89
2.15
3.28
1.93
2.10
0.98
1.00
1.08
0.94
2.90
f1 (ppm)

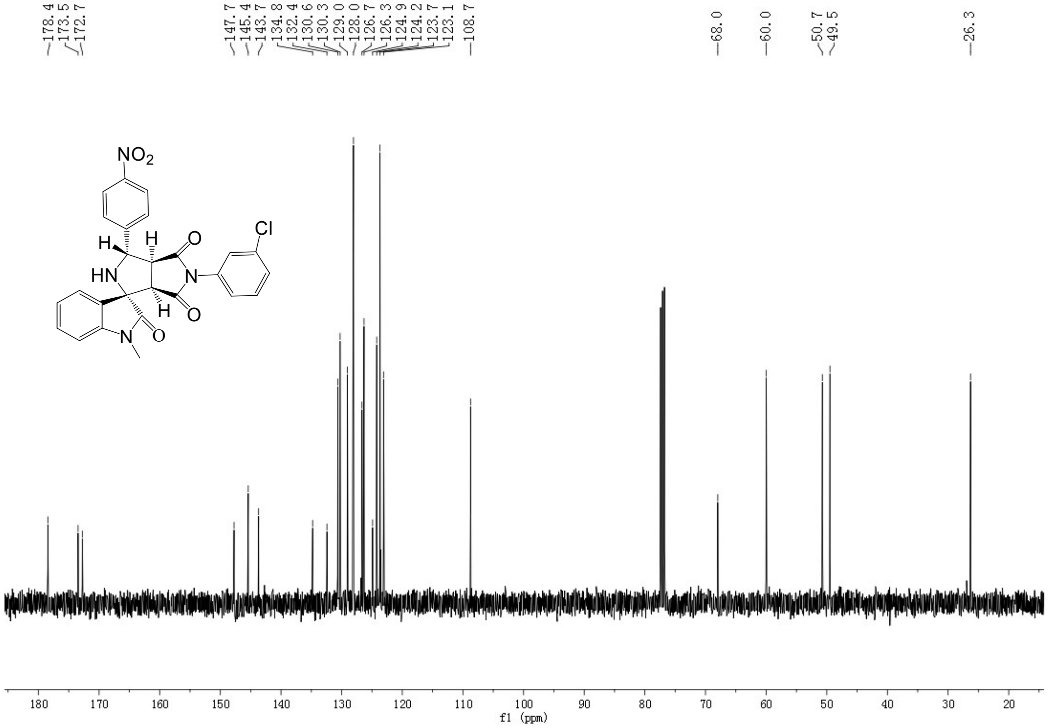
178.4
173.5
172.7
147.7
145.4
143.7
134.8
132.4
130.6
130.3
129.0
128.0
126.7
126.3
124.9
124.2
123.7
123.1
108.7
68.0
60.0
50.7
49.5
26.3
NO2
Cl
f1 (ppm)

5k

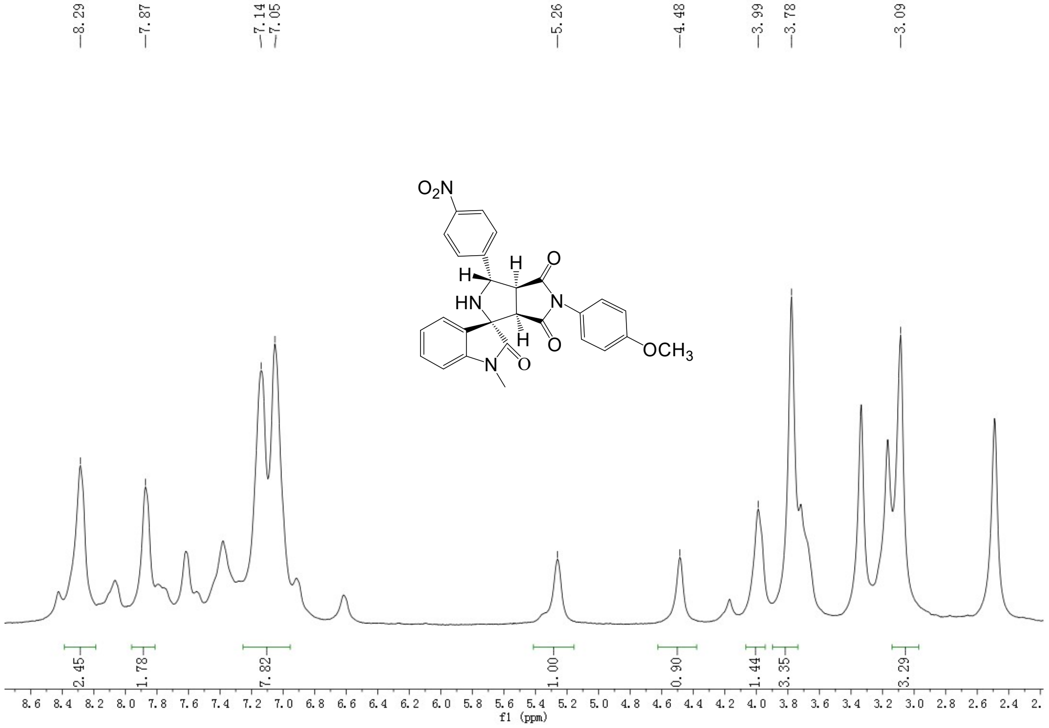

8.29
7.87
7.14
7.05
5.26
4.48
3.99
3.78
3.09
O_2N
H
H
HN
N
O
O
H
OCH3
N
O
2.45
1.78
7.82
1.00
0.90
1.44
3.35
3.29
f1 (ppm)

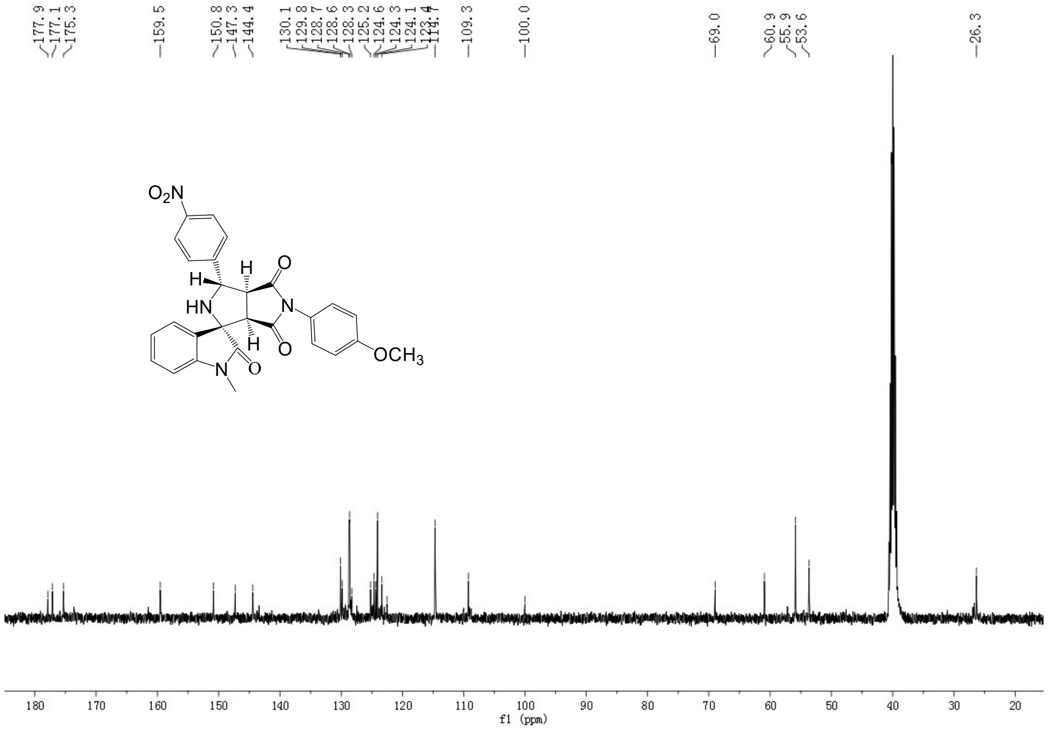

177.9
177.1
175.3
159.5
150.8
147.3
144.4
130.1
129.8
128.7
128.6
128.3
125.2
124.6
124.3
124.1
109.3
100.0
69.0
60.9
55.9
53.6
26.3
O_2N
H
H
HN
N
O
O
H
OCH3
N
O
f1 (ppm)

5l

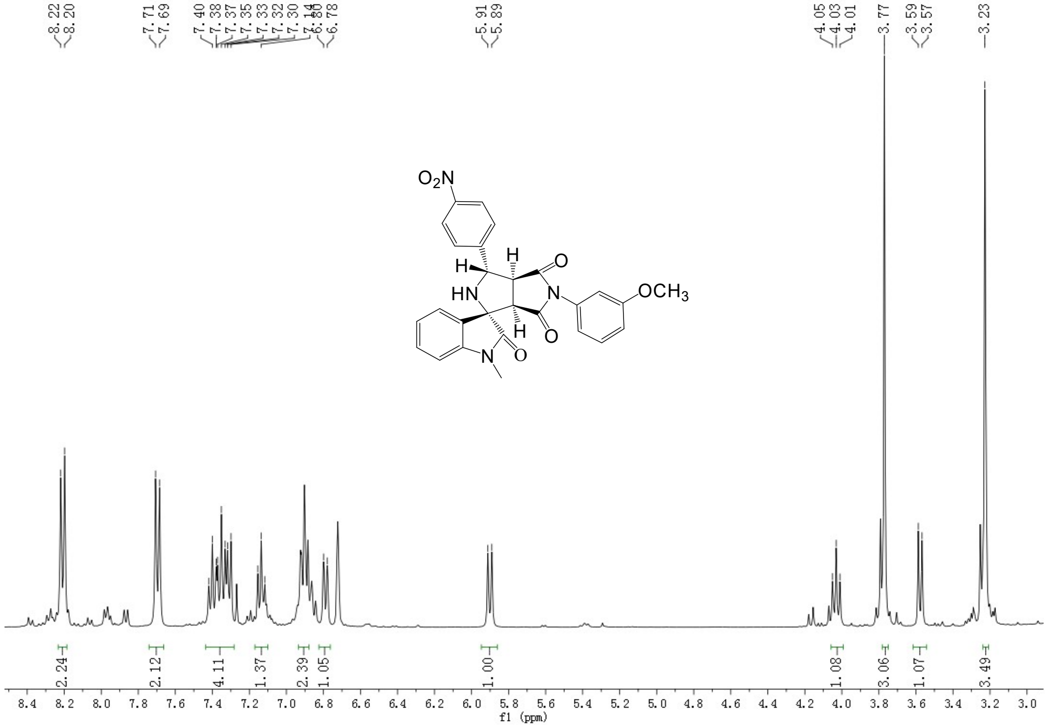
8.22
8.20
7.71
7.69
7.40
7.38
7.37
7.35
7.33
7.32
7.30
6.78
5.91
5.89
4.05
4.03
4.01
3.77
3.59
3.57
3.23
O_2N
H
HN
N
OCH_3
O
2.24
2.12
4.11
1.37
2.39
1.05
1.00
1.08
3.06
1.07
3.49
f1 (ppm)

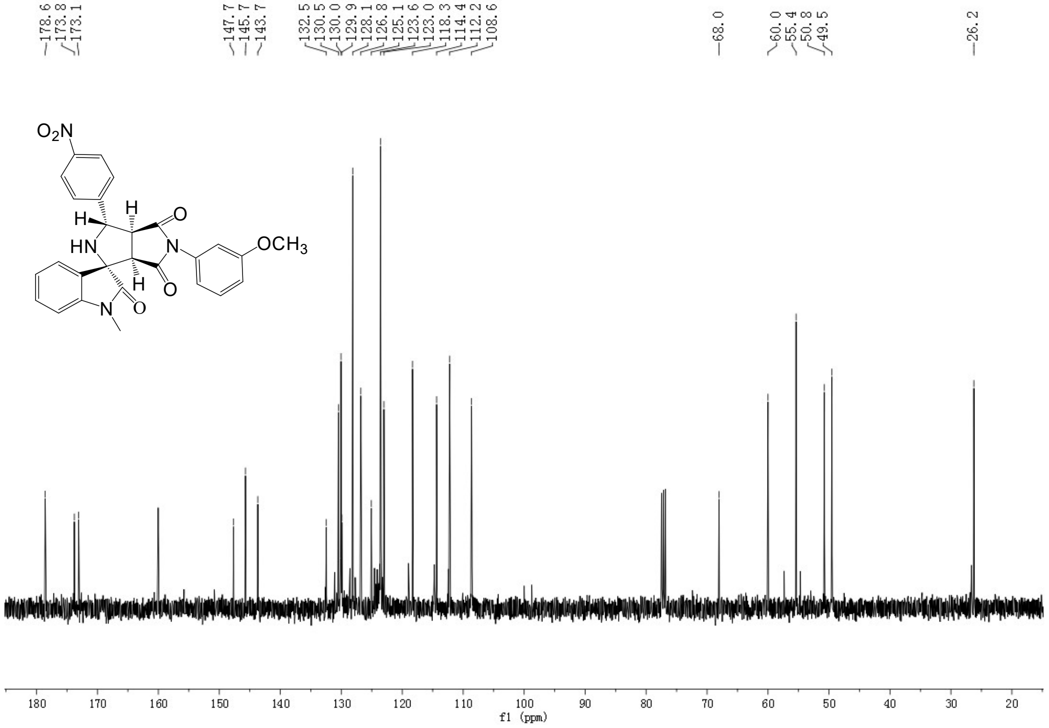
178.6
173.8
173.1
147.7
145.7
143.7
132.5
130.5
130.0
129.9
128.1
126.8
125.1
123.6
123.0
118.3
114.4
112.2
108.6
68.0
60.0
55.4
50.8
49.5
26.2
O_2N
H
HN
N
OCH_3
O
f1 (ppm)

5m

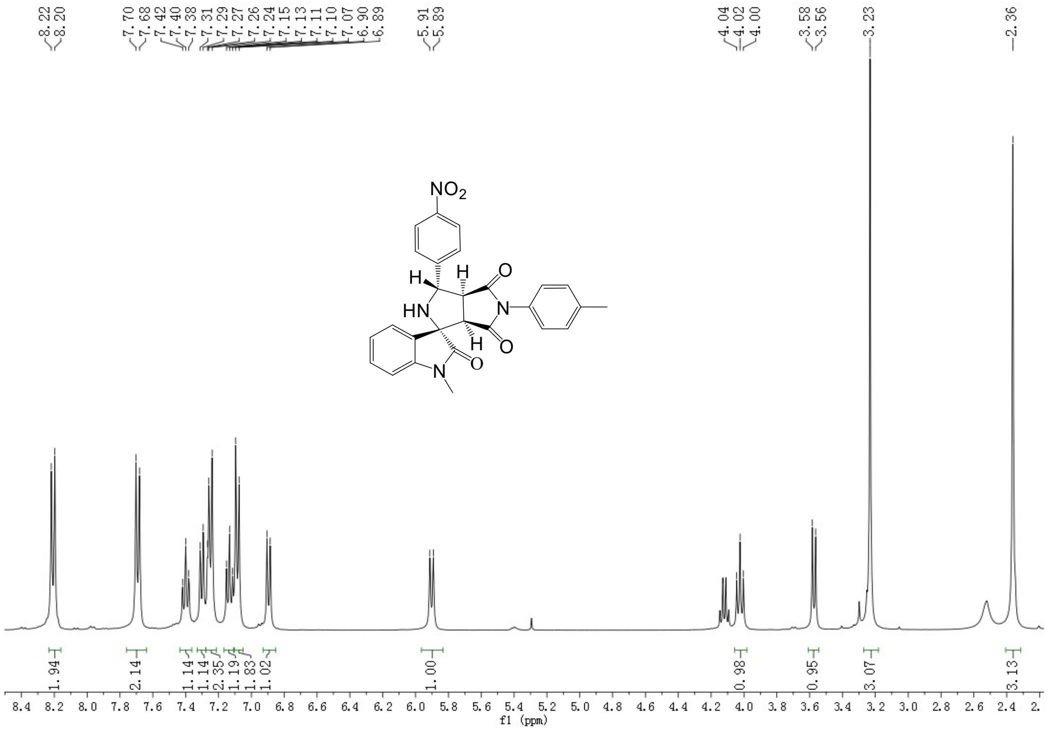

8.22
8.20
7.70
7.68
7.42
7.40
7.38
7.31
7.29
7.27
7.26
7.24
7.15
7.13
7.11
7.10
7.07
6.90
6.89
5.91
5.89
4.04
4.02
4.00
3.58
3.56
3.23
2.36
NO2
H
HN
O
N
1.94
2.14
1.14
1.14
2.35
1.19
1.83
1.02
1.00
0.98
0.95
3.07
3.13
f1 (ppm)

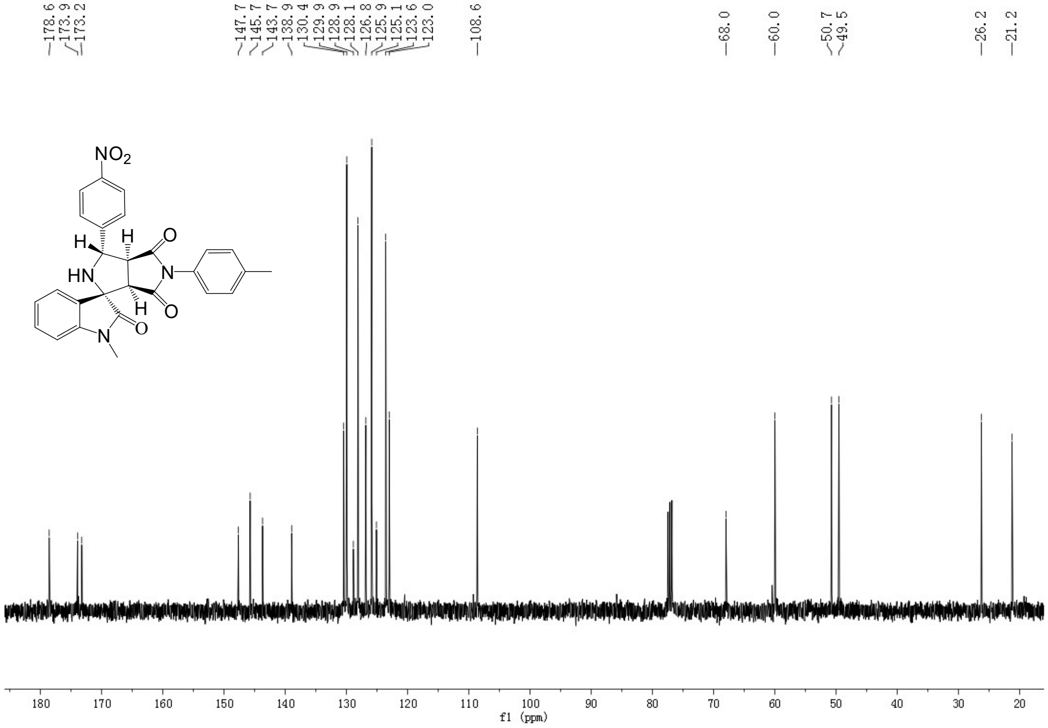

178.6
173.9
173.2
147.7
145.7
143.7
138.9
130.4
129.9
128.9
128.1
126.8
125.9
125.1
123.6
123.0
108.6
68.0
60.0
50.7
49.5
26.2
21.2
NO2
H
HN
O
N
f1 (ppm)

The crystallographic data of **4k**

Chemical Formula: C26H20BrN3O3

Molecular Weight: 502.3680

Temperature (K) 173(2)

Crystal system Monoclinic

Bond precision: C-C = 0.0042 A Wavelength=0.71073

Cell: a=10.0641(5) b=10.7045(6) c=11.0214(7)

alpha=74.935(2) beta=73.244(2) gamma=81.118(2)

Temperature: 298 K

Calculated Reported

Volume 1093.80(11) 1093.80(11)

Space group P -1 P -1

Hall group -P 1 -P 1

Moiety formula C26 H20 Br N3 O3 C26 H20 Br N3 O3

Sum formula C26 H20 Br N3 O3 C26 H20 Br N3 O3

Mr 502.35 502.36

Dx, g cm-3 1.525 1.525

Z 2 2

Mu (mm-1) 1.914 1.914

F000 512.0 512.0

F000’ 511.62

h, k, lmax 12,13,13 12,13,13

Nref 4500 4487

Tmin, Tmax 0.638,0.682 0.638,0.682

Tmin’ 0.558

Correction method= # Reported T Limits: Tmin=0.638 Tmax=0.682

AbsCorr = MULTI-SCAN

Data completeness= 0.997 Theta(max)= 26.387

R(reflections)= 0.0405(3736) wR2(reflections)= 0.1183(4487)

S = 1.071 Npar= 30

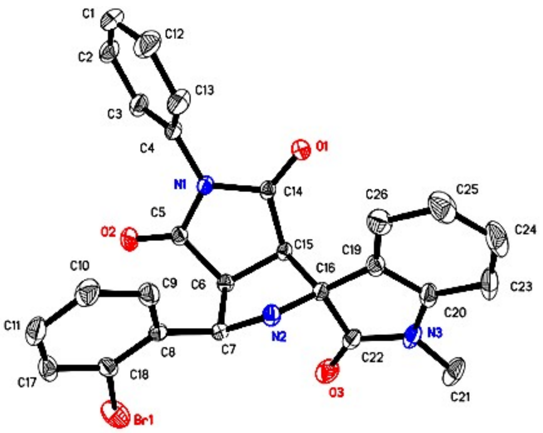

≡

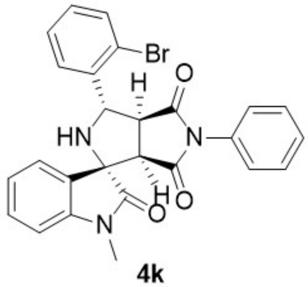

4k
